# Tersone A-G, New Pyridone Alkaloids from the Deep-Sea Fungus *Phomopsis tersa*

**DOI:** 10.3390/md17070394

**Published:** 2019-07-03

**Authors:** Shan-Chong Chen, Zhao-Ming Liu, Hai-Bo Tan, Yu-Chan Chen, Sai-Ni Li, Hao-Hua Li, Heng Guo, Shuang Zhu, Hong-Xin Liu, Wei-Min Zhang

**Affiliations:** 1 State Key Laboratory of Applied Microbiology Southern China, Guangdong Provincial Key Laboratory of Microbial Culture Collection and Application, Guangdong Open Laboratory of Applied Microbiology, Guangdong Institute of Microbiology, Guangdong Academy of Sciences, Guangzhou 510070, China; 2 School of Biosciences and Biopharmaceutics, Guangdong Pharmaceutical University, Guangzhou 510006, China; 3 Program for Natural Products Chemical Biology, Key Laboratory of Plant Resources Conservation and Sustainable Utilization, Guangdong Provincial Key Laboratory of Applied Botany, South China Botanical Garden, Chinese Academy of Sciences, Guangzhou 510650, China

**Keywords:** *Phomopsis tersa*, pyridone alkaloids, diastereoisomers, antibacterial activity, cytotoxic activity

## Abstract

Four phenylfuropyridone racemates, (±)-tersones A-C and E (**1**–**3**, **5**), one phenylpyridone racemate, (±)-tersone D (**4**), one new pyridine alkaloid, tersone F (**6**), single new phenylfuropyridone, tersone G (**7**) and two known analogs **8** and **9** were isolated from the deep-sea fungus *Phomopsis tersa*. Their structures and absolute configurations were characterized on the basis of comprehensive spectroscopic analyses, single-crystal X-ray diffraction experiments, and electronic circular dichroism (ECD) calculations. Moreover, compounds **1**–**9** were evaluated for in vitro antimicrobial and cytotoxic activity. Compounds **5b** and **8b** exhibited antibacterial activity against *S. aureus* with the MIC value of 31.5 μg/mL, while compound **5b** showed cytoxic activities against SF-268, MCF-7, HepG-2 and A549 cell lines with IC_50_ values of 32.0, 29.5, 39.5 and 33.2 μM, respectively.

## 1. Introduction

Pyridone analogues, a family of naturally occurring alkaloids, discovered from a variety of fungal sources have displayed a wide spectrum of fascinating biological activities, including cytotoxic [1,2], antibacterial [3,4,5] and antifungal [6,7] pharmacological actions, which has attracted broad attention from synthetic chemists and pharmacological communities in their search for discovering pharmaceutical drugs [8,9,10,11,12,13]. Regarding the known pyridine alkaloids, citridone A was first discovered as a potentiator for miconazole against *Candida albicans* with a unique 6-6/5/5-ring system containing a rare phenyl-*R*-furopyridone skeleton [6]. In recent years, citridone A was chemically synthesized by synthetic chemists [14,15], and these citridone derivatives were also discovered as a kind of novel bacterial-infection blocking agent to inhibit yellow pigment production in methicillin-resistant *Staphylococcus aureus* (MRSA) [15]. Hence, these new isolates can serve as promising lead compounds or clinical strategies for developing new anti-infective drugs for combating MRSA.

**Figure 1:**
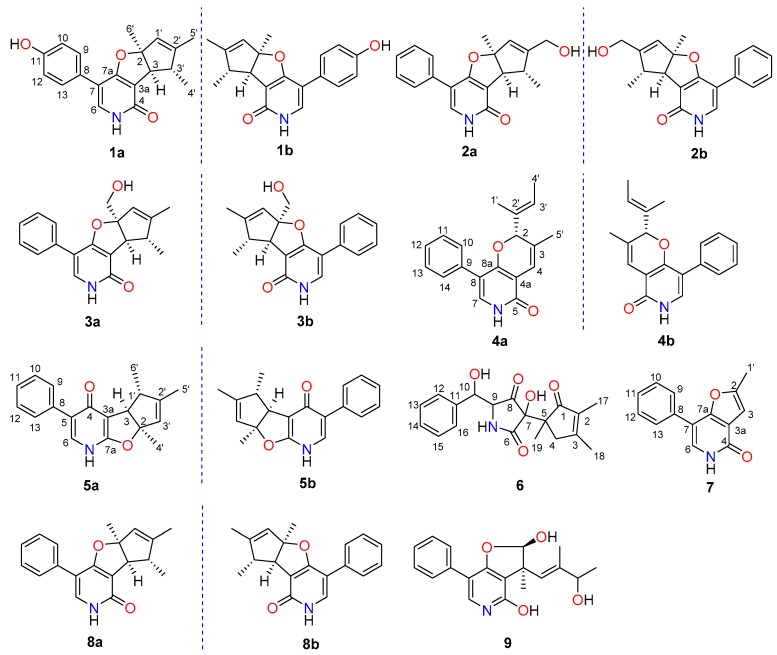
The structures of compounds **1**–**9**.

In our continuing efforts to discover structurally unique and biologically meaningful metabolites from fungal resources [16,17,18,19], the strain FS441, which was isolated from the deep-sea sediment sample, showed a remarkable diversity of secondary metabolites in the preliminary HPLC screening, and it was therefore subjected to detailed chemical analysis. As a result, seven new pyridine alkaloids tersones A (**1**)-G (**7**), along with two known derivatives citridone A [20] (**8a**), *ent*-citridone A [15] (**8b**) and CJ-16, 196 [3] (**9**) (Figure 1) were isolated from the fungus FS441. Among them, tersones A (**1**)-C (**3**) and tersones E (**5**) were isolated as phenylfuropyridone racemates with the rare naturally occurring 6-6/5/5 ring system, and tersone D (**4**) was discovered as phenylpyridone racemate with a 6-6/6 ring system. Moreover, tersone G (**7**) represented the first member of 5-phenylpyridone derivatives with an unprecedented furo [3,2-c]pyridin-4(5*H*)-one skeleton. Herein, the isolation, structural elucidation, and the bioactivity evaluations of these new compounds and the known congeners are presented.

## 2. Results and Discussion

### 2.1. Structure Elucidation

Tersone A was isolated as white needle crystals. Its molecular formula of C_19_H_19_NO_3_ was deduced from its HRESIMS *m*/*z* 310.1438 [M + H]^+^, indicating eleven degrees of unsaturation. The ^1^H NMR spectroscopic data together with the HSQC spectra of **1** indicated the presence of the critical phenylpyridone moiety based on the characteristic signals at *δ*_H_ 7.34 (1H, s, H-6), 7.32 (1H, d, *J* = 8.7 Hz, H-9), 7.32 (1H, d, *J* = 8.7 Hz, H-13), 6.79 (1H, d, *J* = 8.6 Hz, H-10), 6.79 (1H, d, *J* = 8.6 Hz, H-12), and 5.40 (1H, s, H-1′); two methines at *δ*_H_ 3.22 (2H, d, *J* = 1.8 Hz, H-3) and 2.80 (2H, q, *J* = 7.4 Hz, H-3′), as well as three methyl functional groups at *δ*_H_ 1.74 (3H, s, H_3_-5′), 1.63 (3H, s, H_3_-6′), and 1.27 (3H, d, *J* = 7.2 Hz, H-4′). The ^13^C NMR of **1** displayed 19 carbon resonances that were ascribed to an amide carbonyl functionality, twelve olefinic carbons including six quaternary carbons and six methines, one oxygen quaternary carbon, two methine and three methyl moieties. A close comparison of the NMR data of **1** (Table 1) with those of the co-isolate citridone A [20] tentatively revealed that compound **1** should also share the same phenylfuropyridone carbon skeleton with rare naturally occurring 6-6/5/5 fused ring system.

**Figure 2:**
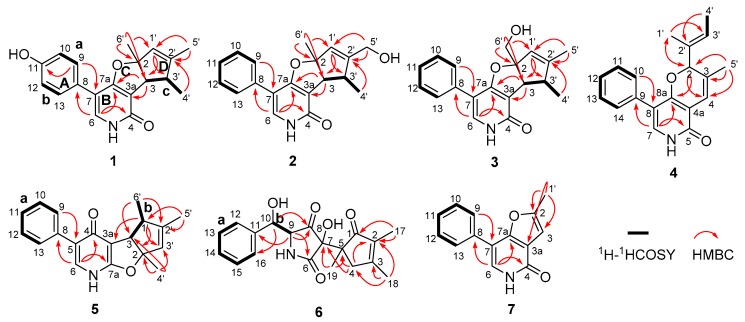
Selected ^1^H–^1^H COSY and HMBC correlations of **1**–**7**.

The planar structure of **1** was elucidated on the basis of ^1^H–^1^H COSY, HSQC, and HMBC experiments (Figure 2). The following three spin systems, **a** (C-9/C-10), **b** (C-12/C-13), and **c** (C-3/C-3′/C-4′), were successfully established and attributed to the key ^1^H–^1^H COSY correlations. Moreover, the HMBC correlations from H-9 to C-11 and C-7, H-6 to C-8, C-7a, and C-4, coupled with the fragments **a** and **b** could readily confirm the structure of rings A and B, whereas the rings C and D were rationally established by the independent spin system **c** in conjunction with the HMBC correlations from H-3 to C-7a and C-3a, H-1′ to C-3 and C-3′, as well as H-4′ to C-2′. Furthermore, the connections of C-6′ to C-2 and C-5′ to C-2′ were strongly suggested by the HMBC correlations of H_3_-6′ to C-2, C-3, C-1′ and H_3_-5′ to C-1′, C-2′, which were absolutely responsive for the location of the C-6′ and C-5′ methyl functionalities, respectively. Thus, the gross structure of **1** was unambiguously established.

**Figure 3:**
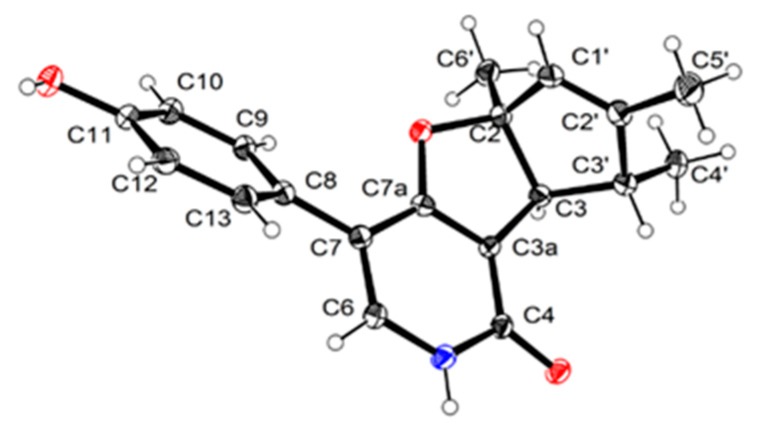
Perspective drawing of the X-ray structure of **1a**.

Compound **1** was isolated as a scalemic mixture, and the further chiral resolution by the semi-preparative HPLC fortunately separated them to afford the anticipated enantiomers with a ratio of 1.5:1, which were found to exhibit opposite optical rotations and CD cotton effects. The absolute configuration for the enantiomer **1a** was absolutely assigned by the single-crystal X-ray diffraction experiment (Figure 3). A single crystal was amazingly achieved in methanol/water solvent system, which beautifully set the stage for an efficacious X-ray diffraction analysis with a Flack parameter of 0.09(10). Then, the further X-ray diffraction experiment completely determined the whole structure and absolute configuration of **1a** as 2*S*,3*S*,3′*R*. Therefore, the absolute configuration of **1b** was finally assigned to be 2*R*,3*R*,3′*S*.

Compound **2** was isolated as white powders and assigned a positive HRESIMS ion peak at *m*/*z* 310.1446 ([M + H]^+^, calcd for C_19_H_20_NO_3_^+^), which was well matched to a molecular formula of C_19_H_19_NO_3_ with eleven degrees of unsaturation in the molecule. The comparison of the 1D NMR spectra of **2** (Table 1) with those of **1** revealed that they shared the same furopyridone carbon skeleton. One of the two obvious differences was that the methyl group at C-2′ position in **1** was oxidized to be a hydroxy one in **2**. This conclusion could be further confirmed by the HMBC correlations from H_2_-5′ to C-1′, C-2′, and C-3′. However, the other distinctive difference was that the hydroxyl group in the benzene ring A in **1** was replaced by a hydrogen atom in **2**, which was further verified by the existence of the crucial ^1^H–^1^H COSY spin system **a** (H-9/H-10/H-11/H-12/H-13). Therefore, the planar structure of **2** was established.

Likewise, compound **2** was also disclosed as a racemate. Based on the previously established methodology, a similar chiral HPLC resolution with YMC-pack Cellulose-SB column (250 × 10 mm × 5 μm) was carried out, which resulted in the successful resolution for the racemates of (±)-**2** to afford a pair of enantiomers (−)-tersone B (**2a**) and (+)-tersone B (**2b**) with a ratio of 1:1. With reference to the same 1D NMR spectral data, HRMS data, optical rotation values and relative configurations between **2a** and the synthesized one, the absolute configuration of compound **2a** was considered to be the same as that of (5a*S*,8*R*,8a*S*)-7-(hydroxymethyl)- 5a,8-dimethyl-4-phenyl-2,5a,8,8a-tetrahydro-1*H*-cyclopenta[4,5]furo[3,2-c]pyridin-1-one, which was previously reported as synthesized cirtidone derivative by Tomoda’s group [15]. Then, the configuration of **2b** was elucidated as 2*R*,3*R*,3′*S*, which could be also corroborated by the CD spectrum with positive cotton effects obviously observed at 210 and 240 nm, and a negative cotton effect at 224 nm. Therefore, the absolute configuration of **2** was also logically established.

Tersone C was obtained as white cubic crystals, and its molecular formula was assigned as C_19_H_19_NO_3_ based on the HRESIMS at *m*/*z* 310.1435 [M + H]^+^ (calcd C_19_H_20_NO_3_^+^). The ^1^H and ^13^C NMR data of **3** (Table 2) closely resembled those of **2**, except for the oxidation of the methyl group into a hydroxymethyl one at C-2 position in **3** and the replacement of the hydroxymethyl functionality at C-2′ position in **2** by a methyl one in **3**. The aforementioned deduction could be further strengthened by the HMBC correlations from H_2_-6′ to C-1′, C-2, and C-3, as well as H_3_-5′ to C-1′, C-2′ and C-3′.

**Figure 4:**
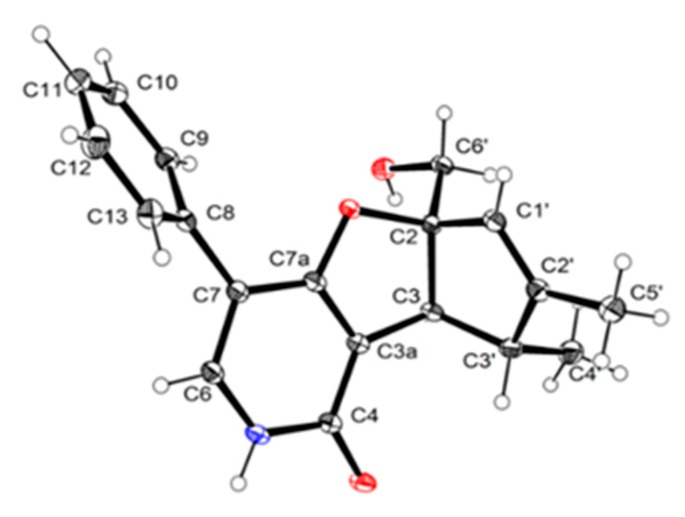
Perspective drawing of the X-ray structure of **3**.

The relative configuration of **3** was determined by a single-crystal X-ray diffraction experiment using CuK*α* radiation (Figure 4). Further efforts were performed to determine the absolute configuration of compound **3**. Apparently, compound **3** might also be an enantiomeric racemate as it was found to lack the essential optical rotation and circular dichroism effect. Subsequent chiral separation through the semi-preparative HPLC using a YMC-pack Cellulose-SB column (250 × 10 mm × 5 μm) to afford a pair of optically pure enantiomers (−)-tersone C (**3a**) and (+)-tersone C (**3b**) with a ratio of approximately 1:1. The absolute configurations for the enantiomers **3a** and **3b** were successfully established by a close comparison of their CD spectra and optical rotation values with those of compounds (−)-**2a** and (+)-**2b**. Both of them exhibited the characteristic optical rotations and positive cotton effects at 210, 230, and 245 nm in the CD spectra, which shared a very high similarity with compounds (−)-**2a** and (+)-**2b**, respectively. Consequently, the absolute configurations of **3a** and **3b** were conclusively assigned as 2*S*,3*S*,3′*R* and 2*R*,3*R*,3′*S*.

Tersone D was also purified as white powders, and its molecular formula was determined as C_19_H_19_NO_2_, which was established by the HRESIMS data as a positive mode at *m*/*z* 294.1496 [M + H]^+^ (calcd for C_19_H_20_NO_2_^+^), indicative of eleven indices of hydrogen deficiency. The analyses of the NMR data (Table 2) of **4** suggested the presence of three methyl groups (*δ*_C_ 11.7, 13.3 and 19.6), nine methines carbons including one oxygenated (*δ*_C_ 87.3) and eight olefinic ones (*δ*_C_ 115.1, 134.6, 129.3, 130.0, 128.5, 130.0, 129.3 and 126.2), as well as seven quaternary carbons, including one ketone carbonyl (*δ*_C_ 162.5), and six olefinic ones (*δ*_C_ 129.7, 108.3, 116.6, 160.8, 134.9 and 133.9).

The HMBC correlations from H-10 to C-8, H-7 to C-9, C-8a and C-5 along with the ^1^H-^1^H COSY fragment of H-10/H-11/H-12/H-13/H-14 collectively pointed to the existence of the key phenylpyridone core. The comparison of the NMR spectroscopic profiles of compound **4** with those of citridone E [5], which was previously confirmed by single crystal X-ray diffraction, revealed close structure similarities with regard to the 6-6/6 ring systems and substituent patterns. The only difference was attributable to the *trans* double bond at C-3 and C-4 positions in **4** which was replaced by a carboxyl functional moiety in citridone E. This deduction was further confirmed by the chemical shifts of C-2′ [*δ*_C_ 133.9] and C-3′ [*δ*_C_ 126.2, *δ*_H_ 5.61 (1H, q, *J* = 6.2 Hz,)] in conjunction with key HMBC correlations from the H_3_-1′ to C-2 and C-3′, H_3_-4′ to C-2′ and C-3′. Moreover, the chemical shifts of C-3 [*δ*_C_ 129.7] and C-4 [*δ*_C_ 115.1, *δ*_H_ 6.50 (1H, s)] as well as the HMBC correlations from H_3_-5′ to C-2, C-3, C-4 and H-2 to C-4 further consolidated this conclusion.

**Scheme 1:**
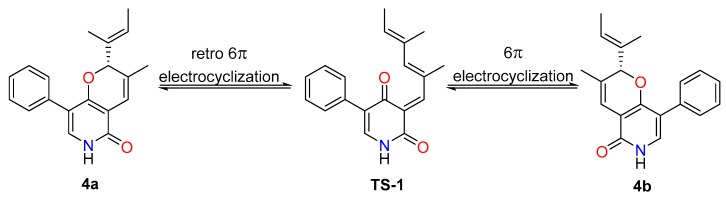
The potential inter-transformation between **4a** and **4b**.

**Figure 5:**
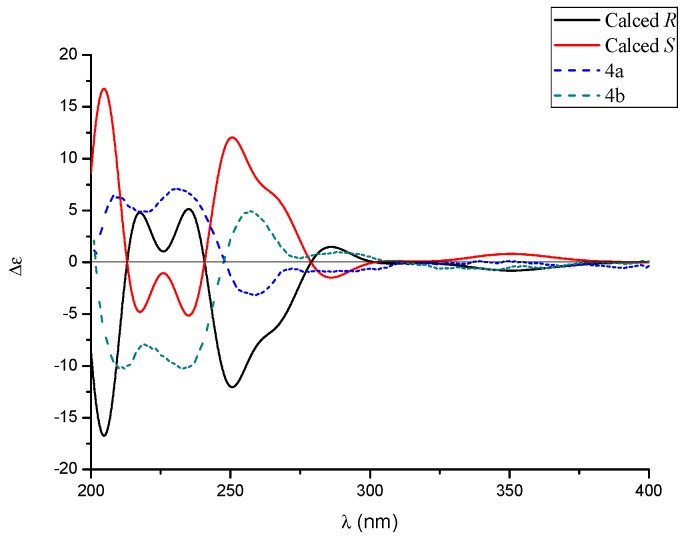
The experimental ECD curves of **4a** (solid black line) and **4b** (solid red line).

In analogy with compounds **1**–**3**, the small specific rotation value and extraordinarily weak CD cotton effect of **4** implied that it was also a racemic mixture. Interestingly, subsequent chiral-phase HPLC separation successfully accessed the two anticipated enantiomers (+)-tersone D (**4a**) and (−)-tersone D (**4b**), which were stable at room temperature and showed opposite specific rotation values and electronic circular dichrosim (ECD) curves mutually. However, the potential spontaneous retro 6π electrocyclization/6π electrocyclization cascaded reaction tended to make them as an unseparated enantiomers (as shown in Scheme 1). By comparing the ^13^C NMR data of citridone E and citridone F in the literature [5], it was confirmed that the double bond at positions C-2′ of compound **4** was the *E* configuration. In order to unravel their absolute structures, efforts toward conclusive evidence by comparison of the experimental and calculated circular dichroism (CD) spectra using the time-dependent DFT were performed. As depicted in Figure 5, the calculated ECD spectra closely matched the experimental ones, which set the stage for their further absolute structure determinations. Therefore, the absolute configurations of **4a** and **4b** were clarified as 2*R* and 2*S* respectively, and the compound **4** was given the trivial name, tersone D.

Compound **5** was a yellow powder, its molecular formula was assigned as C_19_H_19_NO_2_ with eleven indices of hydrogen deficiency by the positive HRESIMS ion at *m*/*z* 294.1497 [M + H]^+^ (calcd 294.1489). The planar structure of **5** was ultimately determined by analysis of its NMR spectra (Table 3) involving HSQC, ^1^H–^1^H COSY, and HMBC correlations. The ^1^H–^1^H COSY spectrum of **5** displayed two proton spin−spin systems in the core structure affiliated to **a** (H-9/H-10/H-11/H-12/H-13) and **b** (H-3/H-1′/H_3_-6′) as depicted with bold lines in Figure 2. Moreover, the HMBC correlations from H-6 to C-4, C-7a, and C-8 and H-9 to C-5 coupled with the fragment **a** successfully which helped to reveal the presence of the 3-phenylpyridin-4(1H)-one scaffold. Futhermore, the fragment **b** together with the HMBC correlations from H-1′ to C-2, C-3′ and C-3a, from H_3_-6′ to C-3 and C-2′, from H-4′ to C-2, C-3 and C-3′ and from H-5′ to C-1′ and C-2′, unambiguously constructed the cyclopentadienyl furan ring system. Then, the planar structure of **5** was established as shown in Figure 1.

**Figure 6:**
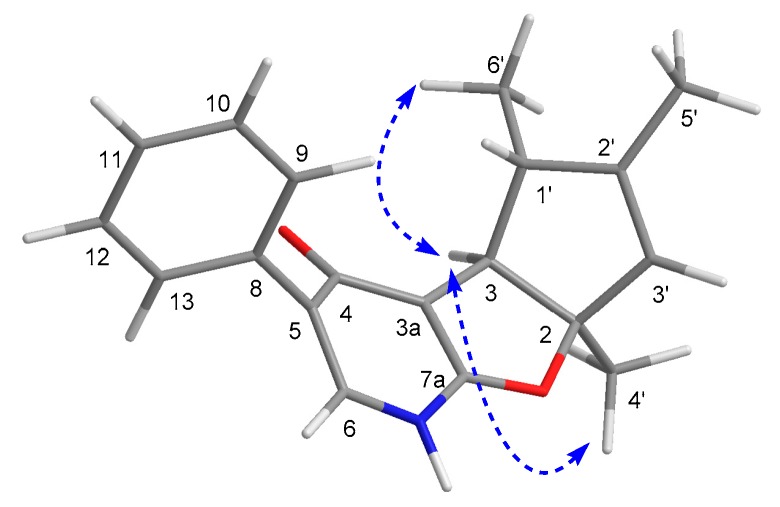
Key NOESY correlations of **5**.

The relative configuration of **5** was established by the NOESY experiment, wherein the NOE correlations between H_3_-4′ to H-3 and H_3_-6′ to H-3 indicated that the protons of H-3, H_3_-4′, and H_3_-6′ ought to be oriented in the same direction (Figure 6). Furthermore, the chiral-phase HPLC analysis of **5** revealed it to be also a racemate, and the further semi-preparative chiral HPLC separation successfully resulted in the resolution of the two corresponding enantiomers (−)-tersone E (**5a**) and (+)-tersone E (**5b**). The compound **5b** was previously synthesized by Tomoda’s group [15], which was confirmed by the comparison of 1D NMR spectral data, HRMS data and the optical rotation values with those of the synthesized one. Finally, the absolute configuration of **5b** was established to be the same as (4b*S*,5*R*,7a*S*)-5,6,7a-trimethyl-3-phenyl-4b,5-dihydro-1*H*-cyclopenta[4,5]furo[2,3-b]pyridin-4(7a*H*)-one [15], which was defined as 2*S*,3*S*,3′*R* in this study. Therefore, the absolute stereochemistry of **5a** could be further determined as 2*R*,3*R*,3′*S*.

Tersone F was isolated as white powders. Its HRESIMS data was disclosed by the obvious positive ion peak at *m*/*z* 344.1494 [M + H]^+^ in the HRESIMS spectrum, and it corresponded to a molecular formula of C_19_H_21_NO_5_. The ^1^H NMR data of **6** (Table 3) in conjunction with its HSQC spectrum displayed a series of characteristic resonances responsive for three methyls [*δ*_H_ 1.57 (3H, s, H_3_-17), 2.04 (3H, s, H_3_-18), and 1.25 (3H, s, H_3_-19)], two methines [*δ*_H_ 4.13 (1H, d, *J* = 2.9 Hz, H-9) and 5.14 (1H, d, *J* = 2.9 Hz, H-10)], and five olefinic proton signals at [*δ*_H_ 7.39 (1H, d, *J* = 6.9 Hz, H-12), 7.37 (1H, t, *J* = 6.9 Hz, H-13), 7.28 (1H, t, *J* = 6.9 Hz, H-14), 7.37 (1H, t, *J* = 6.9 Hz, H-15), and 7.39 (1H, d, *J* = 6.9 Hz, H-16)].

The planar structure of **6** was defined by the analysis of its 2D NMR spectra. Firstly, the COSY and HSQC correlations revealed the presence of the two structural fragments, **a** (C-12/C-13/C-14/C-15/C-16) and **b** (C-9/C-10). Secondly, the HMBC correlations from H-10 to C-8, C-11, and C-16 as well as H-9 to C-6, C-8 and C-11, coupling with the fragments **a** and **b**, led to tentative establishment of the connection between the mono-substituted benzene ring and the pyrrolidine-2,4-dione ring. Moreover, the cyclopent-2-en-1-one subunit was constructed by the cross peaks from H_2_-4 to C-2, C-5 and C-7, H_3_-17 to C-1 and C-2, H_3_-18 to C-2, C-3 and C-4, as well as H_3_-19 to C-5. These aforementioned informative results collectively indicated that compound **6** was structurally related to citridone C [20], excepting the obvious chemical shift changes at C-7 and C-8 positions. With a careful inspection and interpretation of their NMR data, the major difference between them was that the quaternary enol carbons at C-7 and C-8 positions in citridone C were replaced by two quaternary carbons bearing hydroxyl and carbonyl functionalities, respectively. The deduction was evidenced by the chemical shift of C-7 [*δ*_C_ 76.1], C-8 [*δ*_C_ 208.9] as well as the HMBC correlations from H-9 to C-8, H-10 to C-8, and H_3_-19 to C-7. Therefore, the planar structure of **6** was completely interpreted as shown in Figure 1. The relative configuration of **6** was not determined because the active protons of OH and NH had a lack of signals in the ^1^H NMR and NOESY spectra.

Compound **7** was obtained as a yellow powder, and its molecular formula was established as C_14_H_11_NO_2_ with nine indices of hydrogen deficiency based on the HRESIMS with a positive deprotonated molecular ion [M + H]^+^ at *m*/*z* 226.0867 [M + H]^+^ (C_14_H_12_NO_2_^+^, calcd for 226.0863). The 1D NMR (Table 4) and HSQC spectroscopic data of **7** revealed the existence of fourteen carbon resonances ascribable to one methyl (*δ*_C_ 13.6), seven olefinic methines (*δ*_C_ 103.3, 128.6, 128.6, 129.9, 128.9, 129.9, and 128.6), and six quaternary carbons (*δ*_C_ 156.4, 118.4, 113.2, 161.6, 133.9 and 159.6). The above-mentioned information indicated that compound **7** might be also a phenylpyridone derivative. The HMBC correlations from H-6 to C-4, C-7a and C-8, H-9 to C-7 coupling with the critical COSY fragment H-9/H-10/H-11/H-12/H-13, confirmed the construction of the 5-phenylpyridone moiety. Additionally, the HMBC correlations from H_3_-1′ to C-2, C-3 and C-3a, and H-3 to C-3a and C-7a suggested the presence of the 2-methylfuran moiety. Therefore, the structure of **7** was finally established, which represented the first member of 5-phenylpyridone derivatives with an unprecedented furo[3,2-c]pyridin-4(5*H*)-one skeleton as shown in Figure 1, and given the trivial name tersone G.

In addition, the known phenylpyridone analogues were elucidated as citridone A [20] (**8a**), *ent*-citridone A [15] (**8b**) and CJ-16, 196 [3] (**9**) by comparison of their NMR spectra, MS data, and specific rotation values with those of the reported literature.

### 2.2. Biological Activity

The minimum inhibitory concentration (MIC) for compounds **1**–**9** against bacteria *Staphylococcus aureus* ATCC 29,213 and *Escherichia coli* ATCC 8739 was evaluated by means of the microdilution susceptibility test, and performed in Mueller−Hinton broth (MHB) following CLSI guidelines by using the vancomycin as the positive control. Compounds **5b** and **8b** exhibited antibacterial activity against *S. aureus* with the MIC value of 31.5 μg/mL. However, compounds **1**–**3**, **6**–**7**, and **9** were found to be devoid of significant activity (Table 5).

Additionally, compounds **1**–**9** were also tested for their cytotoxic activities against four human tumor cell lines (SF-268, MCF-7, HepG-2 and A549). As a result, **5b** showed cytotoxic activities against SF-268, MCF-7, HepG-2 and A549 cell lines with IC_50_ values of 32.0, 29.5, 39.5 and 33.2 μM, respectively, while the IC_50_ values of the positive control drug cisplatin against the four cell lines were 3.0, 2.6, 2.4, 1.9 μM, respectively. The other compounds were inactive with IC_50_ values more than 100 μM.

## 3. Materials and Methods

### 3.1. General Experimental Procedures

Optical rotations were determined with an Anton Paar MCP-500 spectropolarimeter (Anton Paar, Graz, Austria). Circular dichroism (CD) spectra were recorded under N_2_ gas on a Jasco 820 spectropolarimeter (Jasco Corporation, Kyoto, Japan). IR data were measured by a Shimadzu IR Affinity-1 spectrometer (Shimadzu, Kyoto, Japan). UV spectra were acquired using a Shimadzu UV-2600 spectrophotometer (Shimadzu, Kyoto, Japan). ESIMS data were collected on an Agilent Technologies 1290-6430A Triple Quad LC/MS (Agilent Technologies, Palo Alto, CA, USA). HRESIMS were done with a Thermo MAT95XP (Thermo Fisher Scientific, Bremen, Germany). The melting point was determined on a Yanagimoto Seisakusho MD-S2 micro-melting point apparatus and was uncorrected. NMR spectra were obtained by a Bruker Avance-600 spectrometer (Bruker, Fällanden, Switzerland). Preparative HPLC was performed using a YMC-pack ODS-A column (250 × 20 mm, 5 μm, 12 nm, YMC Co., Ltd., Kyoto, Japan). A YMC-pack ODS-A/AQ column (250 × 10 mm, 5 μm, 12 nm, YMC CO., Ltd., Kyoto, Japan) was used for semipreparative HPLC separation, the YMC-pack Cellulose-SB column (250 × 10 mm, 5 μm, 12 nm, YMC CO., Ltd., Kyoto, Japan) and the CHIRALPAK IC column (250 × 10 mm, 5 μm) for chiral semipreparative HPLC separation. Silica gel (100–200 mesh and 200–300 mesh, Qingdao Marine Chemical Inc., Qingdao, China), C_18_ reversed-phase silica gel (40–63 μm, Merck, Darmstadt, Germany), and Sephadex LH-20 gel (Pharmacia Fine Chemical Co. Ltd., Uppsala, Sweden) were used in the chromatography processes. Fractions were monitored by TLC and spots were detected on heated TLC plates (silica gel GF_254_ plates, Qingdao Marine Chemical Inc., Qingdao, China) with 10% H_2_SO_4_ in EtOH under UV light.

### 3.2. Fungal Material

The fungal strain FS441 used in this work was isolated from a sediment sample, which was collected from the depth of 3000 m in the Indian Ocean (88°58′640″ E, 0°00′307″ S), in April 2016. The isolated strain was deposited in the Guangdong Provincial Key Laboratory of Microbial Culture Collection and Application, Guangdong Institute of Microbiology. The strain was identified by using BLAST (nucleotide sequence comparison program) to search the GenBank database, which had 98.9% similarity to *Phomopsis tersa* SYJM09 (GenBank Accession No. JF923840). Hence, the strain was identified as *Phomopsis tersa* (GenBank Accession No. MK592793).

### 3.3. Fermentation and Extraction

The marine fungus *P. tersa* FS441 was cultured on potato dextrose agar (PDA) at 28 °C for 7 days to prepare the seed culture, and then inoculated into flasks (3 L) containing 9 g sea salts, 250 g of rices and 300 mL of waters. After that, all flasks were incubated at 28 °C for one month and the fermented rice substrate was extracted repeatedly with EtOAc. After the evaporation of the solvent, a dark brown solid (177.9 g) was obtained. The crude extract was fractionated by silica gel column chromatography (100–200 mesh) with two gradient systems of increasing polarity (petroleum ether/EtOAc, 10:1→1:1; CH_2_Cl_2_/CH_3_OH, 10:1→0:1) to furnish seven fractions (A-G).

Fraction E (41.8 g) was subjected to C-18 reversed-phase silica gel CC (gradient elution with MeOH-H_2_O, 30:70→100:0) to afford fifteen subfractions (E1-E15). E6 was separated by Sephadex LH-20 CC (CH_2_Cl_2_-MeOH, 1:1) to yield two subfractions (E6.1-E6.2). E6.2 was subjected to preparative HPLC (MeOH-H_2_O, 60:40, 6 mL/min) to afford eight subfractions (E6.2.1-E6.2.8). E6.2.3 and E6.2.4 were purified by semi-preparative HPLC (MeCN-H_2_O, 30:70, 3 mL/min), and further purified by semi-preparative HPLC equipped with a chiral column (Isopropanol-Hexane, 30:70, 3 mL/min) to yield **6** (2.5 mg, *t_R_* = 7.0 min) and **9** (17.4 mg, *t_R_* = 13.0 min), respectively.

E7 was separated by preparative HPLC (MeOH-H_2_O, 70:30, 6 mL/min) to obtain thirteen subfractions (E7.1-E7.13). **2b** (0.5 mg, *t_R_* = 13.2 min) and **2a** (0.5 mg, *t_R_* = 13.8 min) were obtained from E7.9 by analysis of semi-preparative HPLC (MeCN-H_2_O, 35:65, 3 mL/min) and semi-preparative HPLC equipped with a chiral column (MeCN-H_2_O, 50:50, 2 mL/min). Then, semi-preparative HPLC (MeCN-H_2_O, 50:50, 3 mL/min) and semi-preparative HPLC equipped with a chiral column (MeCN-H_2_O, 70:30, 2 mL/min) analysis of E7.12 afforded **3b** (1 mg, *t_R_* = 8.0 min) and **3a** (1 mg, *t_R_* = 10.0 min), respectively.

E8 was divided into four subfractions (E8.1-E8.4) by silica gel CC (CH_2_Cl_2_-MeOH, 30:1→1:1). Further, **7** (0.9 mg, *t_R_* = 14.0 min) was obtained from E8.2 followed by semi-preparative HPLC (MeCN-H_2_O, 50:50, 3 mL/min) and semi-preparative HPLC equipped with a chiral column (Isopropanol-Hexane, 50:50, 3 mL/min), respectively. E8.3 was purified by preparative HPLC (MeOH-H_2_O, 62:38, 6 mL/min), and further purified by semi-preparative HPLC equipped with a chiral column (Isopropanol-Hexane, 30:70, 3 mL/min) to offer **1a** (2.3 mg, *t_R_* = 16.0 min) and **1b** (1.4 mg, *t_R_* = 14.0 min), respectively.

E9 was separated into six subfractions (E9.1-E9.6) by silica gel CC (CH_2_Cl_2_-MeOH, 40:1-10:1), then E9.1 was further divided into twenty parts (E9.1.1-E9.1.20) by semi-preparative HPLC (MeCN-H_2_O, 50:50, 3 mL/min). Semi-preparative HPLC equipped with a chiral column (Isopropanol-Hexane, 30:70, 3 mL/min) analysis of E9.1.8 afforded **5a** (8.6 mg, *t_R_* = 24.0 min), **5b** (7.8 mg, *t_R_* = 27.0 min), E9.1.8.1 and E9.1.8.4-E9.1.8.5, respectively. E9.1.8.4 and E9.1.8.5 were further purified by semipreparative HPLC (MeCN-H_2_O, 60:40, 2 mL/min) to get **4a** (0.6 mg, *t_R_* = 22.0 min) and **4b** (0.5 mg, *t_R_* = 20.0 min), respectively. **8b** (1.9 mg, *t_R_* = 14.0 min) and **8a** (2 mg, *t_R_* = 17.0 min) were obtained from E9.3 followed by semi-preparative HPLC (MeCN-H_2_O, 70:30, 2 mL/min) and semi-preparative HPLC equipped with a chiral column (Isopropanol-Hexane, 60:40, 3 mL/min), respectively.

(−)-Tersone A (**1a**): Mp 179–180 °C; white needle crystals; ${\left[\alpha \right]}_{D}^{25}$–45.6 (*c* 0.09, MeOH). CD (0.2 mg/mL, MeOH): 217 (−10.84), 246 (−5.13) nm. UV (MeOH) *λ*_max_ (log *ε*): 256 (4.13) nm. IR *ν*_max_: 3352, 2926, 1653, 1456, 1113, 1018, 669 cm^−1^. ^1^H (600 MHz) and ^13^C (150 MHz) NMR spectral data, see Table 1. HRESIMS: *m*/*z* 310.1438 [M + H]^+^ (calcd for C_19_H_20_NO_3_^+^, 310.1438).

(+)-Tersone A (**1b**): white powders; ${\left[\alpha \right]}_{D}^{25}$ +44.9 (*c* 0.04, MeOH). CD (0.2 mg/mL, MeOH): 216 (+9.69), 245 (+4.57) nm. UV, IR, HRESIMS, ^1^H and ^13^C NMR data were the same as **1a**.

(−)-Tersone B (**2a**): white powders; ${\left[\alpha \right]}_{D}^{25}$ −58.6 (*c* 0.03, MeOH). CD (0.2 mg/mL, MeOH): 210 (−7.29), 224 (+3.89), 240 (−11.91) nm. UV (MeOH) *λ*_max_ (log *ε*): 246 (4.18) nm. IR *ν*_max_: 3368, 1647, 1429, 1111, 1016, 696 cm^−1^. ^1^H (600 MHz) and ^13^C (150 MHz) NMR spectral data, see Table 2. HRESIMS: *m*/*z* 310.1446 [M + H]^+^ (calcd for C_19_H_20_NO_3_^+^, 310.1438).

(+)-Tersone B (**2b**): white powders; ${\left[\alpha \right]}_{D}^{25}$ +56.7 (*c* 0.04, MeOH). CD (0.2 mg/mL, MeOH): 210 (+7.22), 224 (−4.69), 240 (+13.67) nm. UV, IR, HRESIMS, ^1^H and ^13^C NMR data were the same as **2a**.

(−)-Tersone C (**3a**): Mp 115–116 °C; white cubic crystals; ${\left[\alpha \right]}_{D}^{25}$ –50.9 (*c* 0.06, MeOH). CD (0.30 mg/mL, MeOH): 210 (–5.56), 224 (+3.99), 241 (−10.11) nm. UV (MeOH) *λ*_max_ (log *ε*): 246 (4.12) nm. IR *ν*_max_: 3422, 2926, 1645, 1429, 1215, 1018, 698 cm^−1^. ^1^H (600 MHz) and ^13^C (150 MHz) NMR spectral data, see Table 3. HRESIMS: *m*/*z* 310.1435 [M + H]^+^ (calcd for C_19_H_20_NO_3_^+^, 310.1438).

(+)-Tersone C (**3b**): Mp 112–113 °C; white cubic crystals; ${\left[\alpha \right]}_{D}^{25}$ +48.6 (*c* 0.07, MeOH). CD (0.26 mg/mL, MeOH): 210 (+7.58), 224 (−5.20), 241 (14.09) nm. UV, IR, HRESIMS, ^1^H and ^13^C NMR data were the same as **3a**.

(+)-Tersone D (**4a**): white powders; ${\left[\alpha \right]}_{D}^{25}$ +19.6 (*c* 0.05, MeOH). CD (0.18 mg/mL, MeOH): 208 (+3.18), 231 (+3.49), 259 (−1.57) nm. UV (MeOH) *λ*_max_ (log *ε*): 244 (4.13), 339 (3.58) nm. IR *ν*_max_: 3393, 2926, 1636, 1456, 1213, 1028, 761, 696 cm^−1^. ^1^H (600 MHz) and ^13^C (150 MHz) NMR spectral data, see Table 4. HRESIMS: *m*/*z* 294.1496 [M + H]^+^ (calcd for C_19_H_20_NO_2_^+^, 294.1489).

(−)-Tersone D (**4b**): white powders; ${\left[\alpha \right]}_{D}^{25}$ −22.6 (*c* 0.03, MeOH). CD (0.23 mg/mL, MeOH): 210 (−3.96), 233 (-3.96), 257 (+1.90) nm. UV, IR, HRESIMS, ^1^H and ^13^C NMR data were the same as **4a**.

(−)-Tersone E (**5a**): yellow powders; ${\left[\alpha \right]}_{D}^{25}$ −98.7 (*c* 0.08, MeOH). CD (0.16 mg/mL, MeOH): 211 (-6.55), 224 (+4.90), 240 (−13.35) nm. UV (MeOH) *λ*_max_ (log *ε*): 246 (4.23) nm. IR *ν*_max_: 2965, 1651, 1429, 1213, 1026, 825, 696 cm^−1^. ^1^H (600 MHz) and ^13^C (150 MHz) NMR spectral data, see Table 3. HRESIMS: *m*/*z* 294.1497 [M + H]^+^ (calcd for C_19_H_20_NO_2_^+^, 294.1489).

(+)-Tersone E (**5b**): yellow powders; ${\left[\alpha \right]}_{D}^{25}$ +99.1 (*c* 0.08, MeOH). CD (0.2 mg/mL, MeOH): 210 (+7.93), 224 (-5.84), 241 (+15.22) nm. UV, IR, HRESIMS, ^1^H and ^13^C NMR data were the same as **5a**.

Tersone F (**6**): white powders; ${\left[\alpha \right]}_{D}^{25}$ +208.0 (*c* 0.06, MeOH). CD (0.23 mg/mL, MeOH): 216 (+5.09), 235 (+4.81), 317 (+2.03) nm. UV (MeOH) *λ*_max_ (log *ε*): 241 (3.83) nm. IR *ν*_max_: 3446, 2922, 1697, 1635, 1020, 710 cm^−1^. ^1^H (600 MHz) and ^13^C (150 MHz) NMR spectral data, see Table 3. HRESIMS: *m*/*z* 344.1494 [M + H]^+^ (calcd for C_19_H_22_NO_5_^+^, 344.1492).

Tersone G (**7**): yellow powders; UV (MeOH) *λ*_max_ (log *ε*): 203 (3.56), 240 (3.69) nm. IR *ν*_max_: 3294, 1653, 1599, 1456, 1232, 694 cm^−1^. ^1^H (600 MHz) and ^13^C (150 MHz) NMR spectral data, see Table 4. HRESIMS: *m*/*z* 226.0867 [M + H]^+^ (calcd for C_14_H_12_NO_2_^+^, 226.0863).

### 3.4. X-ray Crytallographic Data of Compounds ***1a*** and ***3***

(−)-Tersone A (**1a**) and (±)-tersone C (**3**) were both crystallized from a mixture of methanol/water solvent system at room temperature. The single-crystal X-ray diffraction data were collected on Agilent Xcalibur Nova single-crystal diffractometer using CuK*α* radiation at 99.9 and 103K for **1a** and **3**, respectively. The crystal structure was refined by full-matrix least-squares calculation (for details see X-ray Crystallographic Analysis, Table S1, and Table S2 in the Supporting Information). Crystallographic data for (−)-Tersone A (**1a**) and (±)-tersone C (**3**) have been deposited at the Cambridge Crystallographic Data Center with the deposition number of CDCC 1910409 for **1a** and CDCC 1910462 for **3**, respectively. The copies of these data can be obtained free of charge via www.ccdc.cam.au.ck/conts/retrieving.html.

### 3.5. Antibacterial Assay

Compounds **1**–**9** were dissolved in DMSO at a final concentration of 10 mg/mL. The antibacterial activity evaluation was carried out in triplicate according to the standard microdilution method by using the vancomycin as the positive control [21]. The compounds were tested at concentrations ranging from 1.83 to 500 μg/mL. The test strains were *S. aureus* (CMCC 26003) and *E. coli* (ATCC 8739).

### 3.6. Cytotoxicity Assay

Compounds **1**–**9** were evaluated for their cytotoxic activity against SF-268, MCF-7, HepG-2, A549 cell lines by using the SRB method [22]. The cells (180 μL) with a density of 3 × 10^4^ cells/mL of media on 96-well plate were put under 37 °C at 5% CO_2_ condition and incubated for 24 h. Then, 20 μL of various concentrations of compounds were added and further incubated for 72 h. After that, the cell monolayers were fixed by 50% (*wt*/*v*) trichloroacetic acid (50 μL) and stained for 30 min by 0.4% (*wt*/*v*) SRB, which was dissolved in 1% acetic acid. The unbound dye was removed by washing repeatedly with 1% acetic acid, and then dissolved into the protein-bound dye in 10 mM Tris base solution (200 μL) for OD determination at 570 nm using a microplate reader. Cisplatin was used as a positive control possessing potent cytotoxic activity. All data were obtained in triplicate, and the IC_50_ values were calculated by the SigmaPlot 10.0 software (Systat Software Inc., San Jose, CA, America) with the use of a non-linear curve-fitting method. The SF-268, MCF-7, HepG-2, A549 cell lines were provided by the Chinese Academy of Sciences Cell Bank.

## Figures and Tables

**Table 1:** ^1^H (600 MHz) and ^13^C (150 MHz) NMR data for compounds **1** and **2** in CD_3_OD.

No.	1		No.	2	
	*δ*_H_ (*J* in Hz)	*δ*_C_		*δ*_H_ (*J* in Hz)	*δ*_C_
2		105.1, C	2		104.7, C
3	3.22 (d, 1.8)	57.9, CH	3	3.28 (br s)	58.0, CH
3a		114.8, C	3a		114.8, C
4		163.2, C	4		163.3, C
6	7.34 (s)	134.1, CH	6	7.44 (s)	135.1, CH
7		113.3, C	7		113.0, C
7a		166.7, C	7a		166.5, C
8		125.6, C	8		134.5, C
9	7.32 (d, 8.7)	130.0, CH	9	7.50 (d, 7.7)	128.7, CH
10	6.79 (d, 8.6)	116.3, CH	10	7.37 (t, 7.7)	129.5, CH
11		158.2, C	11	7.29 (t, 7.7)	128.5, CH
12	6.79 (d, 8.6)	116.3, CH	12	7.37 (t, 7.7)	129.5, CH
13	7.32 (d, 8.7)	130.0, CH	13	7.50 (d, 7.7)	128.7, CH
1′	5.40 (s)	127.4, CH	1′	5.65 (s)	126.3, CH
2′		151.7, C	2′		155.4, C
3′	2.80 (q, 7.4)	50.6, CH	3′	3.00 (q, 7.4)	47.0, CH
4′	1.27 (d, 7.2)	20.5, CH_3_	4′	1.29 (d, 6.9)	20.8, CH_3_
5′	1.74 (s)	14.8, CH_3_	5′*α* 5′*β*	4.10 (d, 15.3) 4.18 (d, 15.3)	60.2, CH_2_
6′	1.63 (s)	26.5, CH_3_	6′	1.68 (s)	26.3, CH_3_

**Table 2:** ^1^H (600 MHz) and ^13^C (150 MHz) NMR data for compounds **3**–**4** in CD_3_OD.

No.	3		No.	4	
	*δ*_H_ (*J* in Hz)	*δ*_C_		*δ*_H_ (*J* in Hz)	*δ*_C_
2		108.2, C	2	5.23 (s)	87.3, CH
3	3.49 (d, 1.8)	53.2, CH	3		129.7, C
3a		115.3, C	4	6.50 (s)	115.1, CH
4		163.2, C	4a		108.3, C
6	7.44 (s)	135.0, CH	5		162.5, C
7		113.1, C	7	7.21 (s)	134.6, CH
7a		167.1, C	8		116.6, C
8		134.6, C	8a		160.8, C
9	7.54 (d, 7.1)	128.9, CH	9		134.9, C
10	7.37 (t, 7.7)	129.5, CH	10	7.36 (overlapped)	129.3, CH
11	7.29 (t, 7.4)	128.4, CH	11	7.36 (overlapped)	130.0, CH
12	7.37 (t, 7.7)	129.5, CH	12	7.30 (t, 7.5)	128.5, CH
13	7.54 (d, 7.1)	128.9, CH	13	7.36 (overlapped)	130.0, CH
1′	5.39 (s)	123.7, CH	14	7.36 (overlapped)	129.3, CH
2′		154.4, C	1′	1.64 (overlapped)	11.7, CH_3_
3′	2.83 (q, 7.3)	50.5, CH	2′		133.9, C
4′	1.29 (d, 7.2)	20.4, CH_3_	3′	5.61 (q, 6.2)	126.2, CH
5′	1.78 (s)	15.1, CH_3_	4′	1.64 (overlapped)	13.3, CH_3_
6′*α* 6′*β*	3.67 (d, 12.2) 3.89 (d, 12.2)	66.7, CH_2_	5′	1.72 (s)	19.6, CH_3_

**Table 3:** ^1^H (600 MHz) and ^13^C (150 MHz) NMR data for compounds **5** and **6** in CD_3_OD.

No.	5		No.	6	
	*δ*_H_ (*J* in Hz)	*δ*_C_		*δ*_H_ (*J* in Hz)	*δ*_C_
2		105.3, C	1		211.7, C
3	3.23 (d, 1.8)	57.8, CH	2		134.3, C
3a		114.9, C	3		174.6, C
4		166.6, C	4	2.14 (d, 18.1) 3.68 (d, 18.1)	43.8, CH_2_
5		113.0, C	5		57.4, C
6	7.43 (s)	135.0, CH	6		175.0, C
7a		163.3, C	7		76.1, C
8		134.5, C	8		209.0, C
9	7.49 (d, 7.0)	128.7, CH	9	4.13 (d, 2.9)	68.7, CH
10	7.36 (t, 7.7)	129.5, CH	10	5.14 (d, 2.9)	72.4, CH
11	7.29 (t, 7.4)	128.4, CH	11		142.3, C
12	7.36 (t, 7.7)	129.5, CH	12	7.39 (d, 6.9)	127.1, CH
13	7.49 (d, 7.0)	128.7, CH	13	7.37 (t, 6.9)	129.6, CH
1′	2.80 (q, 7.2)	50.5, CH	14	7.28 (t, 6.9)	128.7, CH
2′		151.8, C	15	7.37 (t, 6.9)	129.6, CH
3′	5.40 (s)	127.3, CH	16	7.39 (d, 6.9)	127.1, CH
4′	1.63 (s)	26.4, CH_3_	17	1.57 (s)	7.8, CH_3_
5′	1.74 (s)	14.8, CH_3_	18	2.04 (s)	17.2, CH_3_
6′	1.27 (d, 7.2)	20.5, CH_3_	19	1.25 (s)	19.0, CH_3_

**Table 4:** ^1^H (600 MHz) and ^13^C (150 MHz) NMR data for compound **7** in CD_3_OD.

No.	7		No.	7	
	*δ*_H_ (*J* in Hz)	*δ*_C_		*δ*_H_ (*J* in Hz)	*δ*_C_
2		156.4, C	8		133.9, C
3	6.66 (s)	103.3, CH	9	7.70 (d, 7.5)	128.6, CH
3a		118.4, C	10	7.46 (t, 7.5)	129.9, CH
4		159.6, C	11	7.38 (t, 7.5)	128.9, CH
6	7.43 (s)	128.6, CH	12	7.46 (t, 7.5)	129.9, CH
7		113.2, C	13	7.70 (d, 7.5)	128.6, CH
7a		161.6, C	1′	2.48 (s)	13.6, CH_3_

**Table 5:** In vitro antibacterial evaluation (MIC) of **1**–**9**.

Compounds	MIC (μg/mL)		Compounds	MIC (μg/mL)	
	*S. aureus* (ATCC 29213)	*E. coli* (ATCC 8739)		*S. aureus* (ATCC 29213)	*E. coli* (ATCC 8739)
**1–3**	>500	>500	**6–7**	>500	>500
**(+)-4a**	125	>500	**(−)-8a**	62.5	>500
**(−)-4b**	125	>500	**(+)-8b**	31.5	>500
**(−)-5a**	62.5	>500	**9**	250	>500
**(+)-5b**	31.2	>500	Van	1.8	125

## Supplementary Materials

The following are available online at https://www.mdpi.com/1660-3397/17/7/394/s1. Figures S1–S88: 1D, 2D NMR, HRESIMS, CD, UV and IR spectra of compounds **1**–**7**; Figures S89–S94: 1D NMR spectra of compounds **8**–**9**.

## References

[1] Li X.B., Li L., Zhu R.X., Li W., Chang W.Q., Zhang L.L., Wang X.N., Zhao Z.T., Lou H.X. (2015). Tetramic acids and pyridone alkaloids from the endolichenic fungus *Tolypocladium cylindrosporum*. J. Nat. Prod..

[2] Shang Z., Li L., Esposito B.P., Salim A.A., Khalil Z.G., Quezada M., Bernhardt P.V., Capon R.J. (2015). New PKS-NRPS tetramic acids and pyridinone from an Australian marine-derived fungus, *Chaunopycnis* sp.. Org. Biomol. Chem..

[3] Sakemi S., Bordner J., Decosta D.L., Dekker K.A., Hirai H., Inagaki T., Kim Y.J., Kojima N., Sims J.C., Sugie Y. (2002). CJ-15, 696 and its analogs, new furopyridine antibiotics from the fungus *Cladobotryum varium*: Fermentation, isolation, structural elucidation, biotransformation and antibacterial activities. J. Antibiot..

[4] Breinholt J., Jensen H.C., Kjær A., Olsen C.E., Rassing B.R., Rosendahl C.N., Søtofte I. (1998). Cladobotryal: A fungal metabolite with a novel ring system. Acta Chem. Scand..

[5] Xu Y., Wang L., Zhu G., Zuo M., Gong Q., He W., Li M., Yuan C., Hao X., Zhu W. (2019). New phenylpyridone derivatives from the *Penicillium sumatrense* GZWMJZ-313, a fungal endophyte of Garcinia multiflora. Chin. Chem. Lett..

[6] Fukuda T., Yamaguchi Y., Masuma R., Tomoda H., Omura S. (2005). Citridones, new potentiators of antifungal miconazole activity, produced by *Penicillium* sp. FKI-1938. I. taxonomy, fermentation, isolation and biological properties. J. Antibiot..

[7] Fukuda T., Sakabe Y., Tomoda H., Omura S. (2006). Fungal citridone D having a novel phenylfuropyridine skeleton. Chem. Pharm. Bull..

[8] Fotiadou A.D., Zografos A.L. (2011). Accessing the structural diversity of pyridone alkaloids: Concise total synthesis of rac-citridone A. Org. Lett..

[9] Snider B.B., Che Q. (2004). Synthesis of cladobotryal, CJ16, 169, and CJ16, 170. Org. Lett..

[10] Clive D.L., Huang X. (2004). Model studies and first synthesis of the antifungal and antibacterial agent cladobotryal. J. Org. Chem..

[11] Clive D.L., Huang X. (2002). Studies related to furopyridinone antibiotics. Synthesis of 2-epi-CJ-16,170. Tetrahedron.

[12] Moore M.C., Cox R.J., Duffin G.R., O’Hagan D. (1998). Synthesis and evaluation of a putative acyl tetramic acid intermediate in tenellin biosynthesis in *Beauveria bassiana*. A new role for tyrosine. Tetrahedron.

[13] Tomoda H. (2016). New approaches to drug discovery for combating MRSA. Chem. Pharm. Bull..

[14] Miyagawa T., Nagai K., Yamada A., Sugihara Y., Fukuda T., Fukuda T., Uchida R., Tomoda H., O̅mura S., Nagamitsu T. (2011). Total synthesis of citridone A. Org. Lett..

[15] Fukuda T., Shimoyama K., Nagamitsu T., Tomoda H. (2014). Synthesis and biological activity of Citridone A and its derivatives. J. Antibiot..

[16] Liu H., Tan H., Chen Y., Guo X., Wang W., Guo H., Liu Z., Zhang W. (2019). Cytorhizins A−D, four highly structure-combined benzophenones from the endophytic fungus *Cytospora rhizophorae*. Org. Lett..

[17] Liu H., Tan H., Wang W., Zhang W., Chen Y., Li S., Liu Z., Li H., Zhang W. (2019). Cytorhizophins A and B, benzophenone-hemiterpene adducts from the endophytic fungus *Cytospora rhizophorae*. Org. Chem. Front..

[18] Xu J., Tan H., Chen Y., Li S., Huang Z., Guo H., Li H., Gao X., Liu H., Zhang W. (2018). Lithocarpins A–D: Four tenellone-macrolide conjugated [4 + 2] hetero-adducts from the deep-sea derived fungus *Phomopsis lithocarpus* FS508. Org. Chem. Front..

[19] Chen S., Li H., Chen Y., Li S., Xu J., Guo H., Liu Z., Zhu S., Liu H., Zhang W. (2019). Three new diterpenes and two new sesquiterpenoids from the endophytic fungus *Trichoderma koningiopsis* A729. Bioorg. Chem..

[20] Fukuda T., Tomoda H., Omura S. (2005). Citridones, new potentiators of antifungal miconazole activity, produced by *Penicillium* sp. FKI-1938: II. structure elucidation. J. Antibiot..

[21] Drummond A.J., Waigh R.D., Pandalai S.G. (2000). Recent Research Developments in Phytochemistry.

[22] Skehan P., Storeng R., Scudiero D., Monks A., McMahon J., Vistica D., Warren J.T., Bokesch H., Kenney S., Boyd M.R. (1990). New colorimetric cytotoxicity assay for anticancer-drug screening. J. Natl. Cancer. Inst..

