# Supplementary material for: Tersone A-G, New Pyridone Alkaloids from the Deep-Sea Fungus Phomopsis tersa
Source: Mar Drugs. 2019 Jul 3;17(7):394. doi: 10.3390/md17070394 (PMC6669727; doi:10.3390/md17070394)
Supplement: Supplementary file 1 [file marinedrugs-17-00394-s001.zip › Supplementary Files/Supporting Information.pdf]

Supporting Information

Tersone A-G, new pyridone alkaloids from the deep-sea fungus *Phomopsis tersa*

Shan-Chong Chen[1,2], Zhao-Ming Liu[1], Hai-Bo Tan[3], Yu-Chan Chen[1], Sai-Ni Li[1], Hao-Hua Li[1], Heng Guo[1], Shuang Zhu[2], Hong-Xin Liu[1,*] and Wei-Min Zhang[1,*]

[1]State Key Laboratory of Applied Microbiology Southern China, Guangdong Provincial Key Laboratory of Microbial Culture Collection and Application, Guangdong Open Laboratory of Applied Microbiology, Guangdong Institute of Microbiology, Guangdong Academy of Sciences, Guangzhou 510070, China

[2]School of Biosciences and Biopharmaceutics, Guangdong Pharmaceutical University, Guangzhou 510006, China

[3]Program for Natural Products Chemical Biology, Key Laboratory of Plant Resources Conservation and Sustainable Utilization, Guangdong Provincial Key Laboratory of Applied Botany, South China Botanical Garden, Chinese Academy of Sciences, Guangzhou 510650, China

Contents

1. Experimental Section

1.1 X-ray crystallographic analysis of compounds **1a** and **3**.

1.2 Computational details

2. NMR, HRESIMS, CD, UV and IR spectra of compounds 1-9

Figure S1. ^{1}H NMR spectrum (600 MHz, CD_3OD) of **1a**

Figure S2. ^{13}C NMR spectrum (150 MHz, CD_3OD) of **1a**

Figure S3. ^{1}H-^{1}H COSY spectrum of **1a** in CD_3OD

Figure S4. HSQC spectrum of **1a** in CD_3OD

Figure S5. HMBC spectrum of **1a** in CD_3OD

Figure S6. NOESY spectrum of **1a** in CD_3OD

Figure S7. HRESIMS spectrum of **1a**

Figure S8. CD spectrum of **1a**

Figure S9. UV spectrum of **1a**

Figure S10. IR spectrum of **1a**

Figure S11. ^{1}H NMR spectrum (600 MHz, CD_3OD) of **1b**

Figure S12. ^{13}C NMR spectrum (150 MHz, CD_3OD) of **1b**

Figure S13. HRESIMS spectrum of **1b**

Figure S14. CD spectrum of **1b**

Figure S15. ^{1}H NMR spectrum (600 MHz, CD_3OD) of **2a**

Figure S16. ^{13}C NMR spectrum (150 MHz, CD_3OD) of **2a**

Figure S17. ^{1}H-^{1}H COSY spectrum of **2a** in CD_3OD

Figure S18. HSQC spectrum of **2a** in CD_3OD

Figure S19. HMBC spectrum of **2a** in CD_3OD

Figure S20. NOESY spectrum of **2a** in CD_3OD

Figure S21. HRESIMS spectrum of **2a**

Figure S22. CD spectrum of **2a**

Figure S23. UV spectrum of **2a**

Figure S24. IR spectrum of **2a**

Figure S25. ^{1}H NMR spectrum (600 MHz, CD_3OD) of **2b**

Figure S26. ^{13}C NMR spectrum (150 MHz, CD_3OD) of **2b**

Figure S27. HRESIMS spectrum of **2b**

Figure S28. CD spectrum of **2b**

Figure S29. ^{1}H NMR spectrum (600 MHz, CD_3OD) of **3a**

Figure S30. ^{13}C NMR spectrum (150 MHz, CD_3OD) of **3a**

Figure S31. ^{1}H-^{1}H COSY spectrum of **3a** in CD_3OD

Figure S32. HSQC spectrum of **3a** in CD_3OD

Figure S33. HMBC spectrum of **3a** in CD_3OD

Figure S34. NOESY spectrum of **3a** in CD_3OD

Figure S35. HRESIMS spectrum of **3a**

Figure S36. CD spectrum of **3a**

Figure S37. UV spectrum of **3a**

Figure S38. IR spectrum of **3a**

Figure S39. ^{1}H NMR spectrum (600 MHz, CD_3OD) of **3b**

Figure S40. ^{13}C NMR spectrum (150 MHz, CD_3OD) of **3b**

Figure S41. HRESIMS spectrum of **3b**

Figure S42. CD spectrum of **3b**

Figure S43. ^{1}H NMR spectrum (600 MHz, CD_3OD) of **4a**

Figure S44. ^{13}C NMR spectrum (150 MHz, CD_3OD) of **4a**

Figure S45. ^{1}H-^{1}H COSY spectrum of **4a** in CD_3OD

Figure S46. HSQC spectrum of **4a** in CD_3OD

Figure S47. HMBC spectrum of **4a** in CD_3OD

Figure S48. NOESY spectrum of **4a** in CD_3OD

Figure S49. HRESIMS spectrum of **4a**

Figure S50. CD spectrum of **4a**

Figure S51. UV spectrum of **4a**

Figure S52. IR spectrum of **4a**

Figure S53. ^{1}H NMR spectrum (600 MHz, CD_3OD) of **4b**

Figure S54. ^{13}C NMR spectrum (150 MHz, CD_3OD) of **4b**

Figure S55. HRESIMS spectrum of **4b**

Figure S56. CD spectrum of **4b**

Figure S57. ^{1}H NMR spectrum (600 MHz, CD_3OD) of **5a**

Figure S58. ^{13}C NMR spectrum (150 MHz, CD_3OD) of **5a**

Figure S59. ^{1}H-^{1}H COSY spectrum of **5a** in CD_3OD

Figure S60. HSQC spectrum of **5a** in CD_3OD

Figure S61. HMBC spectrum of **5a** in CD_3OD

Figure S62. NOESY spectrum of **5a** in CD_3OD

Figure S63. HRESIMS spectrum of **5a**

Figure S64. CD spectrum of **5a**

Figure S65. UV spectrum of **5a**

Figure S66. IR spectrum of **5a**

Figure S67. ^{1}H NMR spectrum (600 MHz, CD_3OD) of **5b**

Figure S68. ^{13}C NMR spectrum (150 MHz, CD_3OD) of **5b**

Figure S69. HRESIMS spectrum of **5b**

Figure S70. CD spectrum of **5b**

Figure S71. ^{1}H NMR spectrum (600 MHz, CD_3OD) of **6**

Figure S72. ^{13}C NMR spectrum (150 MHz, CD_3OD) of **6**

Figure S73. ^{1}H-^{1}H COSY spectrum of **6** in CD_3OD

Figure S74. HSQC spectrum of **6** in CD_3OD

Figure S75. HMBC spectrum of **6** in CD_3OD

Figure S76. NOESY spectrum of **6** in CD_3OD

Figure S77. HRESIMS spectrum of **6**

Figure S78. CD spectrum of **6**

Figure S79. UV spectrum of **6**

Figure S80. IR spectrum of **6**

Figure S81. ^{1}H NMR spectrum (600 MHz, CD_3OD) of **7**

Figure S82. ^{13}C NMR spectrum (150 MHz, CD_3OD) of **7**

Figure S83. ^{1}H-^{1}H COSY spectrum of **7** in CD_3OD

Figure S84. HSQC spectrum of **7** in CD_3OD

Figure S85. HMBC spectrum of **7** in CD_3OD

Figure S86. HRESIMS spectrum of **7**

Figure S87. UV spectrum of **7**

Figure S88. IR spectrum of **7**

Figure S89. ^1H NMR spectrum (600 MHz, CD_3OD) of **8a**

Figure S90. ^{13}C NMR spectrum (150 MHz, CD_3OD) of **8a**

Figure S91. ^1H NMR spectrum (600 MHz, CD_3OD) of **8b**

Figure S92. ^{13}C NMR spectrum (150 MHz, CD_3OD) of **8b**

Figure S93. ^1H NMR spectrum (600 MHz, CD_3OD) of **9**

Figure S94. ^{13}C NMR spectrum (150 MHz, CD_3OD) of **9**

1. Experimental Section

1.1 X-ray crystallographic analysis of compounds **1a** and **3**.

Table S1. X-ray crystallographic data for (−)-tersone A (**1a**).

Empirical formula	$C_{19}H_{19}NO_3$
Formula weight	309.34
Temperature	99.9(8) K
Wavelength	1.54184 Å
Crystal system	monoclinic
Space group	12
Unit cell dimensions	a = 15.4694(2) Å α = 90 °.
	b = 11.28363(15) Å β = 101.9564(15) °.
	c = 21.3051(3) Å γ = 90 °.
Volume	3638.15(9) $Å^3$
Z	8
Density (calculated)	1.126 Mg/m^3
Absorption coefficient	0.617 mm^{-1}
F(000)	1304.0
Crystal size	0.08 x 0.06 x 0.05 mm^3
Theta range for data collection	8.484 to 148.346 °.
Index ranges	-18<=h<=19, -13<=k<=13, -26<=l<=26
Reflections collected	18985
Independent reflections	7142 [R(int) = 0.0489]
Completeness to theta = 66.97 °	99.94 %
Absorption correction	multi-scan
Refinement method	Full-matrix least-squares on F^2
Data / restraints / parameters	7142 / 1 / 423
Goodness-of-fit on F^2	1.062
Final R indices [I>2sigma(I)]	R1 = 0.0404, wR2 = 0.1055
R indices (all data)	R1 = 0.0483, wR2 = 0.1101
Absolute structure parameter	0.09(10)
Largest diff. peak and hole	0.23 and -0.19 e.$Å^{-3}$

Table S2. X-ray crystallographic data for (±)-tersone C (**3**).

Empirical formula	$C_{19}H_{19}NO_3$
Formula weight	309.35
Temperature	103(6) K
Wavelength	1.54184 Å
Crystal system	monoclinic
Space group	$P2_1/n$
Unit cell dimensions	a = 8.94020(10) Å α = 90 °.
	b = 17.2446(2) Å β = 98.8880(10) °.
	c = 10.33770(10) Å γ = 90 °.
Volume	1574.63(3) $Å^3$
Z	4
Density (calculated)	1.305 Mg/m^3
Absorption coefficient	0.713 mm^{-1}
F(000)	656.0
Crystal size	0.08 x 0.07 x 0.05 mm^3
Theta range for data collection	10.066 to 148.01 °.
Index ranges	-10<=h<=10, -15<=k<=21, -12<=l<=11
Reflections collected	8528
Independent reflections	3083 [R(int) = 0.0280]
Completeness to theta = 67.97 °	99.76 %
Absorption correction	multi-scan
Refinement method	Full-matrix least-squares on F^2
Data / restraints / parameters	3083 / 0 / 211
Goodness-of-fit on F^2	1.044
Final R indices [I>2sigma(I)]	R1 = 0.0368, wR2 = 0.0964
R indices (all data)	R1 = 0.0429, wR2 = 0.1002
Largest diff. peak and hole	0.20 and -0.23 e.$Å^{-3}$

1.2 Computational details

Methods. Merck molecular force field (MMFF) and DFT/TD-DFT calculations were carried out with the Spartan’14 software (Wavefunction Inc., Irvine, CA, USA) and the Gaussian 09 program, respectively [1]. Conformers within the 10 kcal mol^{-1} energy window were generated and optimized using DFT calculations at the b3lyp/6-31+g(d,p) level. Frequency calculations were performed at the same level to confirm that each optimized conformer was true minimum and to estimate their relative thermal free energy (ΔG) at 298.15 K. Conformers with the Boltzmann distribution over 5% were chosen for ECD calculations in methanol at the b3lyp/6-311+g(d,p) level. Solvent effects were taken into consideration using the self-consistent reaction field (SCRF) method with the polarizable continuum model (PCM) [2].The ECD spectrum was generated by the

SpecDis program [3] using a Gaussian band shape with 0.26 eV exponential half-width from dipole-length dipolar and rotational strengths.

Results.

Table S3. Energy analysis for the conformers of **4**.

compounds	conformation	G (Hartree)	G (Kcal/mol)	ΔG (Kcal/mol)	Boltzmann Dist (%)
**4**	a	-940.73426083	-590313.4768	0	74.82%
	b	-940.73323300	-590312.8319	0.644901673	25.18%

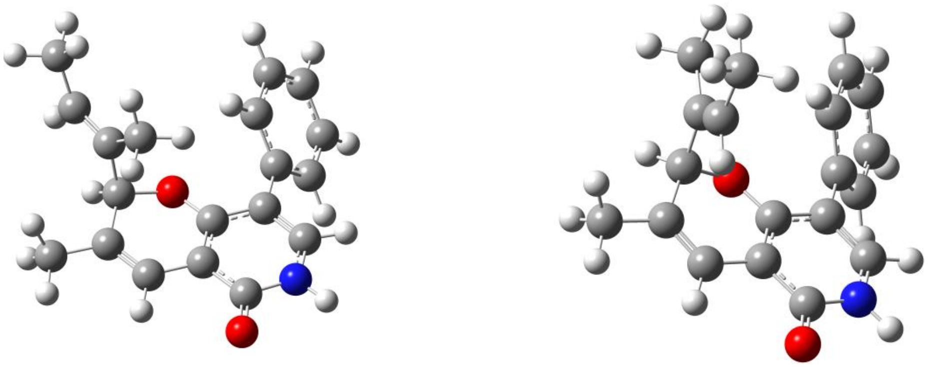

a b

Figure S1. B3LYP/6-31G(d) optimized low-energy conformers of **4**

References

1. Frisch, M.J.; Trucks, G.W.; Schlegel, H.B.; Scuseria, G.E.; Robb, M.A.; Cheeseman, J.R.; Scalmani, G.; Barone, V.; Mennucci, B.; Petersson, G.A.; Nakatsuji, H.; Caricato, M.; Li, X.; Hratchian, H.P.; Izmaylov, A.F.; Bloino, J.; Zheng, G.; Sonnenberg, J.L.; Hada, M.; Ehara, M.; Toyota, K.; Fukuda, R.; Hasegawa, J.; Ishida, M.; Nakajima, T.; Honda,Y.; Kitao, O.; Nakai, H.; Vreven, T.; Montgomery Jr., J.A.; Peralta, J.E.; Ogliaro, F.; Bearpark, M.; Heyd, J.J.; Brothers, E.; Kudin, K.N.; Staroverov, V.N.; Kobayashi, R.; Normand, J.; Raghavachari, K.; Rendell, A.; Burant, J.C.; Iyengar, S.S.; Tomasi, J.; Cossi, M.; Rega, N.; Millam, J.M.; Klene, M.; Knox, J.E.; Cross, J.B.; Bakken, V.; Adamo, C.; Jaramillo, J.; Gomperts, R.; Stratmann, R.E.; Yazyev, O.; Austin, A.J.; Cammi, R.; Pomelli, C.; Ochterski, J.W.; Martin, R.L.; Morokuma, K.; Zakrzewski, V.G.; Voth, G. A.; Salvador, P.; Dannenberg, J.J.; Dapprich, S.; Daniels, A.D.; Farkas, Ö.; Foresman, J.B.; Ortiz,J.V.; Cioslowski J.; Fox, D.J. Gaussian 09, revision D.01, Gaussian, Inc., Wallingford, CT, **2013**.
2. Wu, P.; Xue, J.; Yao, L.; Xu, L.; Li, H.; Wei, X. Bisacremines E-G, three polycyclic dimeric acremines produced by *Acremonium persicinum* SC0105. *Org. Lett.*, **2015**, *17*, 4922.
3. Bruhn, T.; Schauml öffel, A.; Hemberger, Y.; Bringmann, G. SpecDis: Quantifying the comparison of calculated and experimental electronic circular dichroism spectra. *Chirality*, **2013**, *25*, 243.

2. NMR, HRESIMS, CD, UV and IR spectrum of compounds 1-9

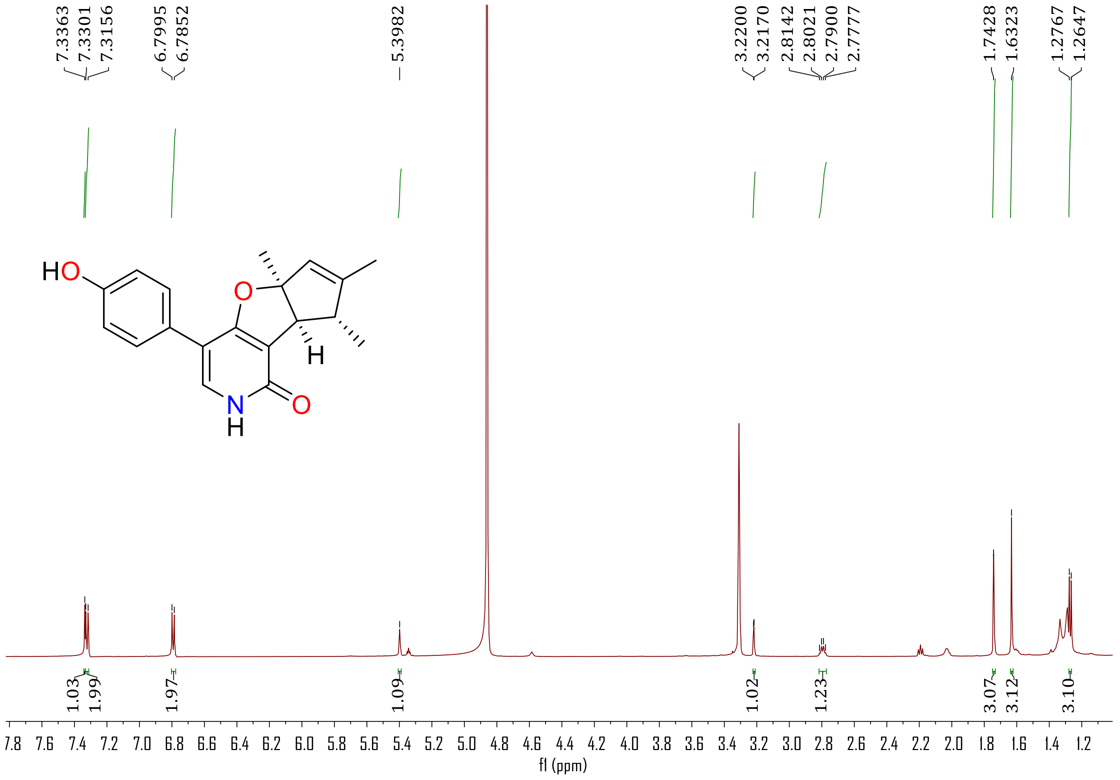

Figure S1. ^{1}H NMR spectrum (600 MHz, CD_3OD) of **1a**

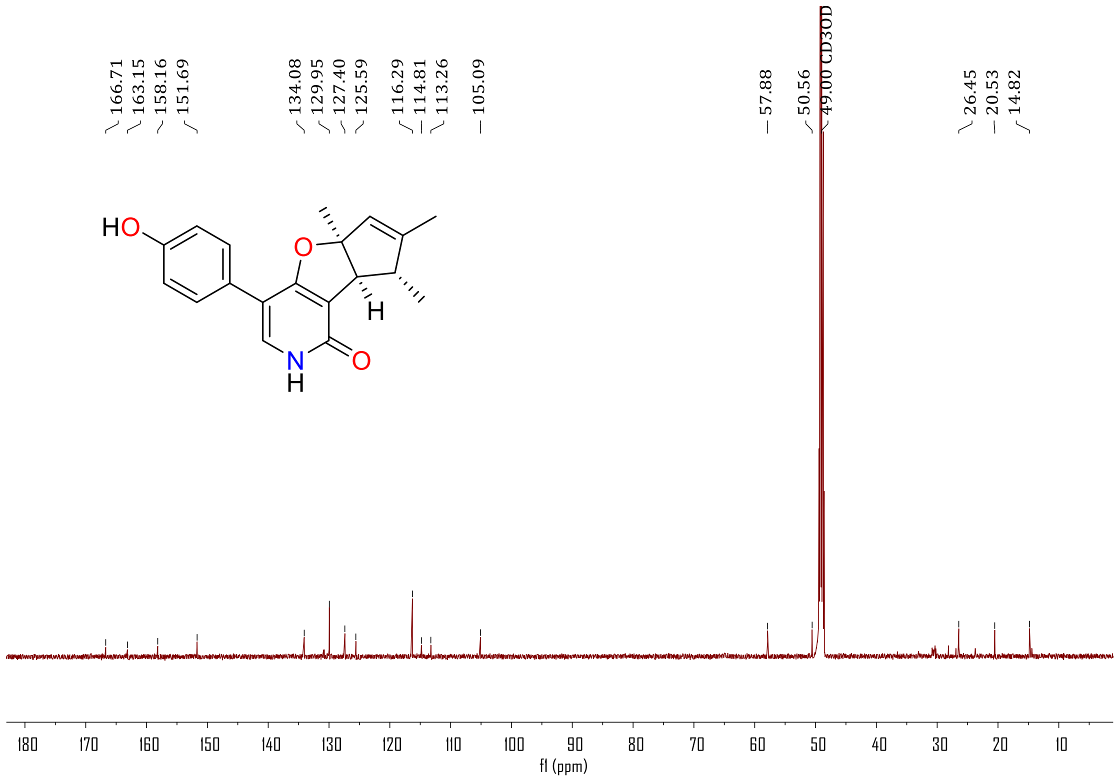

Figure S2. ^{13}C NMR spectrum (150 MHz, CD_3OD) of **1a**

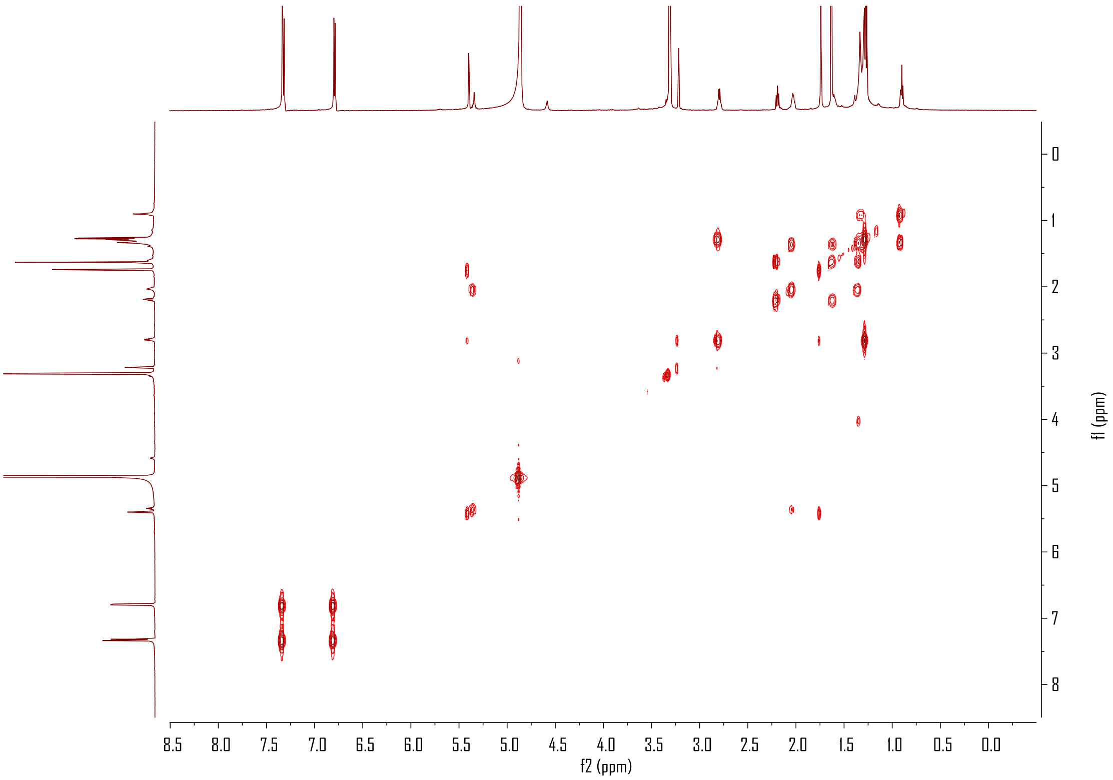

Figure S3. ^{1}H-^{1}H COSY spectrum of **1a** in CD_3OD

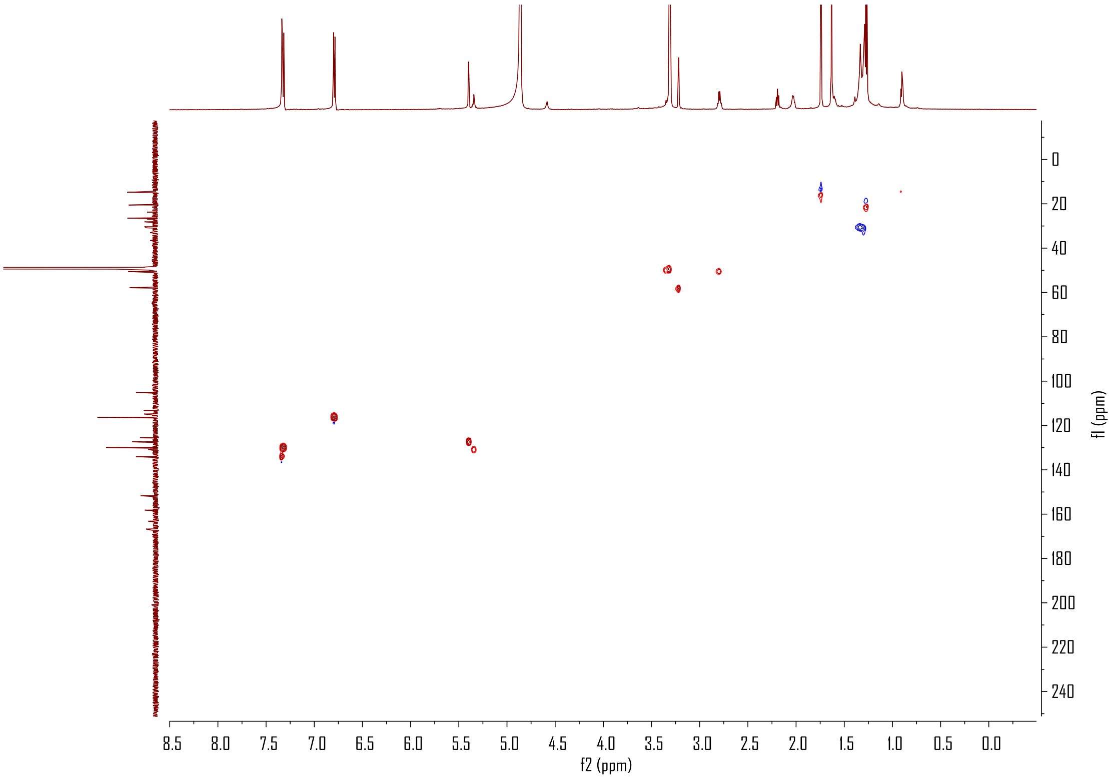

Figure S4. HSQC spectrum of **1a** in CD_3OD

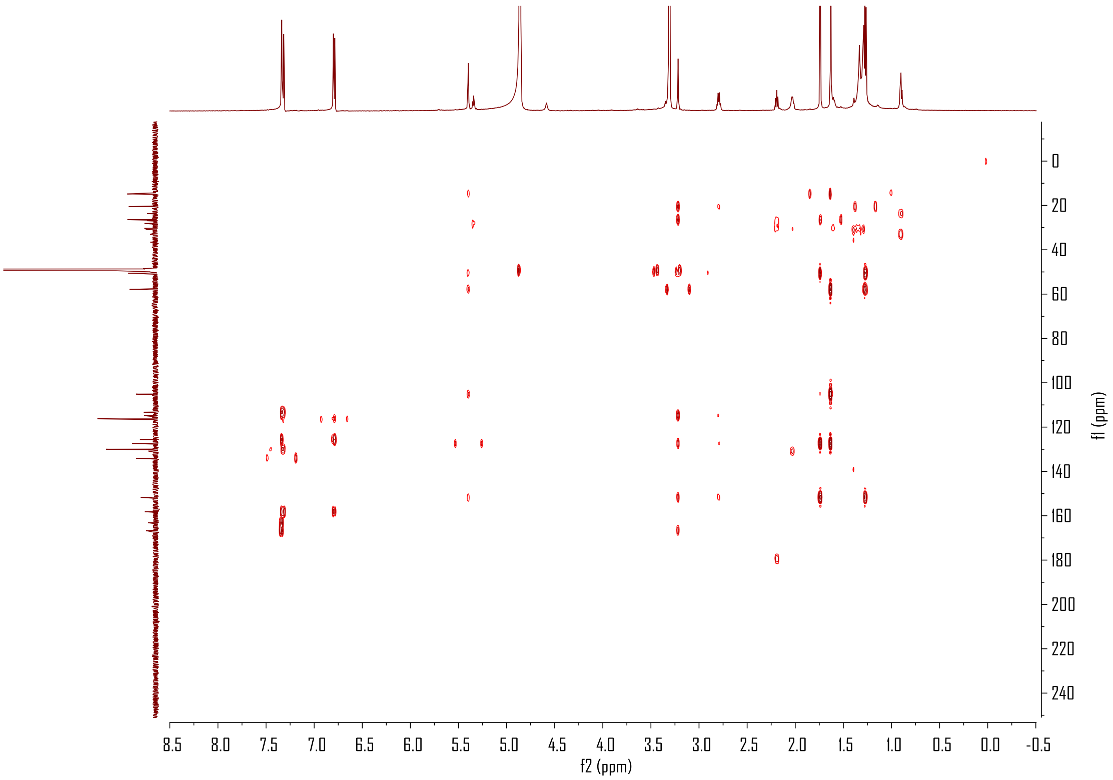

Figure S5. HMBC spectrum of **1a** in CD_3OD

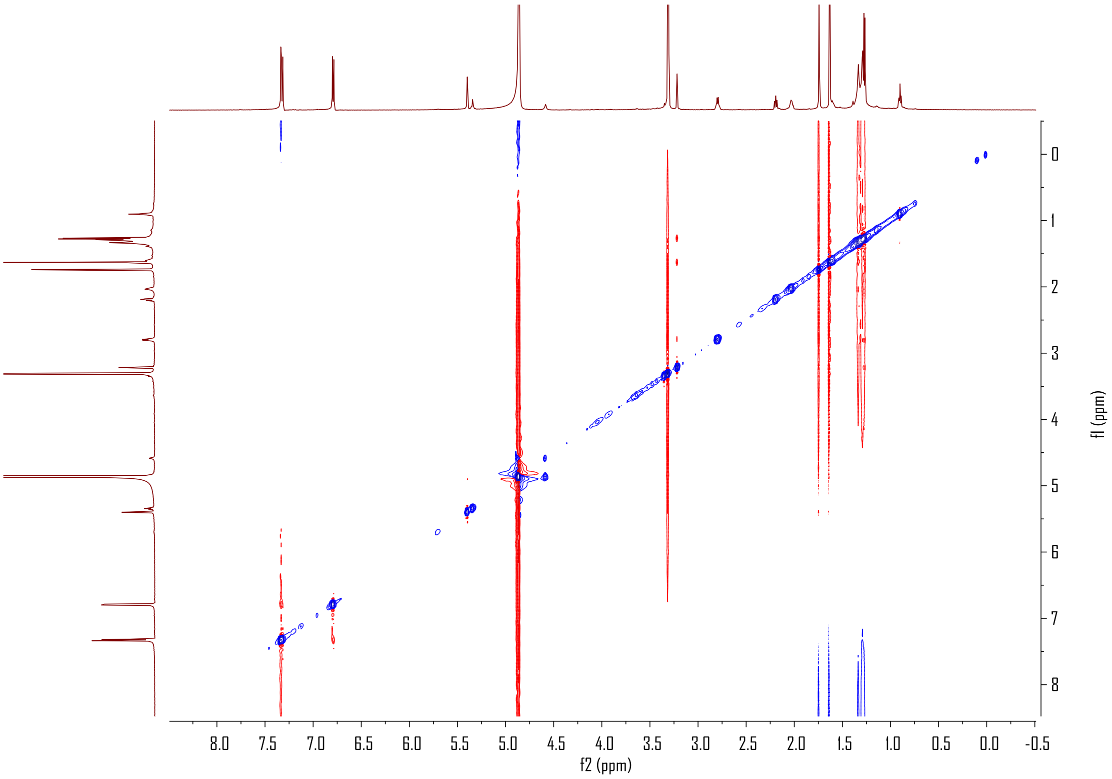

Figure S6. NOESY spectrum of **1a** in CD_3OD

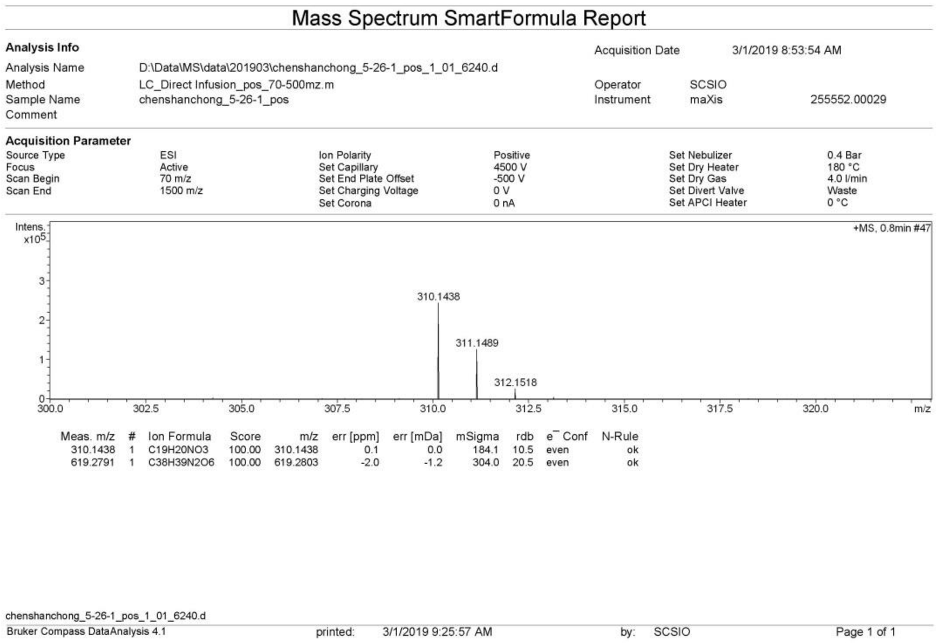

Mass Spectrum SmartFormula Report

Analysis Info

Analysis Name	D:\Data\MS\data\201903\chenshanchong_5-26-1_pos_1_01_6240.d	Acquisition Date	3/1/2019 8:53:54 AM
Method	LC_Direct Infusion_pos_70-500mz.m	Operator	SCSIO
Sample Name	chenshanchong_5-26-1_pos	Instrument	maXis 255552.00029
Comment			

Acquisition Parameter

Source Type	ESI	Ion Polarity	Positive	Set Nebulizer	0.4 Bar
Focus	Active	Set Capillary	4500 V	Set Dry Heater	180 °C
Scan Begin	70 m/z	Set End Plate Offset	-500 V	Set Dry Gas	4.0 l/min
Scan End	1500 m/z	Set Charging Voltage	0 V	Set Divert Valve	Waste
		Set Corona	0 nA	Set APCI Heater	0 °C

Meas. m/z	#	Ion Formula	Score	m/z	err [ppm]	err [mDa]	mSigma	rdb	e⁻ Conf	N-Rule
310.1438	1	C19H20NO3	100.00	310.1438	0.1	0.0	184.1	10.5	even	ok
619.2791	1	C38H39N2O6	100.00	619.2803	-2.0	-1.2	304.0	20.5	even	ok

chenshanchong_5-26-1_pos_1_01_6240.d
Bruker Compass DataAnalysis 4.1 printed: 3/1/2019 9:25:57 AM by: SCSIO Page 1 of 1

Figure S7. HRESIMS spectrum of **1a**

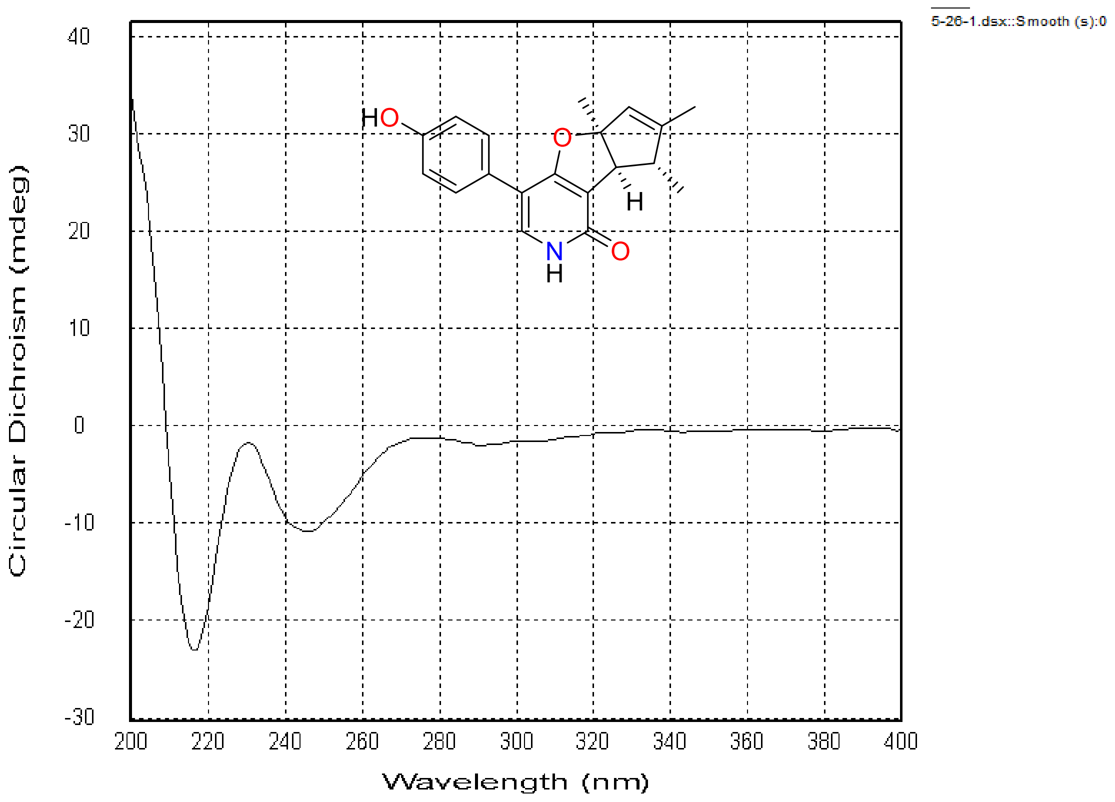

Figure S8. CD spectrum of **1a**

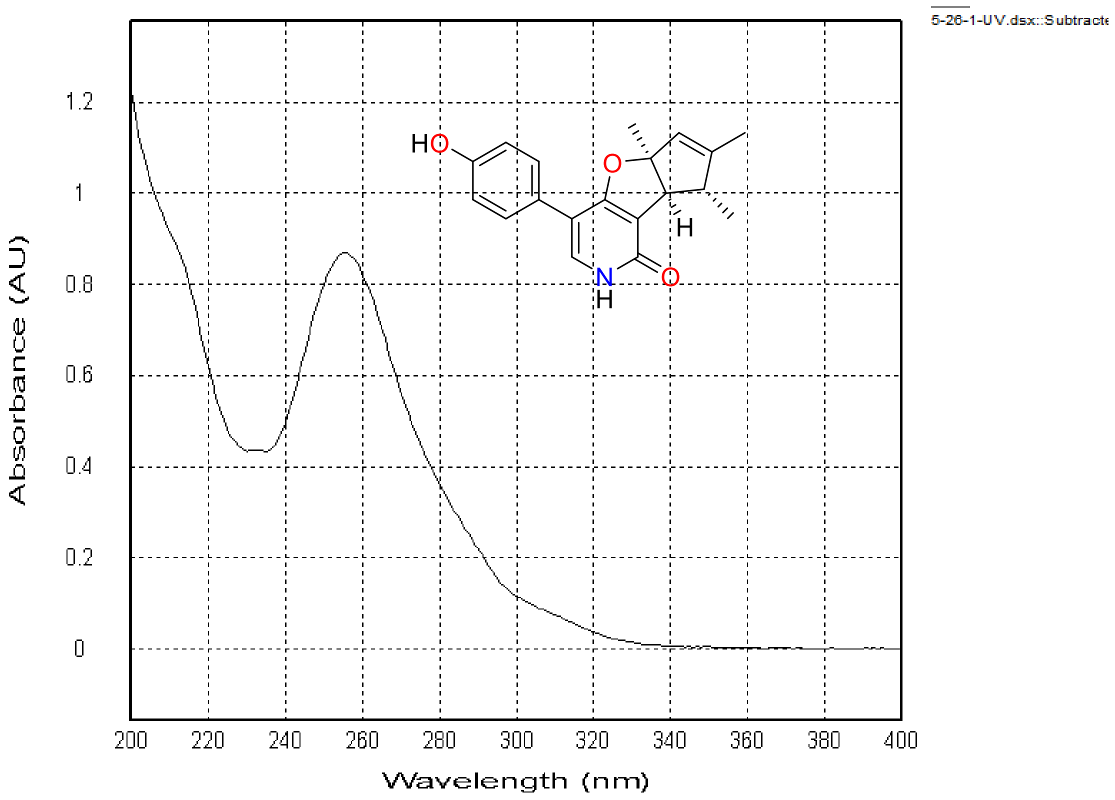

Figure S9. UV spectrum of **1a**

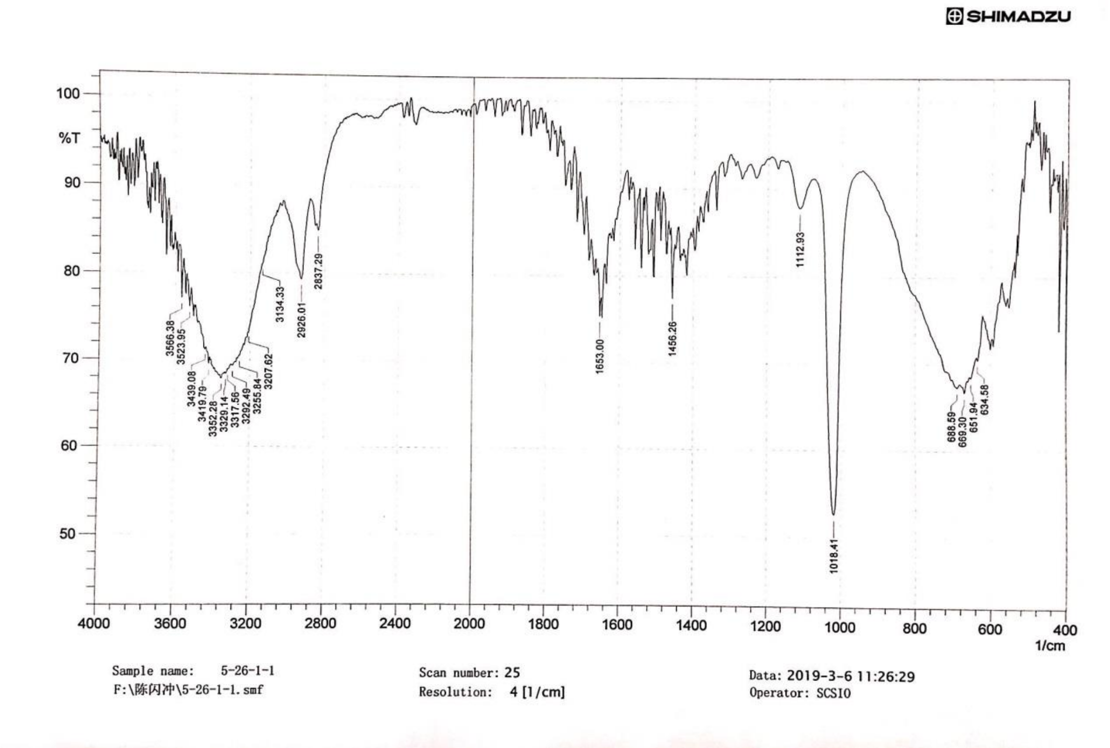

Figure S10. IR spectrum of **1a**

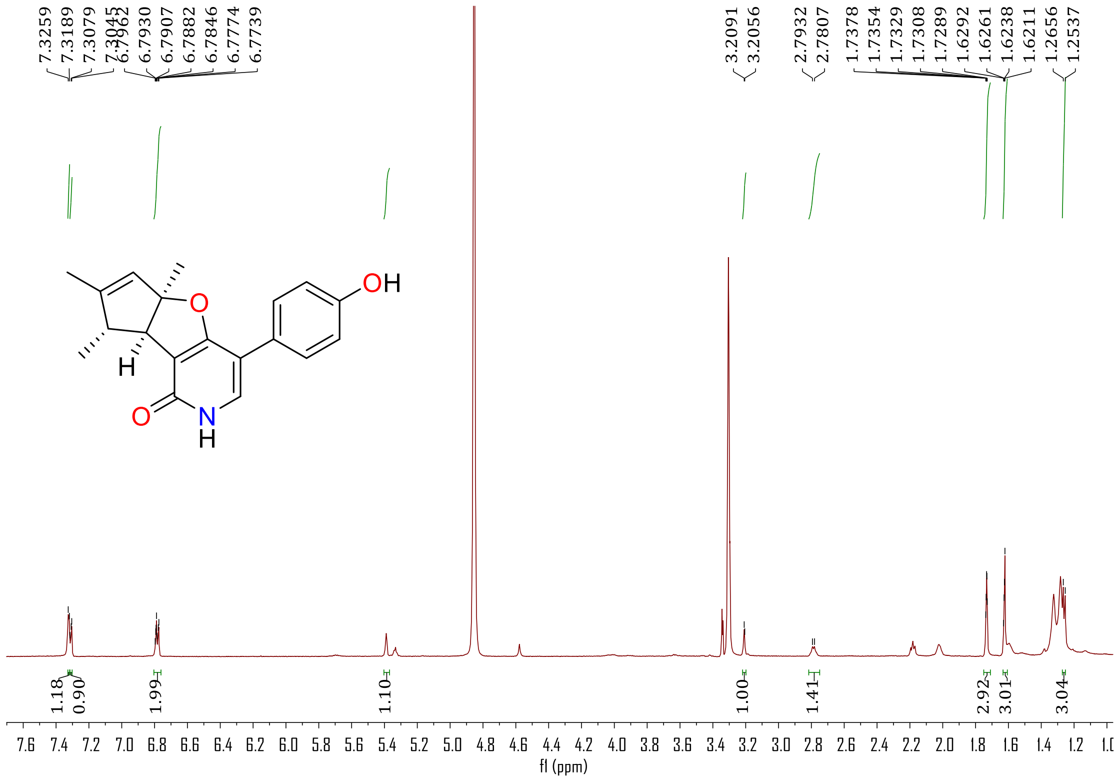

Figure S11. ^{1}H NMR spectrum (600 MHz, CD_3OD) of **1b**

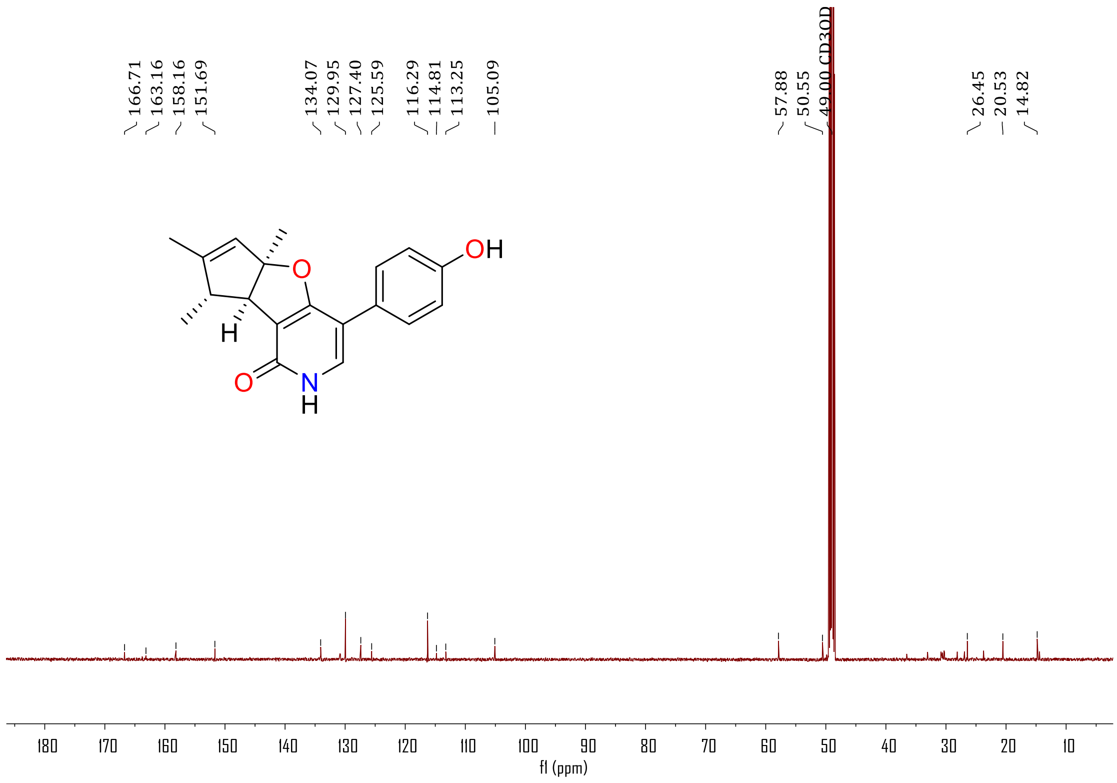

Figure S12. ^{13}C NMR spectrum (150 MHz, CD_3OD) of **1b**

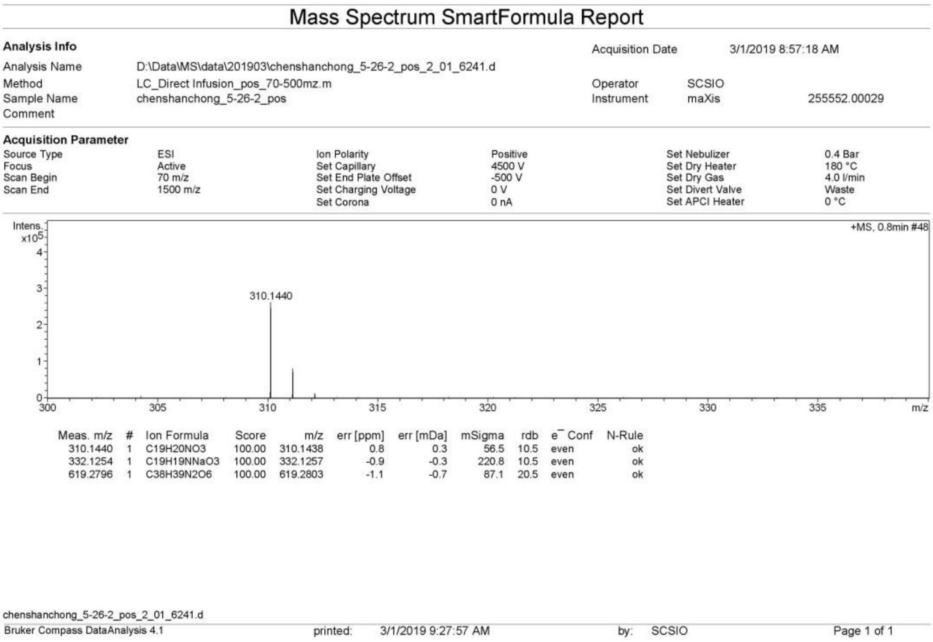

Mass Spectrum SmartFormula Report

Analysis Info

Analysis Name	D:\Data\MS\data\201903\chenshanchong_5-26-2_pos_2_01_6241.d	Acquisition Date	3/1/2019 8:57:18 AM
Method	LC_Direct Infusion_pos_70-500mz.m	Operator	SCSIO
Sample Name	chenshanchong_5-26-2_pos	Instrument	maXis 255552.00029
Comment			

Acquisition Parameter

Source Type	ESI	Ion Polarity	Positive	Set Nebulizer	0.4 Bar
Focus	Active	Set Capillary	4500 V	Set Dry Heater	180 °C
Scan Begin	70 m/z	Set End Plate Offset	-500 V	Set Dry Gas	4.0 l/min
Scan End	1500 m/z	Set Charging Voltage	0 V	Set Divert Valve	Waste
		Set Corona	0 nA	Set APCI Heater	0 °C

Meas. m/z	#	Ion Formula	Score	m/z	err [ppm]	err [mDa]	mSigma	rdb	e¯ Conf	N-Rule
310.1440	1	C19H20NO3	100.00	310.1438	0.8	0.3	56.5	10.5	even	ok
332.1254	1	C19H19NNaO3	100.00	332.1257	-0.9	-0.3	220.8	10.5	even	ok
619.2796	1	C38H39N2O6	100.00	619.2803	-1.1	-0.7	87.1	20.5	even	ok

chenshanchong_5-26-2_pos_2_01_6241.d
Bruker Compass DataAnalysis 4.1 printed: 3/1/2019 9:27:57 AM by: SCSIO Page 1 of 1

Figure S13. HRESIMS spectrum of **1b**

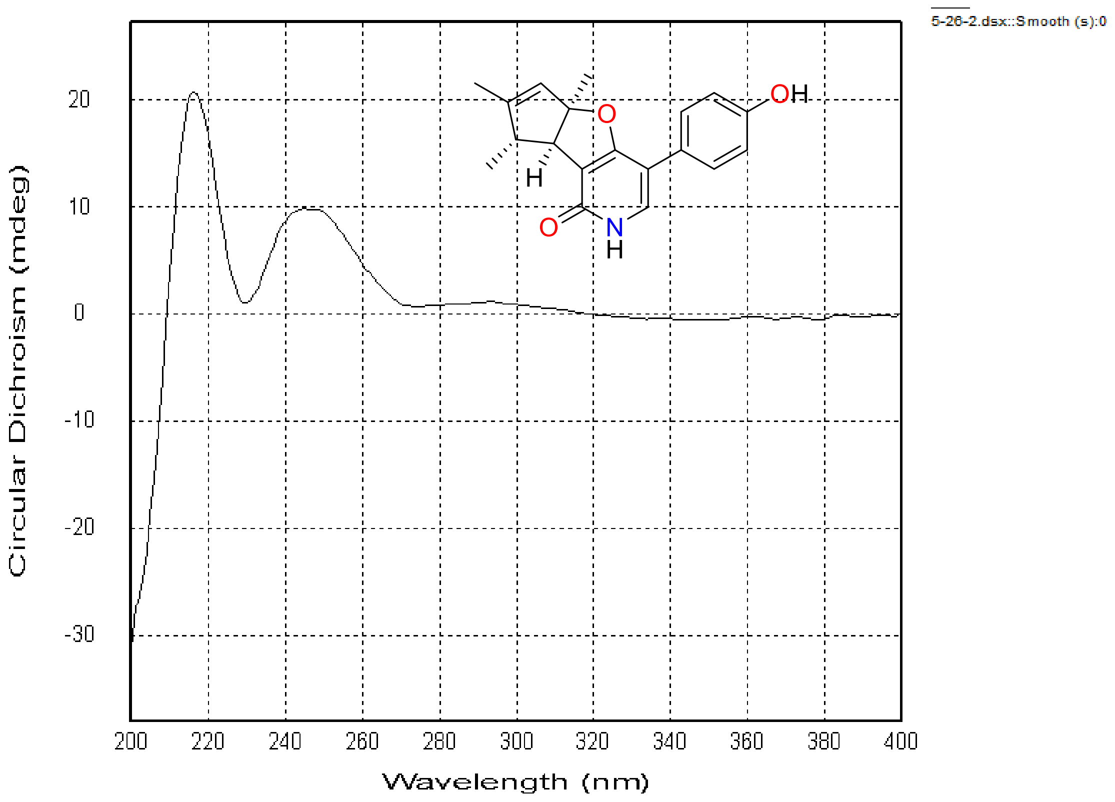

Figure S14. CD spectrum of **1b**

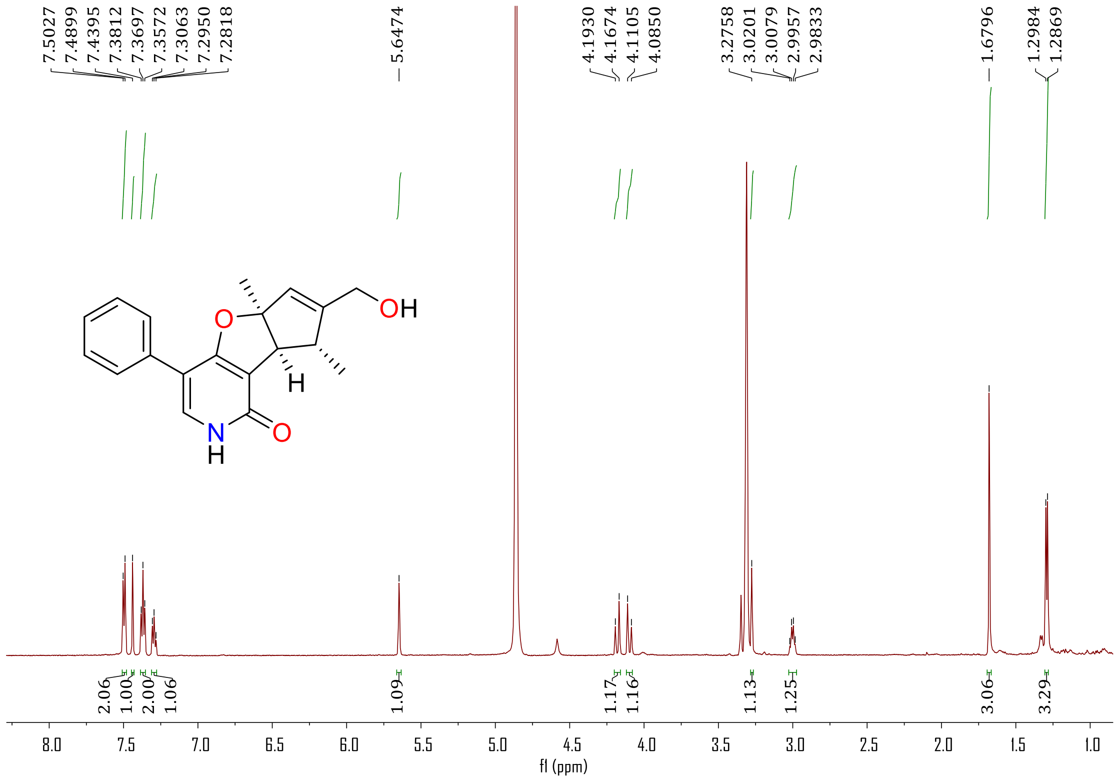

Figure S15. ^{1}H NMR spectrum (600 MHz, CD_3OD) of **2a**

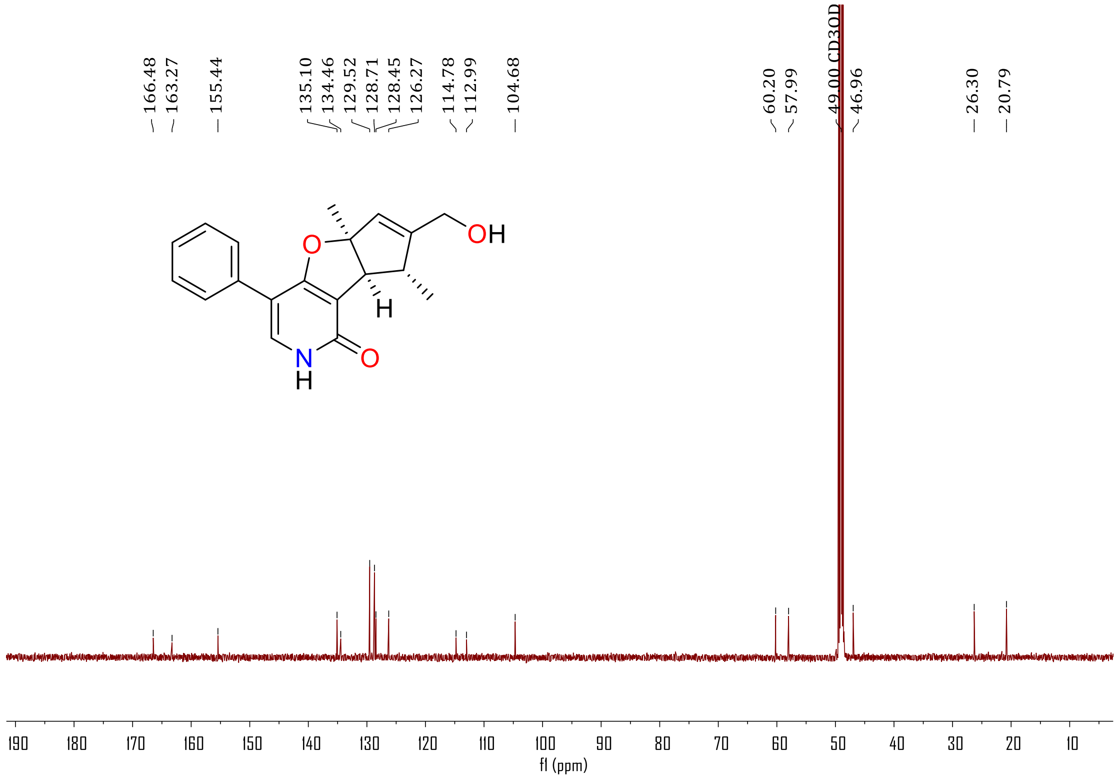

Figure S16. ^{13}C NMR spectrum (150 MHz, CD_3OD) of **2a**

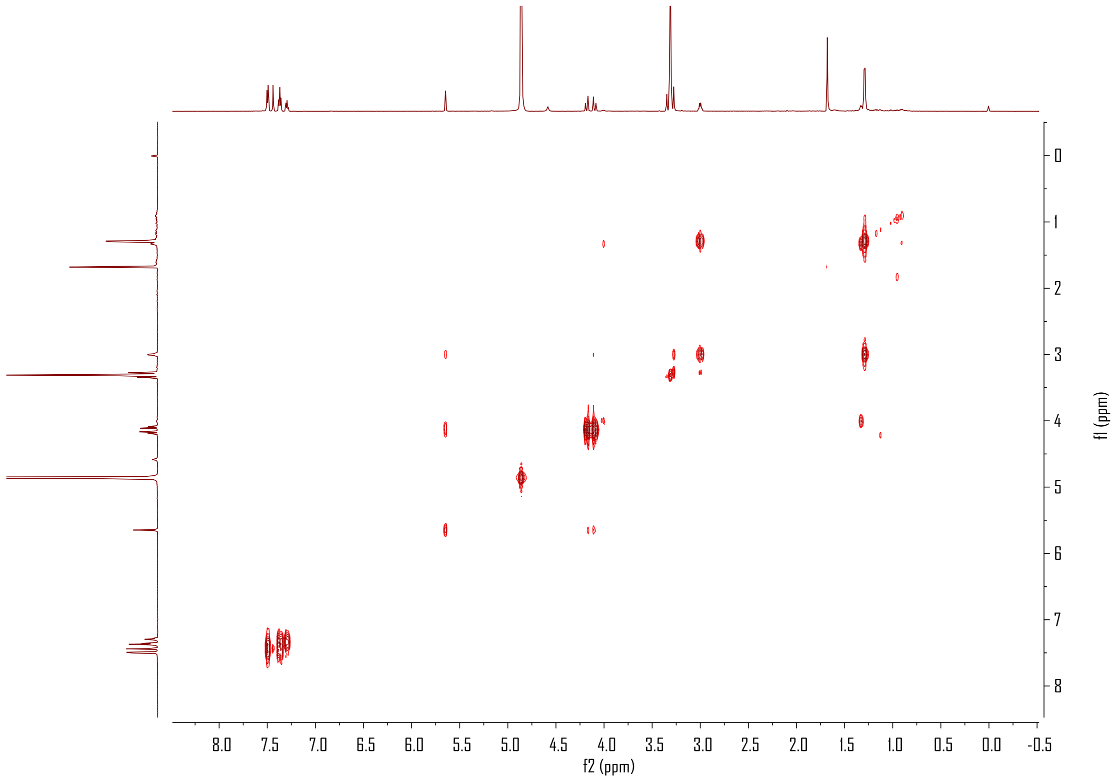

Figure S17. ^{1}H-^{1}H COSY spectrum of **2a** in CD_3OD

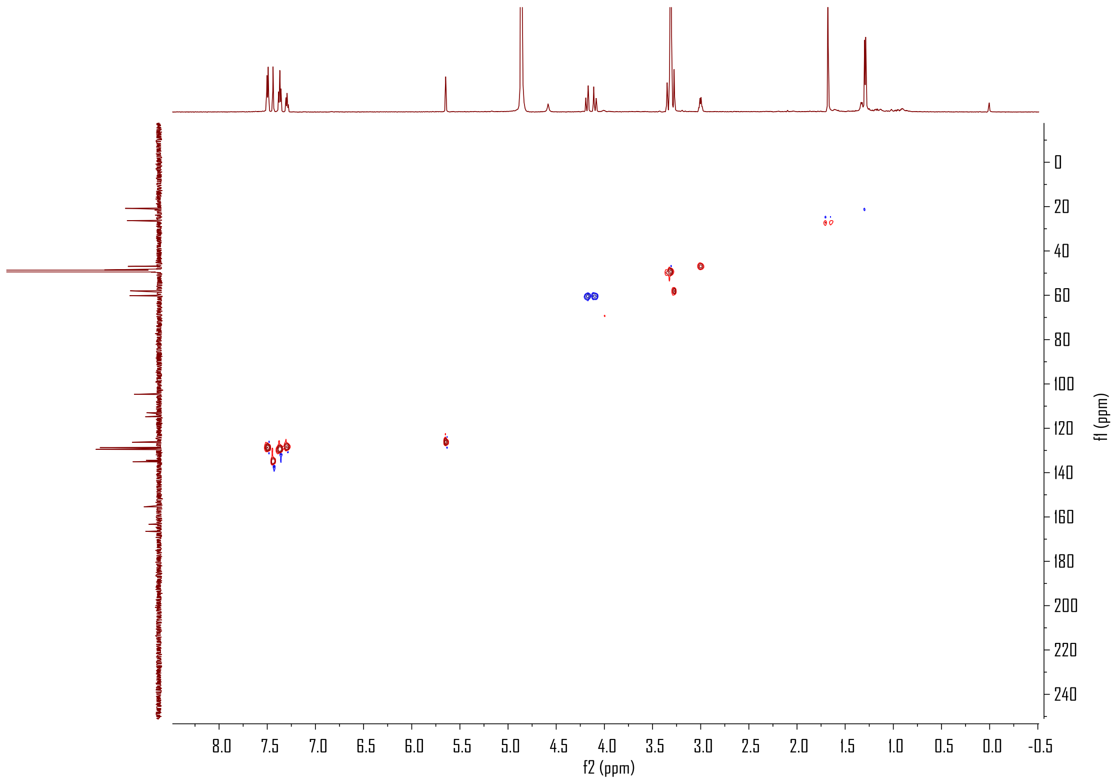

Figure S18. HSQC spectrum of **2a** in CD_3OD

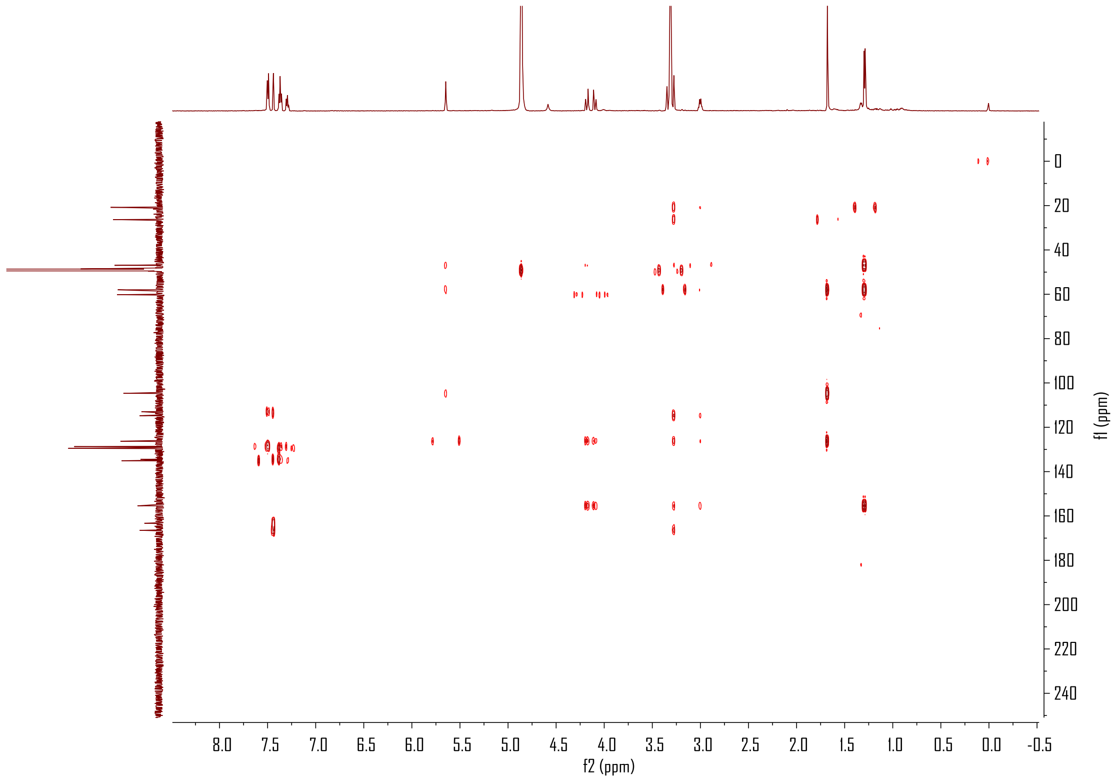

Figure S19. HMBC spectrum of **2a** in CD_3OD

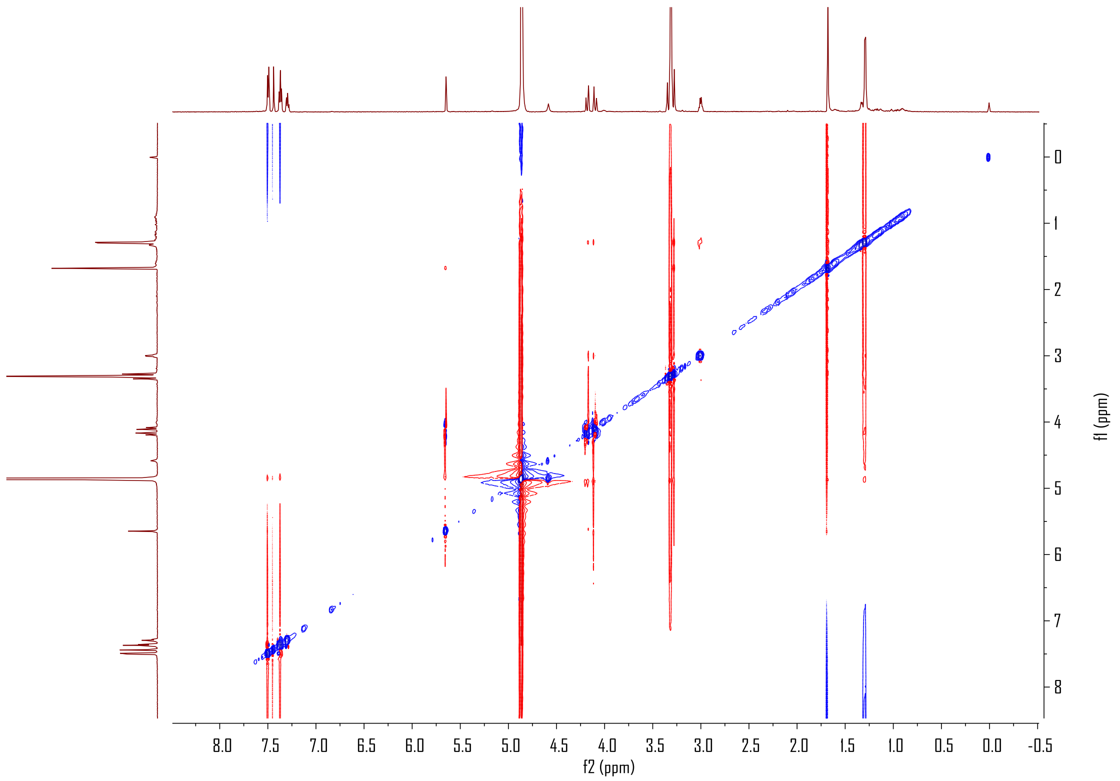

Figure S20. NOESY spectrum of **2a** in CD_3OD

Mass Spectrum SmartFormula Report

Analysis Info

Analysis Name	D:\Data\MS\data\201903\chenshanchong_5-42-2_pos_8_01_6247.d	Acquisition Date	3/1/2019 9:18:03 AM	
Method	LC_Direct Infusion_pos_70-500mz.m	Operator	SCSIO	
Sample Name	chenshanchong_5-42-2_pos	Instrument	maXis	255552.00029
Comment				

Acquisition Parameter

Source Type	ESI	Ion Polarity	Positive	Set Nebulizer	0.4 Bar
Focus	Active	Set Capillary	4500 V	Set Dry Heater	180 °C
Scan Begin	70 m/z	Set End Plate Offset	-500 V	Set Dry Gas	4.0 l/min
Scan End	1500 m/z	Set Charging Voltage	0 V	Set Divert Valve	Waste
		Set Corona	0 nA	Set APCI Heater	0 °C

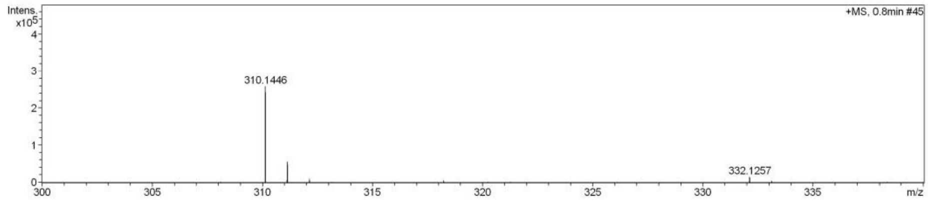

Meas. m/z	#	Ion Formula	Score	m/z	err [ppm]	err [mDa]	mSigma	rdb	e⁻ Conf	N-Rule
310.1446	1	C19H20NO3	100.00	310.1438	-2.7	-0.8	2.5	10.5	even	ok
332.1257	1	C19H19NNaO3	100.00	332.1257	-0.0	-0.0	29.6	10.5	even	ok
619.2813	1	C38H39N2O6	100.00	619.2803	1.6	1.0	32.0	20.5	even	ok
641.2631	1	C38H38N2NaO6	100.00	641.2622	-1.4	-0.9	44.9	20.5	even	ok

chenshanchong_5-42-2_pos_8_01_6247.d

Bruker Compass DataAnalysis 4.1 printed: 3/1/2019 9:54:03 AM by: SCSIO Page 1 of 1

Figure S21. HRESIMS spectrum of **2a**

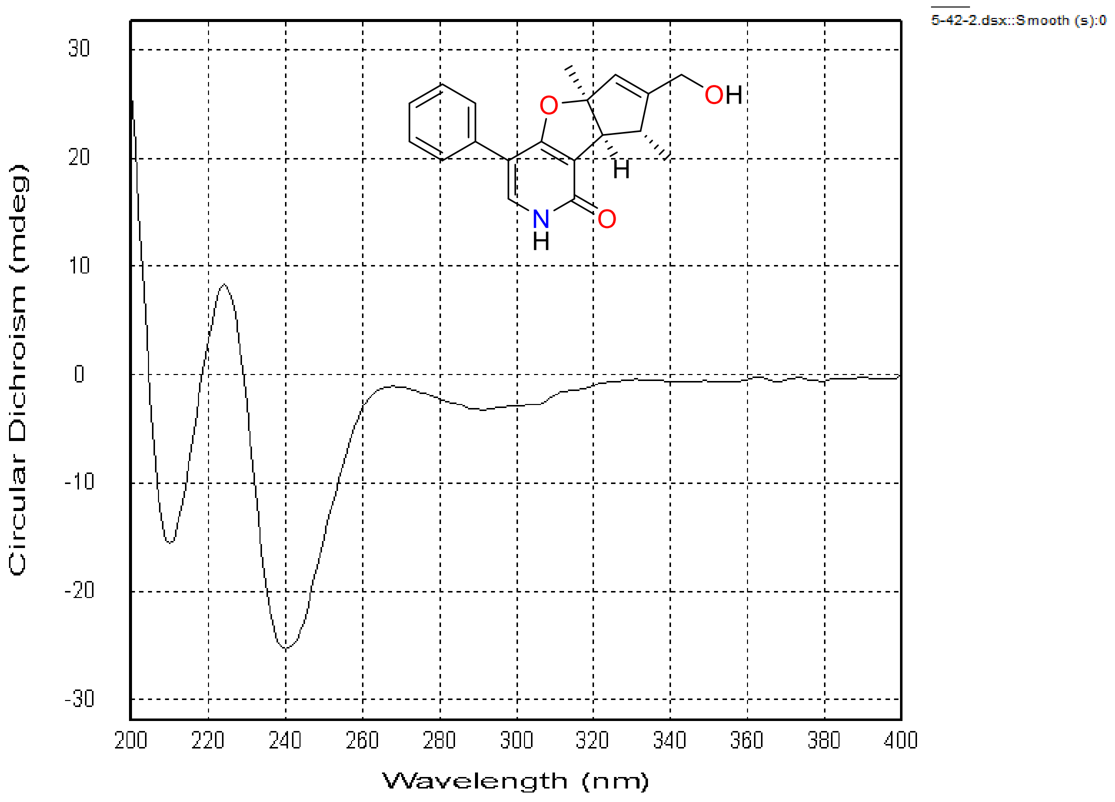

Figure S22. CD spectrum of **2a**

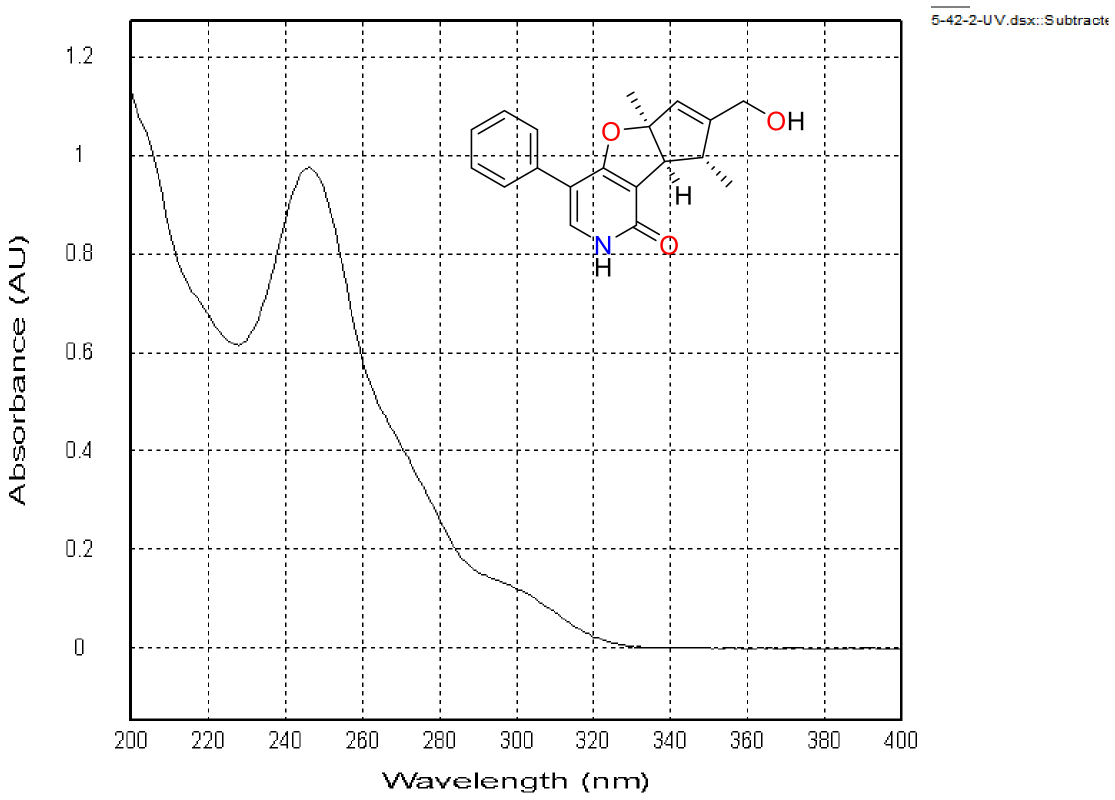

Figure S23. UV spectrum of **2a**

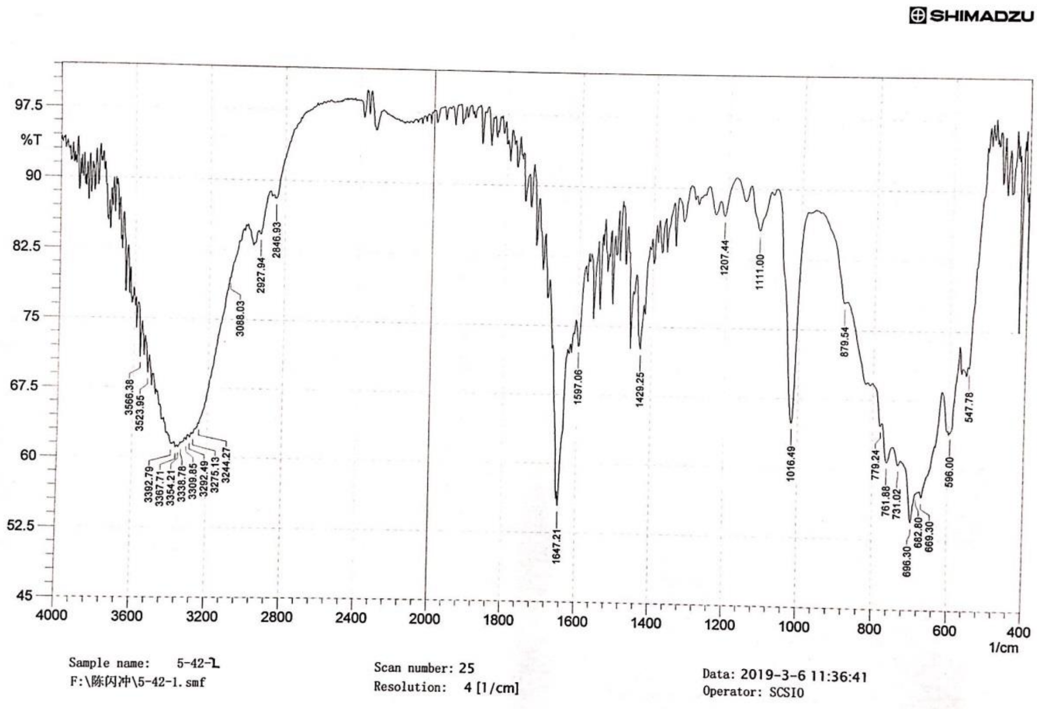

Figure S24. IR spectrum of **2a**

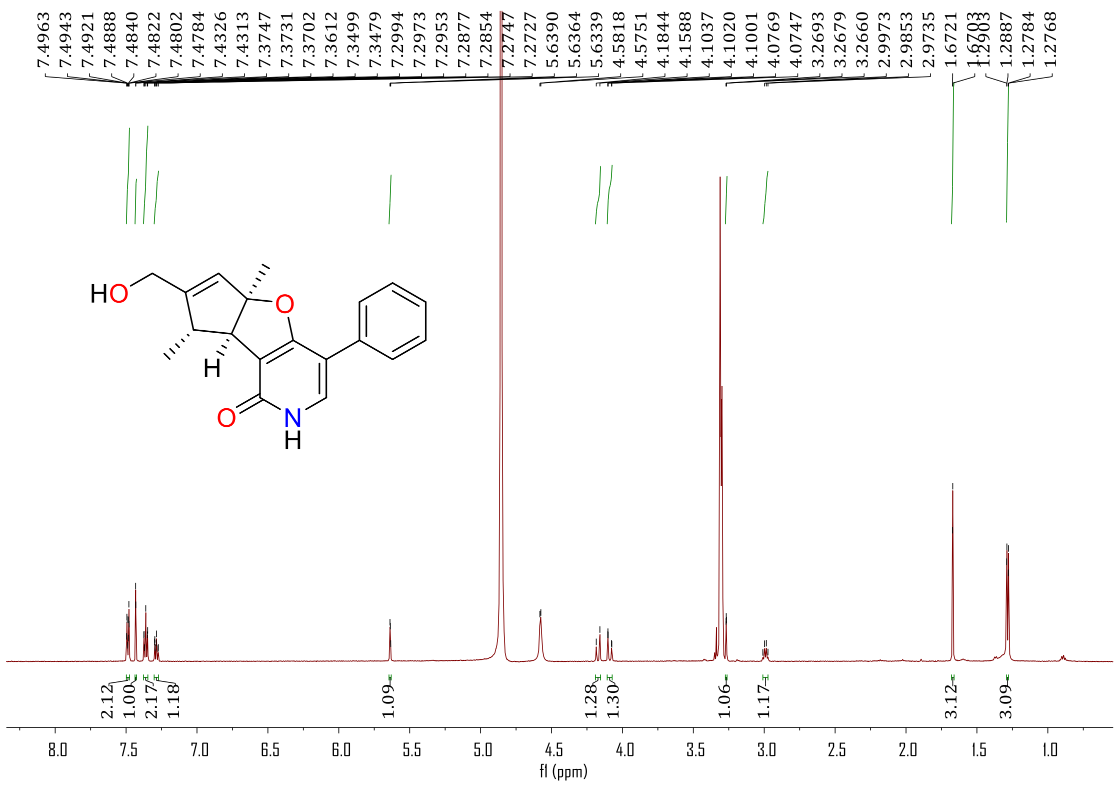

Figure S25. ^{1}H NMR spectrum (600 MHz, CD_3OD) of **2b**

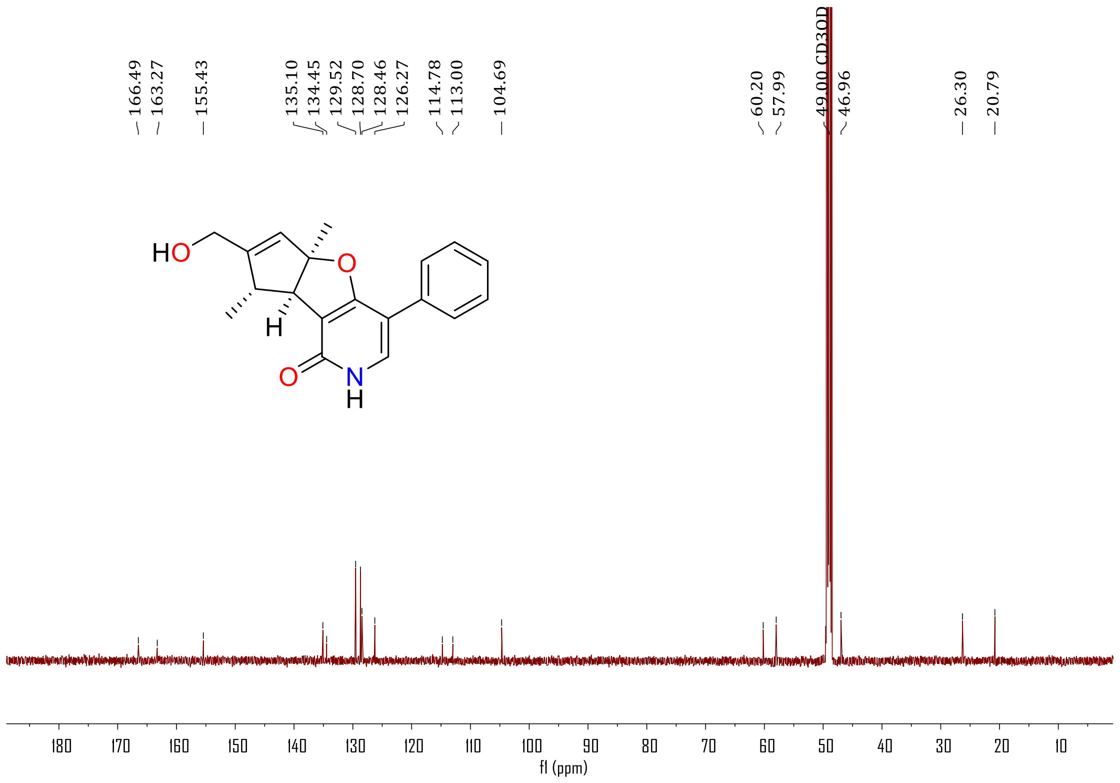

Figure S26. ^{13}C NMR spectrum (150 MHz, CD_3OD) of **2b**

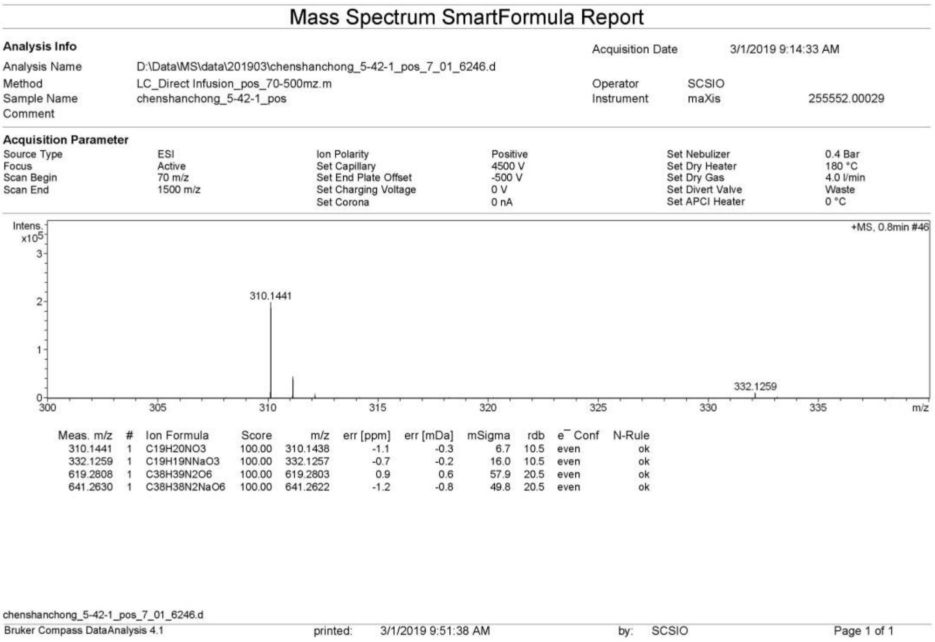

Mass Spectrum SmartFormula Report

Analysis Info

Analysis Name D:\Data\MS\data\201903\chenshanchong_5-42-1_pos_7_01_6246.d
Method LC_Direct Infusion_pos_70-500mz.m
Sample Name chenshanchong_5-42-1_pos
Comment

Acquisition Date 3/1/2019 9:14:33 AM
Operator SCSIO
Instrument maXis 255552.00029

Acquisition Parameter

Source Type	ESI	Ion Polarity	Positive	Set Nebulizer	0.4 Bar
Focus	Active	Set Capillary	4500 V	Set Dry Heater	180 °C
Scan Begin	70 m/z	Set End Plate Offset	-500 V	Set Dry Gas	4.0 l/min
Scan End	1500 m/z	Set Charging Voltage	0 V	Set Divert Valve	Waste
		Set Corona	0 nA	Set APCI Heater	0 °C

Meas. m/z	#	Ion Formula	Score	m/z	err [ppm]	err [mDa]	mSigma	rdb	e⁻ Conf	N-Rule
310.1441	1	C19H20NO3	100.00	310.1438	-1.1	-0.3	6.7	10.5	even	ok
332.1259	1	C19H19NNaO3	100.00	332.1257	-0.7	-0.2	16.0	10.5	even	ok
619.2808	1	C38H39N2O6	100.00	619.2803	0.9	0.6	57.9	20.5	even	ok
641.2630	1	C38H38N2NaO6	100.00	641.2622	-1.2	-0.8	49.8	20.5	even	ok

chenshanchong_5-42-1_pos_7_01_6246.d
Bruker Compass DataAnalysis 4.1 printed: 3/1/2019 9:51:38 AM by: SCSIO Page 1 of 1

Figure S27. HRESIMS spectrum of **2b**

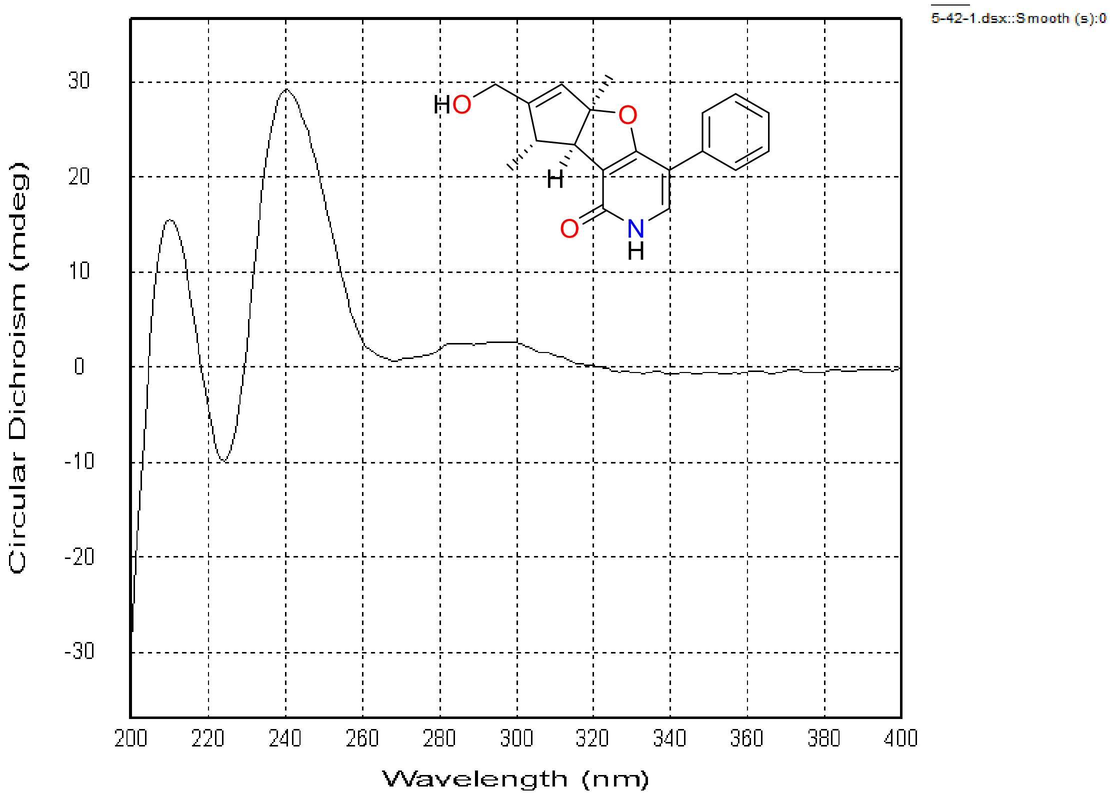

Figure S28. CD spectrum of **2b**

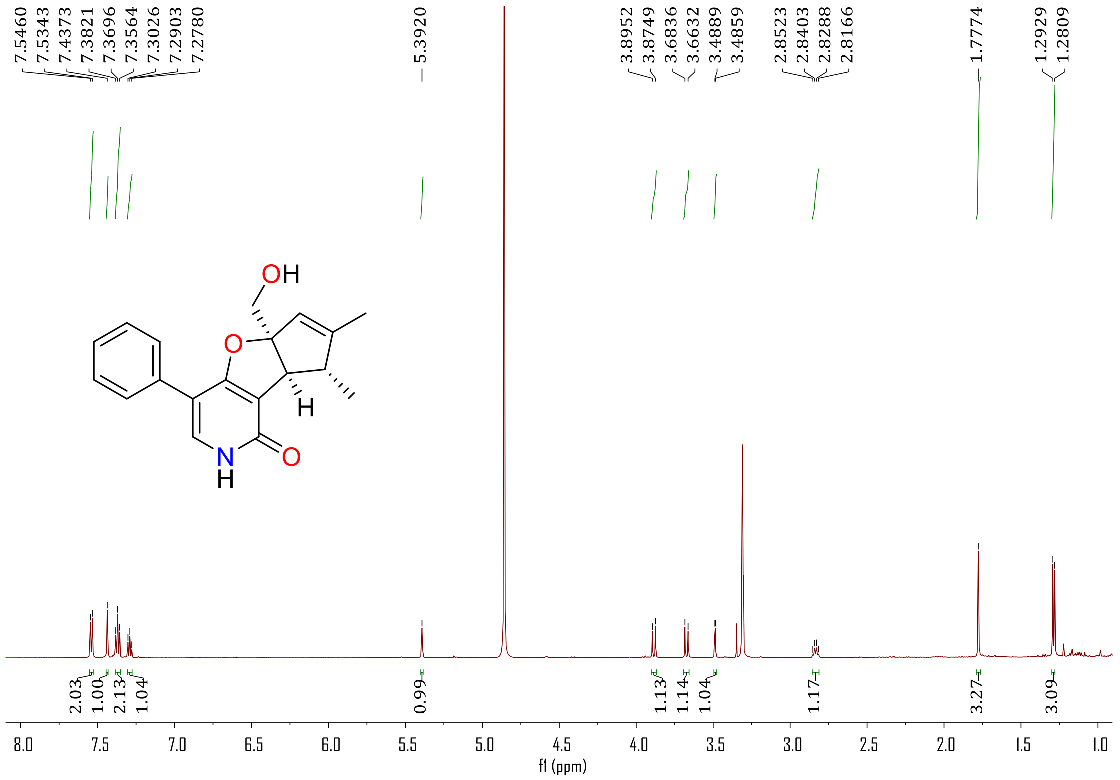

Figure S29. ^{1}H NMR spectrum (600 MHz, CD_3OD) of **3a**

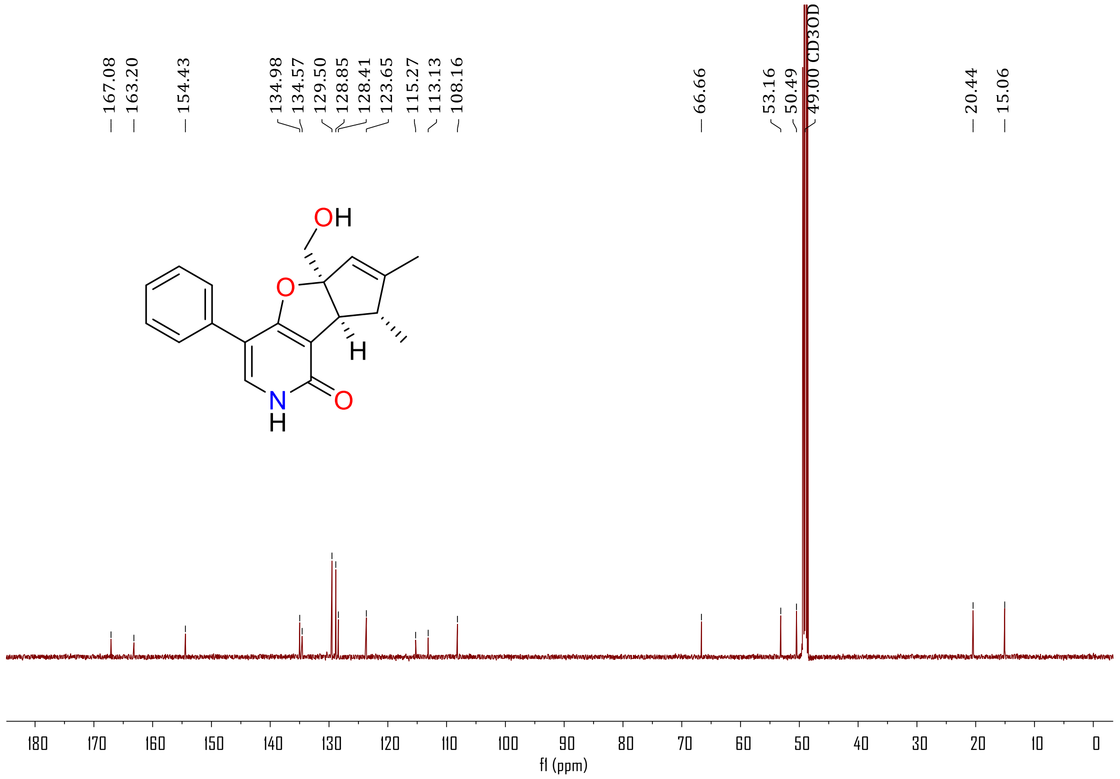

Figure S30. ^{13}C NMR spectrum (150 MHz, CD_3OD) of **3a**

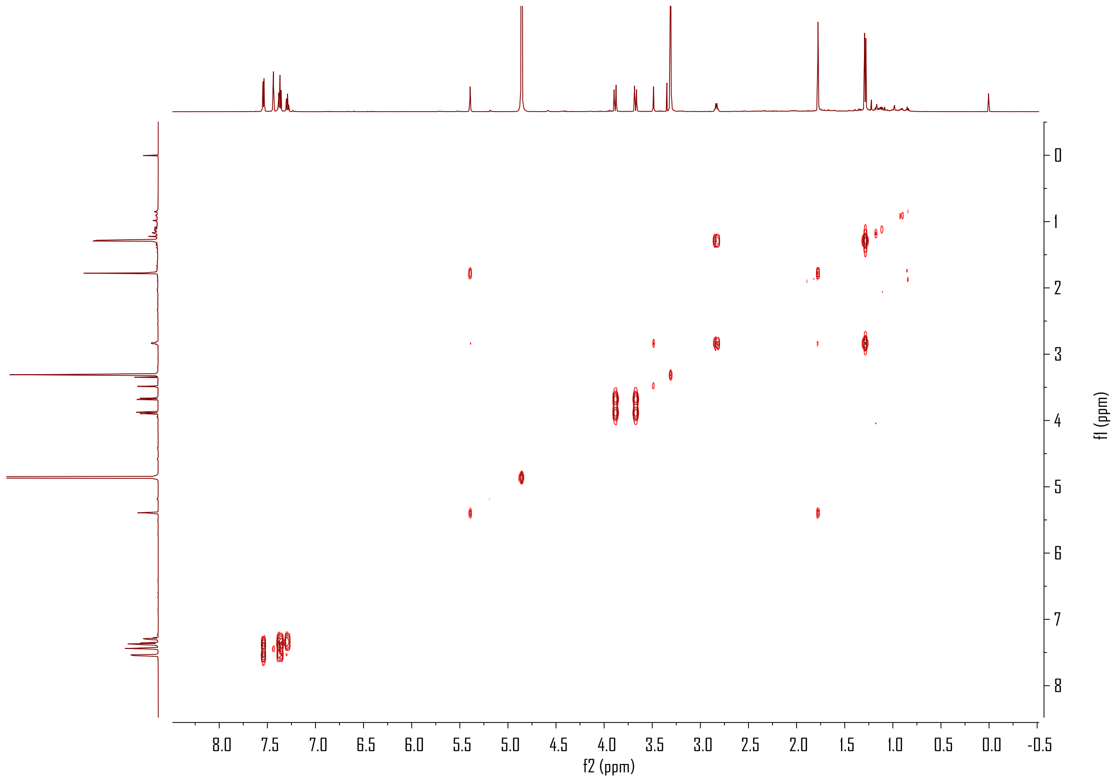

Figure S31. ^{1}H-^{1}H COSY spectrum of **3a** in CD_3OD

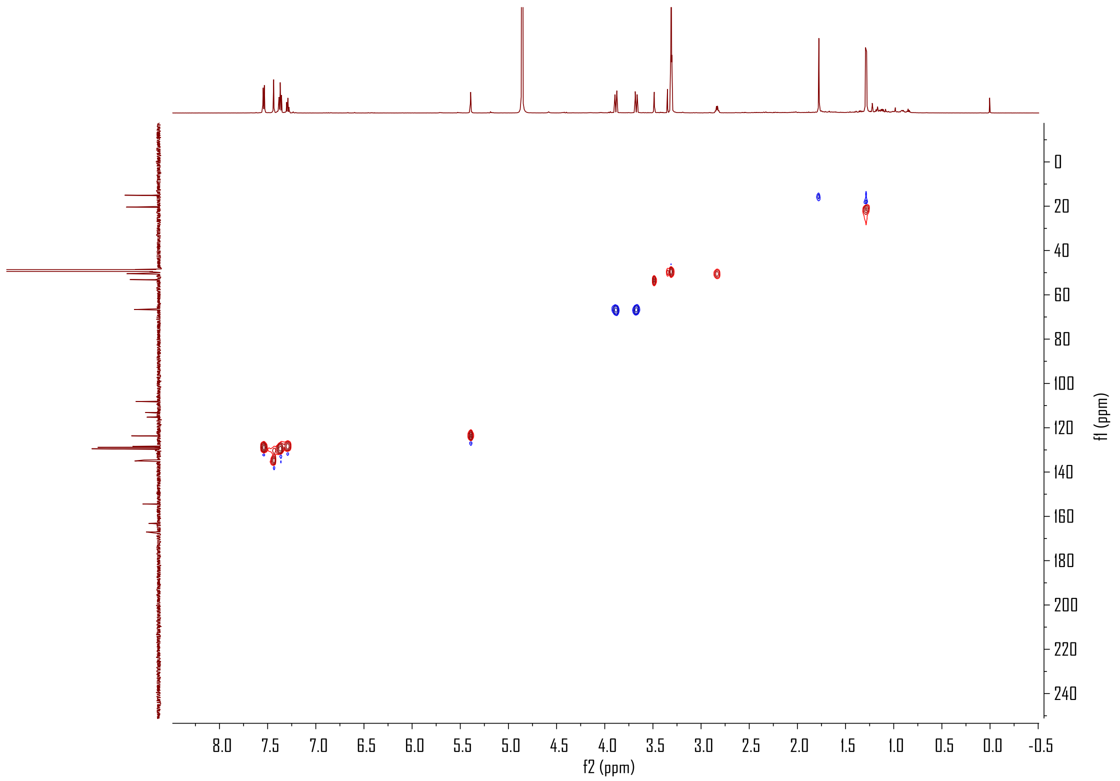

Figure S32. HSQC spectrum of **3a** in CD_3OD

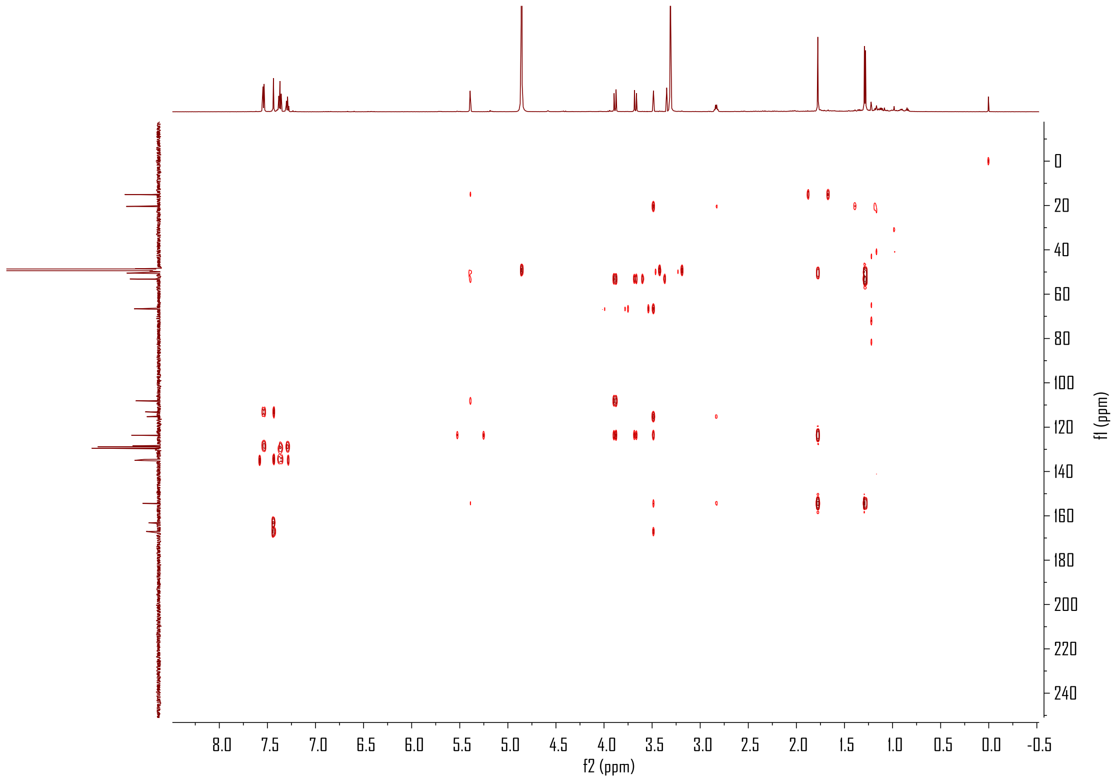

Figure S33. HMBC spectrum of **3a** in CD_3OD

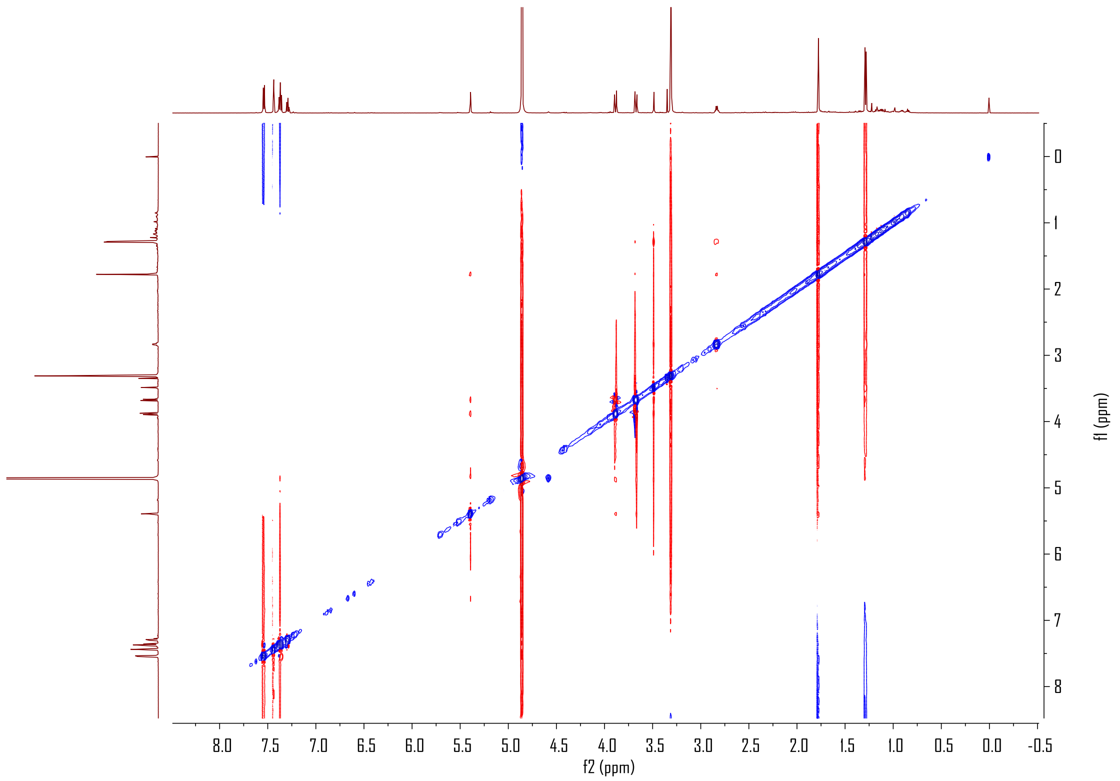

Figure S34. NOESY spectrum of **3a** in CD_3OD

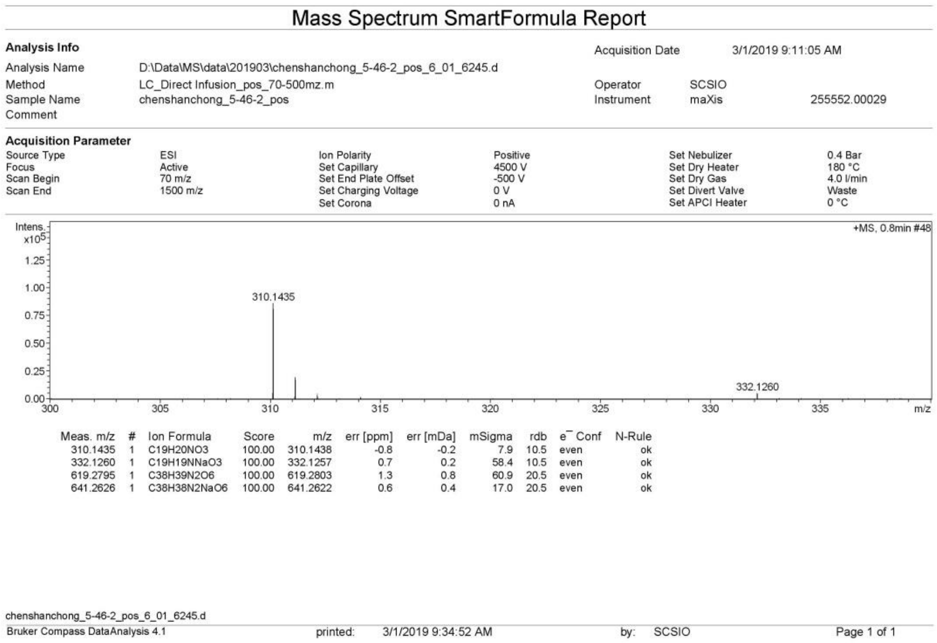

Mass Spectrum SmartFormula Report

Analysis Info

Analysis Name	D:\Data\MS\data\201903\chenshanchong_5-46-2_pos_6_01_6245.d	Acquisition Date	3/1/2019 9:11:05 AM		
Method	LC_Direct Infusion_pos_70-500mz.m	Operator	SCSIO		
Sample Name	chenshanchong_5-46-2_pos	Instrument	maXis	255552.00029	
Comment					

Acquisition Parameter

Source Type	ESI	Ion Polarity	Positive	Set Nebulizer	0.4 Bar
Focus	Active	Set Capillary	4500 V	Set Dry Heater	180 °C
Scan Begin	70 m/z	Set End Plate Offset	-500 V	Set Dry Gas	4.0 l/min
Scan End	1500 m/z	Set Charging Voltage	0 V	Set Divert Valve	Waste
		Set Corona	0 nA	Set APCI Heater	0 °C

Meas. m/z	#	Ion Formula	Score	m/z	err [ppm]	err [mDa]	mSigma	rdb	e⁻ Conf	N-Rule
310.1435	1	C19H20NO3	100.00	310.1438	-0.8	-0.2	7.9	10.5	even	ok
332.1260	1	C19H19NNaO3	100.00	332.1257	0.7	0.2	58.4	10.5	even	ok
619.2795	1	C38H39N2O6	100.00	619.2803	1.3	0.8	60.9	20.5	even	ok
641.2626	1	C38H38N2NaO6	100.00	641.2622	0.6	0.4	17.0	20.5	even	ok

chenshanchong_5-46-2_pos_6_01_6245.d

Bruker Compass DataAnalysis 4.1 printed: 3/1/2019 9:34:52 AM by: SCSIO Page 1 of 1

Figure S35. HRESIMS spectrum of **3a**

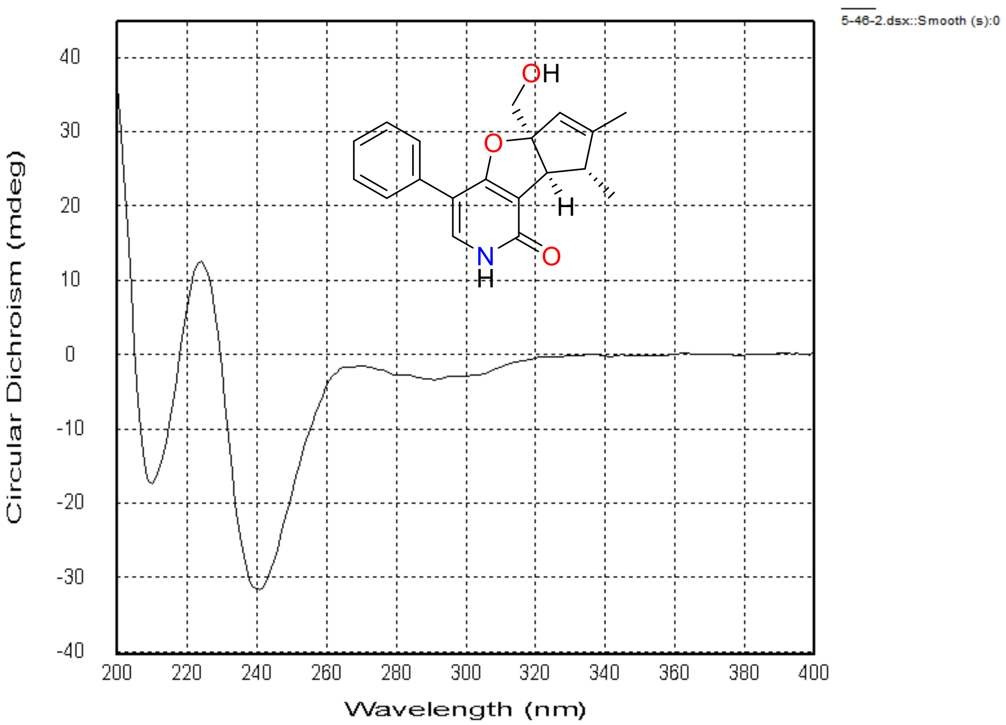

Figure S36. CD spectrum of **3a**

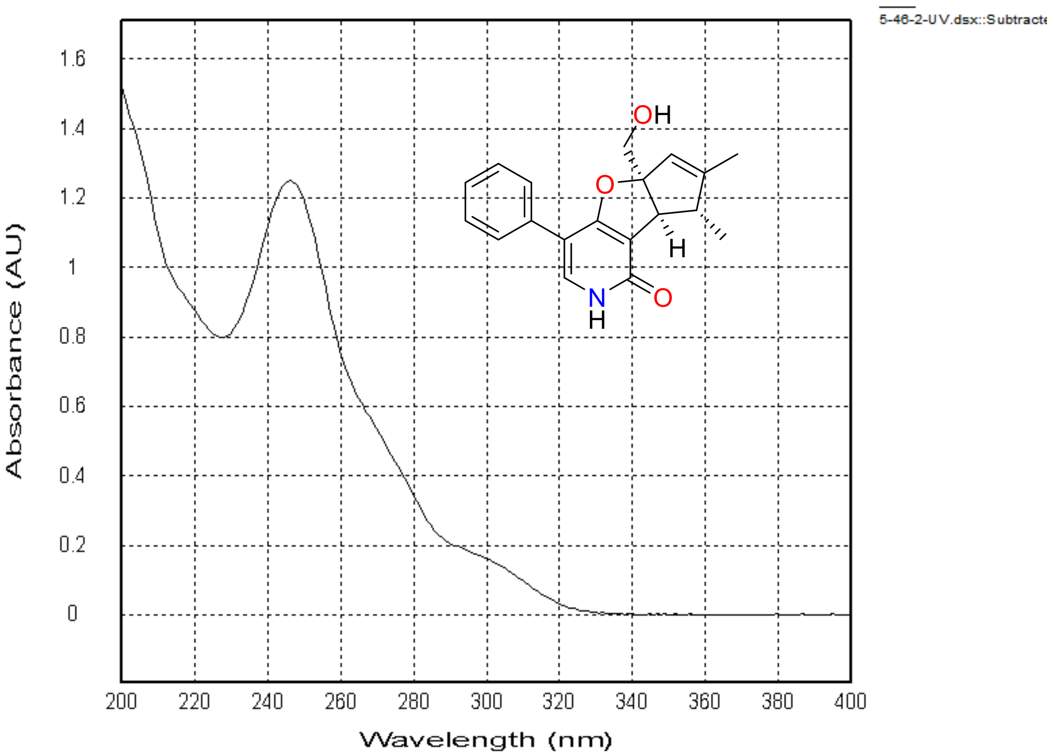

Figure S37. UV spectrum of **3a**

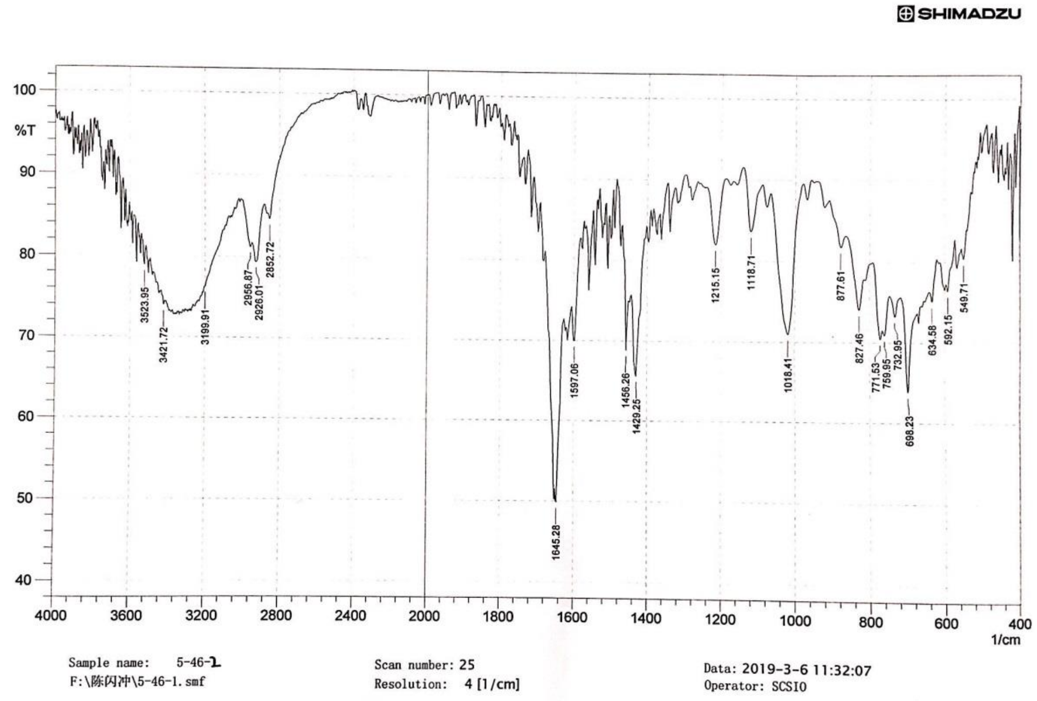

Figure S38. IR spectrum of **3a**

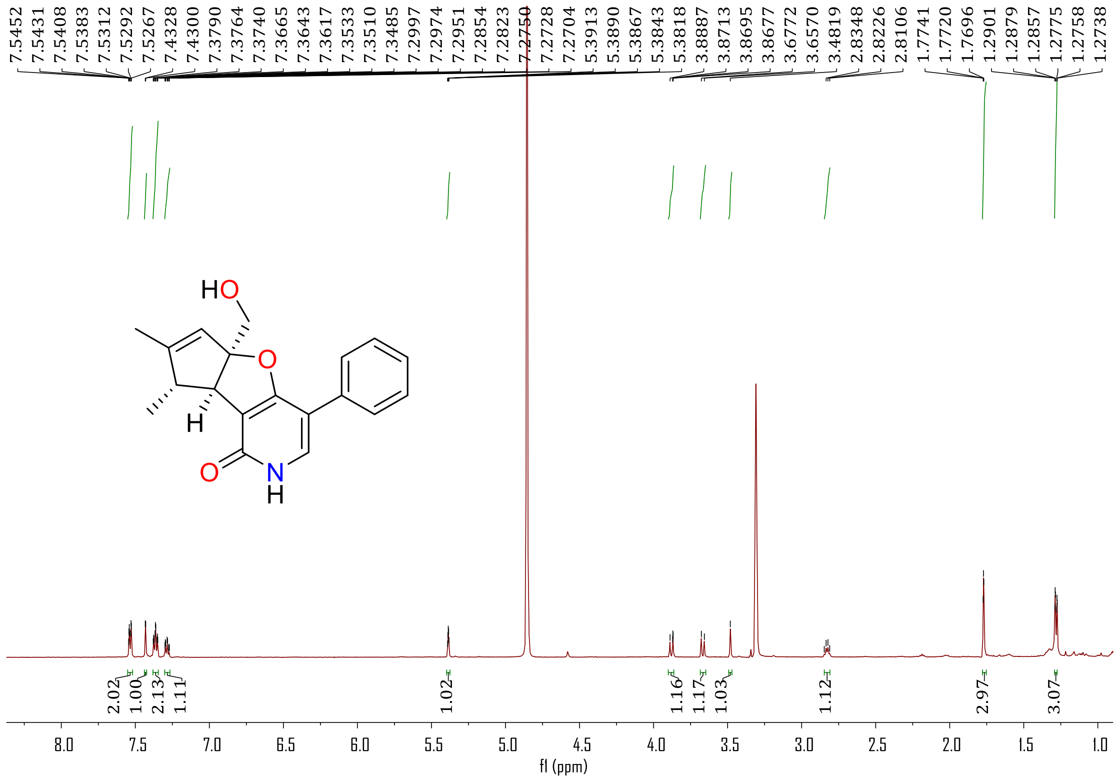

Figure S39. ^{1}H NMR spectrum (600 MHz, CD_3OD) of **3b**

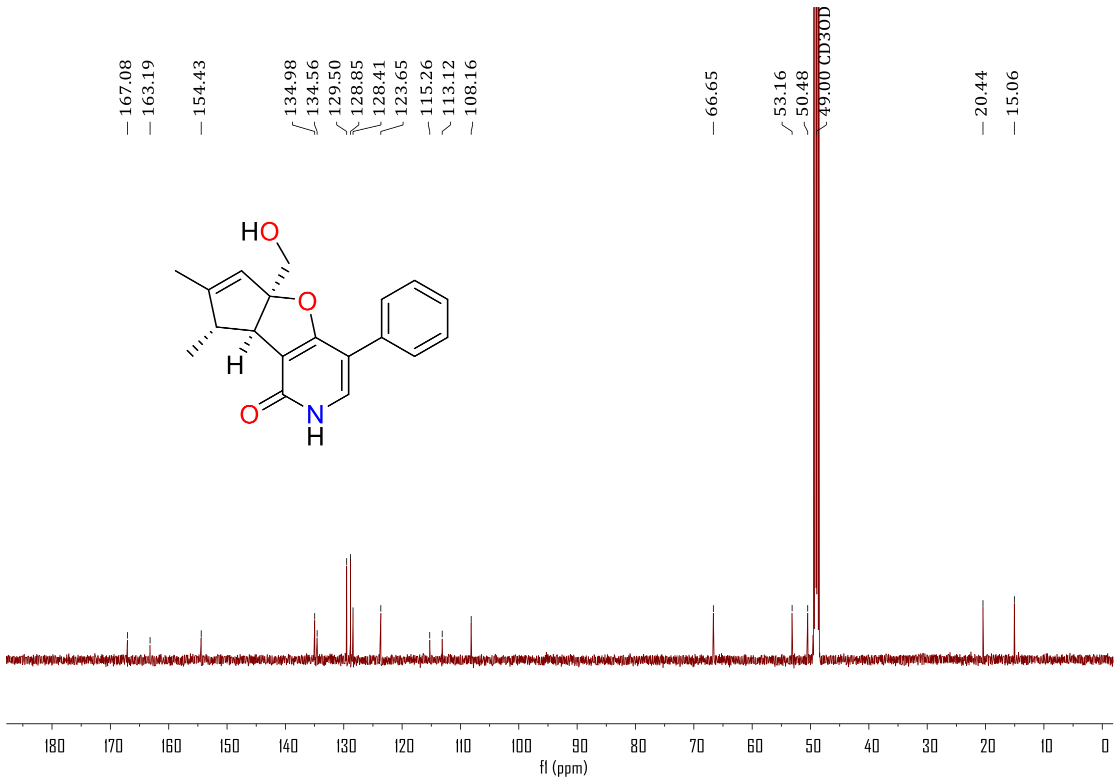

Figure S40. ^{13}C NMR spectrum (150 MHz, CD_3OD) of **3b**

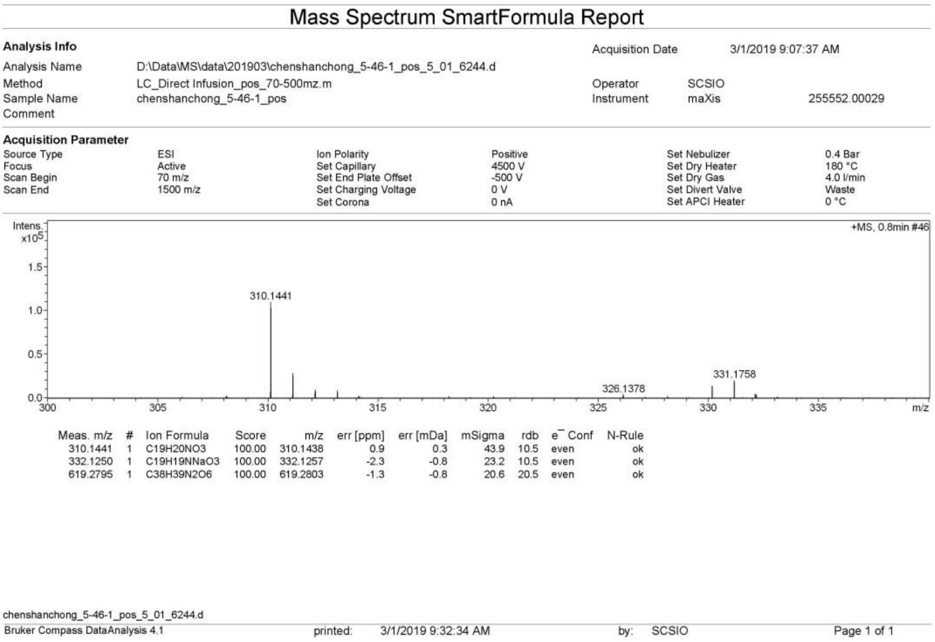

Mass Spectrum SmartFormula Report

Analysis Info

Analysis Name	D:\Data\MS\data\201903\chenshanchong_5-46-1_pos_5_01_6244.d	Acquisition Date	3/1/2019 9:07:37 AM
Method	LC_Direct Infusion_pos_70-500mz.m	Operator	SCSIO
Sample Name	chenshanchong_5-46-1_pos	Instrument	maXis 255552.00029
Comment			

Acquisition Parameter

Source Type	ESI	Ion Polarity	Positive	Set Nebulizer	0.4 Bar
Focus	Active	Set Capillary	4500 V	Set Dry Heater	180 °C
Scan Begin	70 m/z	Set End Plate Offset	-500 V	Set Dry Gas	4.0 l/min
Scan End	1500 m/z	Set Charging Voltage	0 V	Set Divert Valve	Waste
		Set Corona	0 nA	Set APCI Heater	0 °C

Meas. m/z	#	Ion Formula	Score	m/z	err [ppm]	err [mDa]	mSigma	rdb	e⁻ Conf	N-Rule
310.1441	1	C19H20NO3	100.00	310.1438	0.9	0.3	43.9	10.5	even	ok
332.1250	1	C19H19NNaO3	100.00	332.1257	-2.3	-0.8	23.2	10.5	even	ok
619.2795	1	C38H39N2O6	100.00	619.2803	-1.3	-0.8	20.6	20.5	even	ok

chenshanchong_5-46-1_pos_5_01_6244.d
Bruker Compass DataAnalysis 4.1 printed: 3/1/2019 9:32:34 AM by: SCSIO Page 1 of 1

Figure S41. HRESIMS spectrum of **3b**

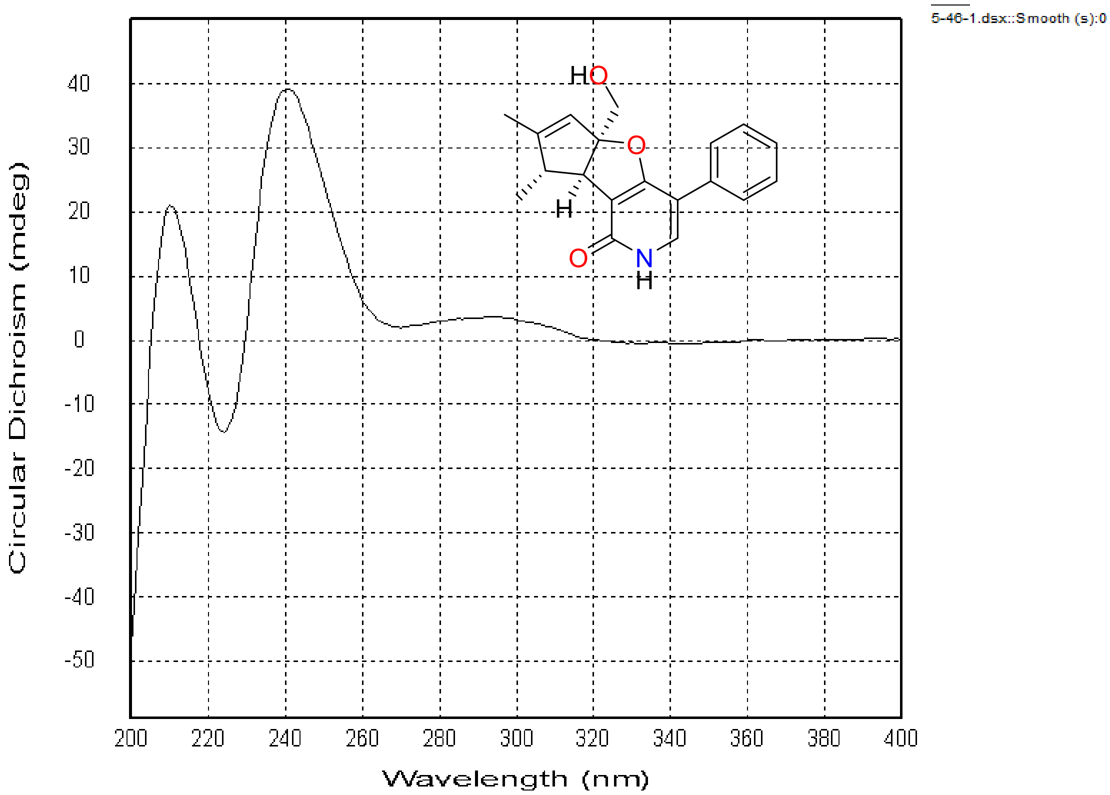

Figure S42. CD spectrum of **3b**

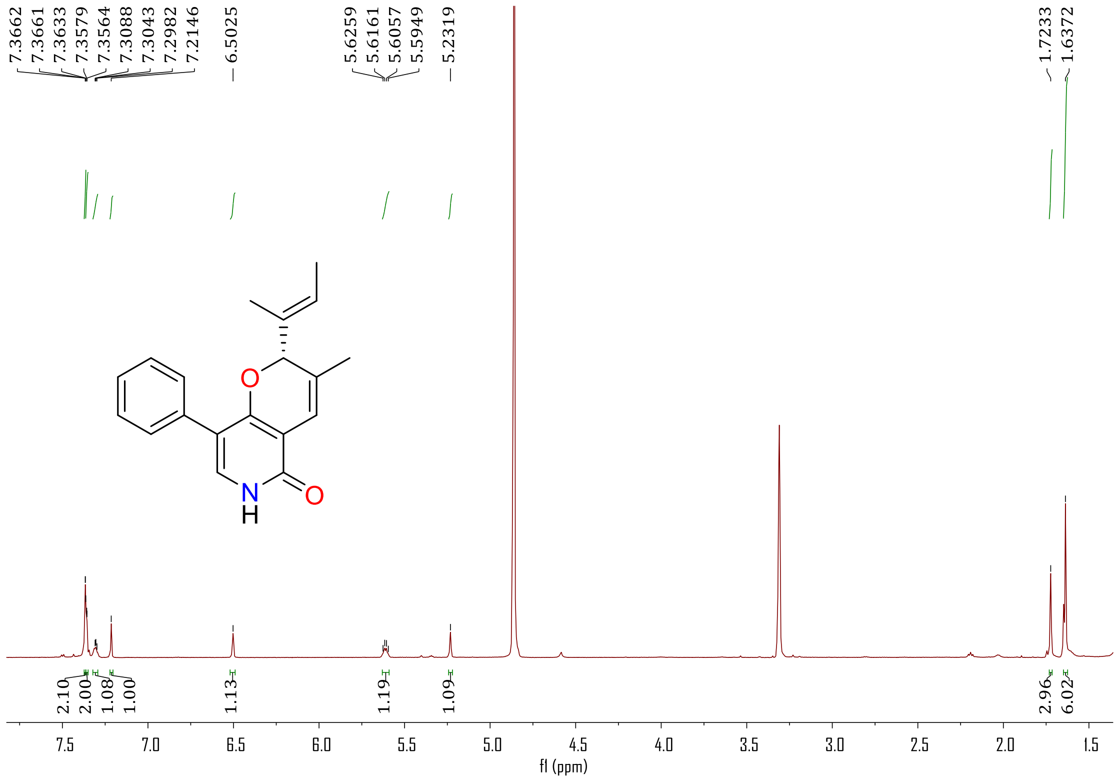

Figure S43. ^{1}H NMR spectrum (600 MHz, CD_3OD) of **4a**

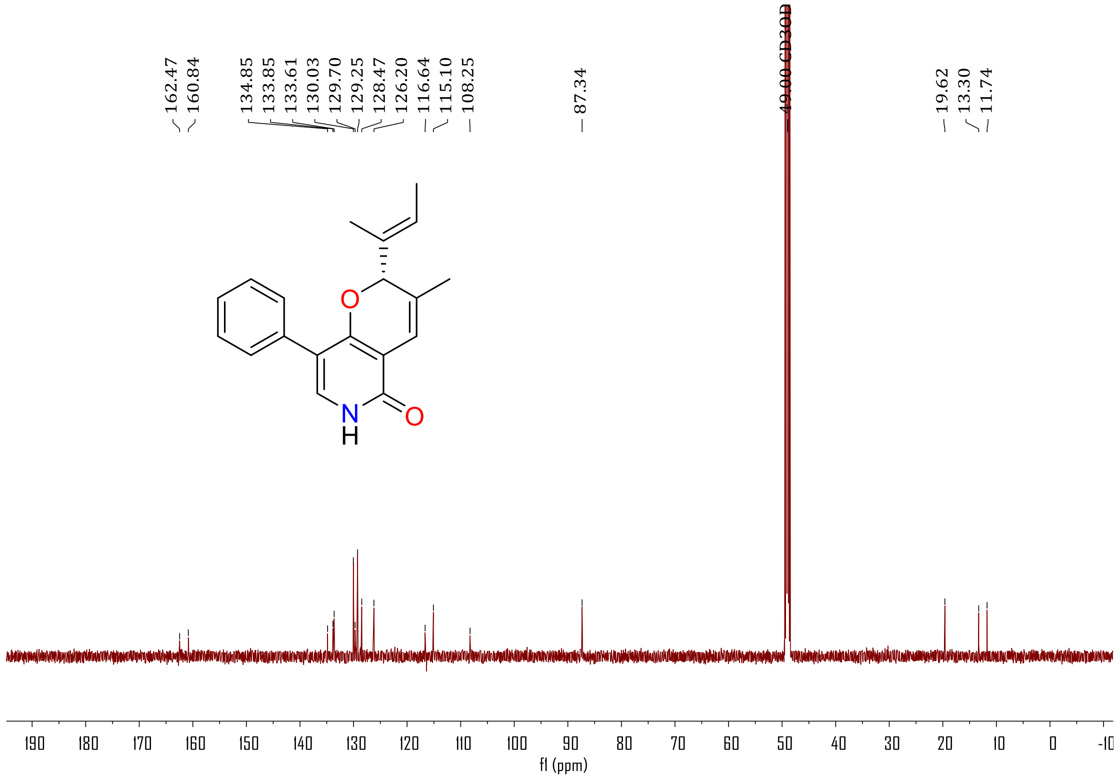

Figure S44. ^{13}C NMR spectrum (150 MHz, CD_3OD) of **4a**

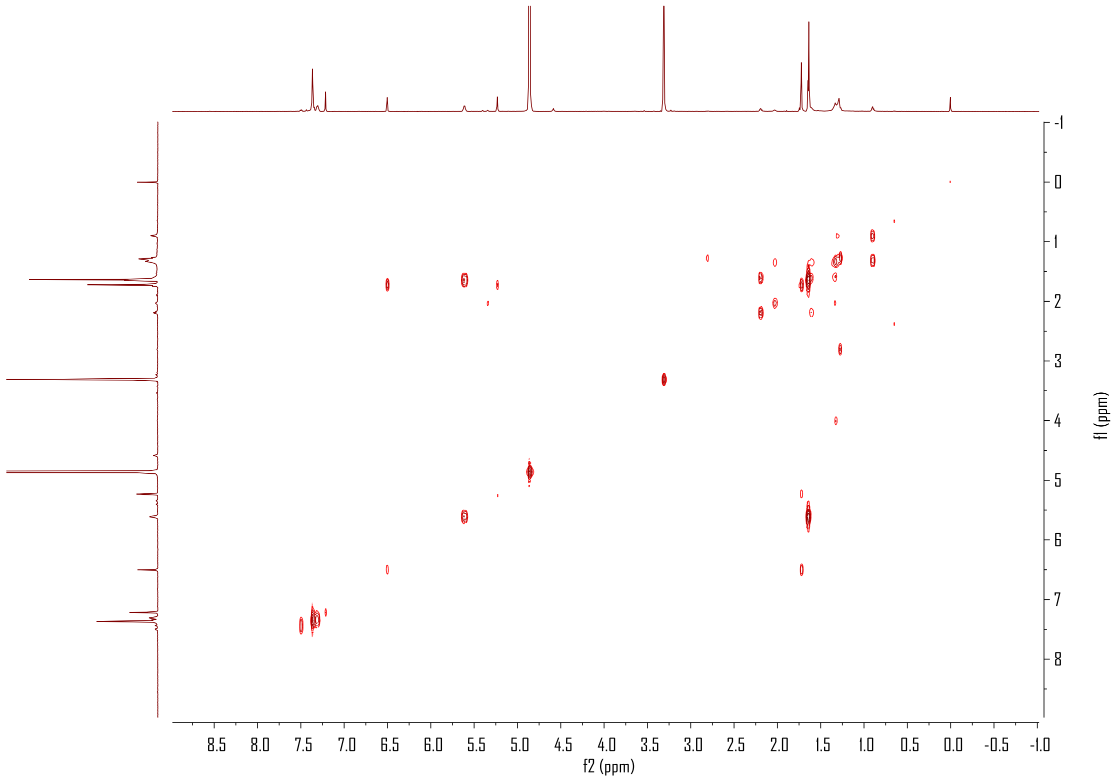

Figure S45. ${}^{1}H$-${}^{1}H$ COSY spectrum of **4a** in CD_3OD

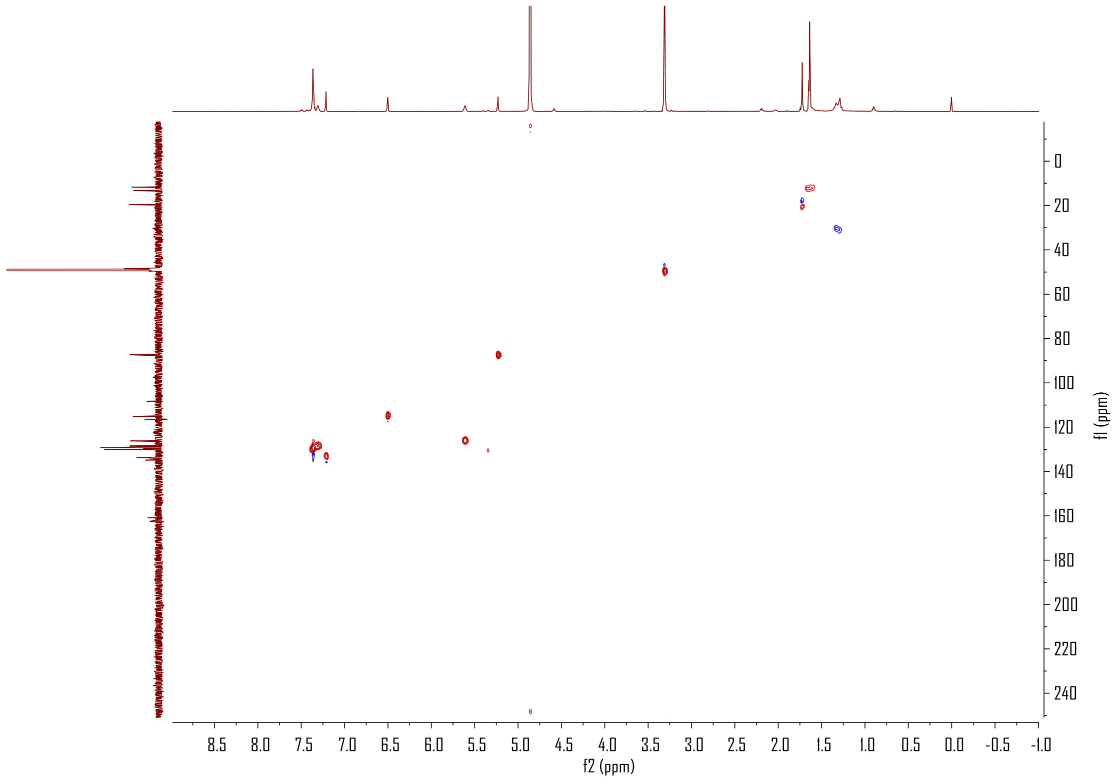

Figure S46. HSQC spectrum of **4a** in CD_3OD

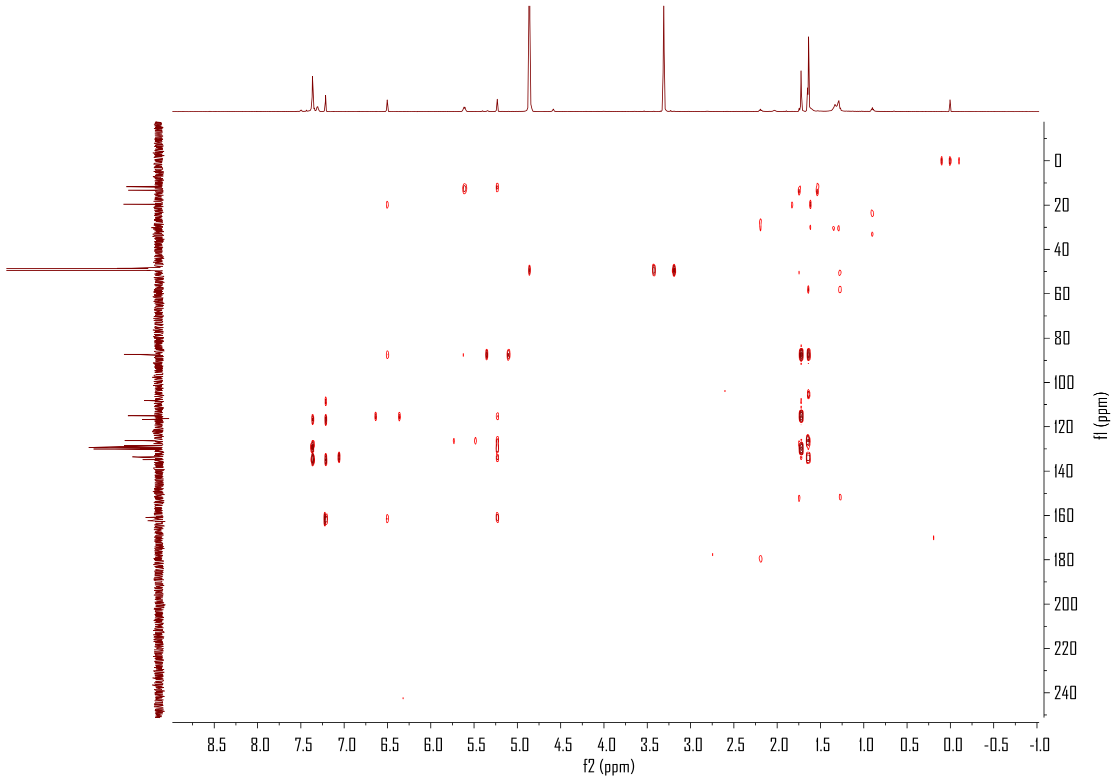

Figure S47. HMBC spectrum of **4a** in CD_3OD

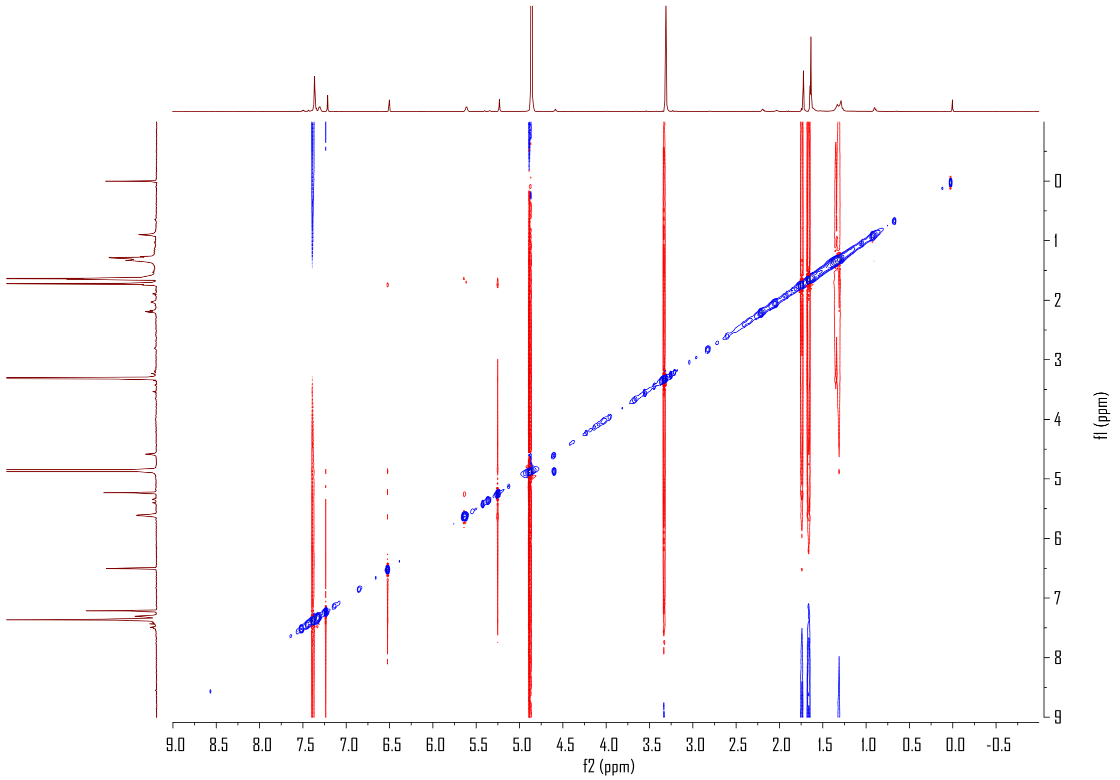

Figure S48. NOESY spectrum of **4a** in CD_3OD

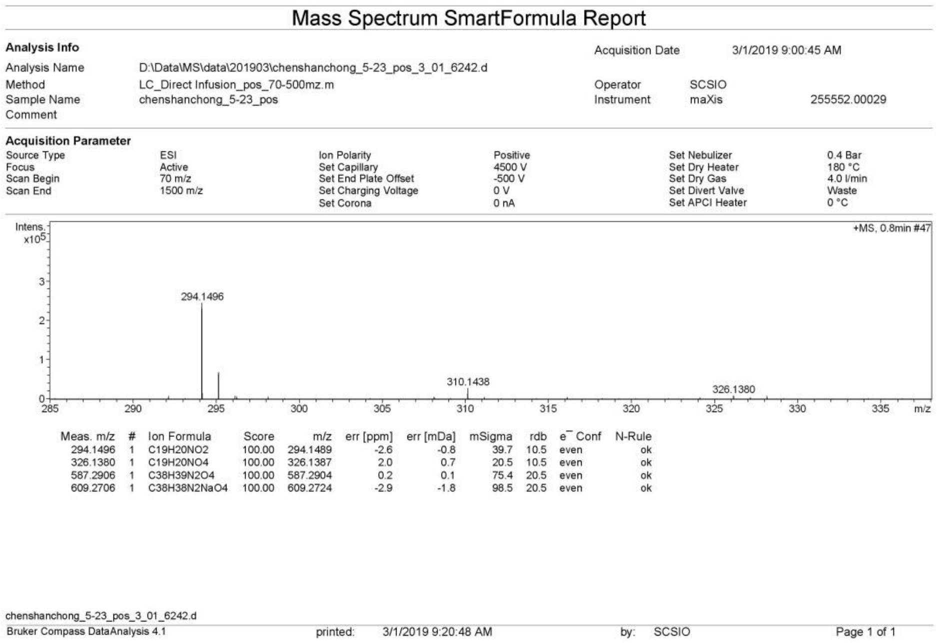

Figure S49. HRESIMS spectrum of **4a**

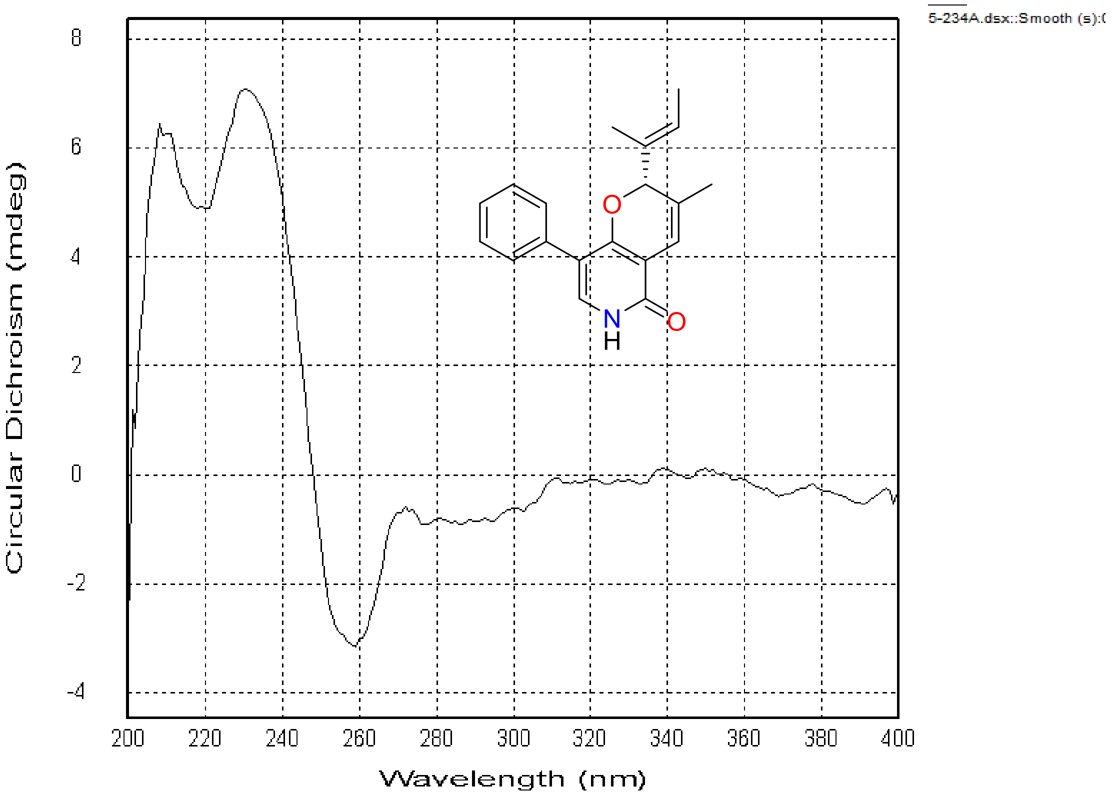

Figure S50. CD spectrum of **4a**

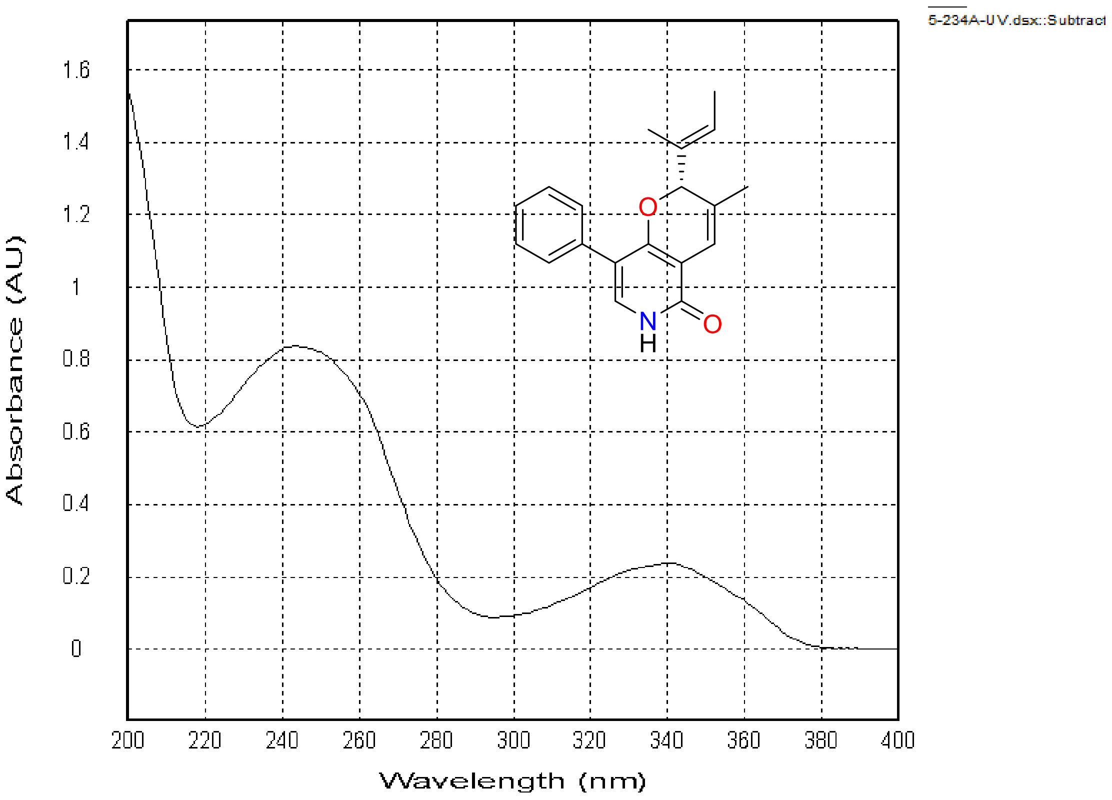

Figure S51. UV spectrum of **4a**

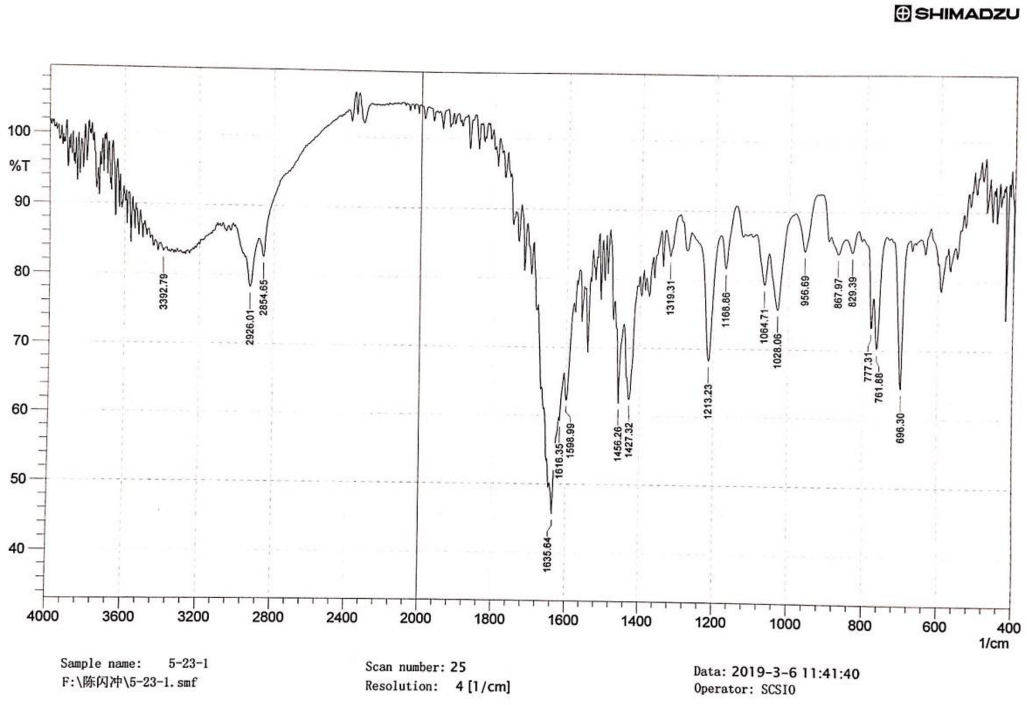

Figure S52. IR spectrum of **4a**

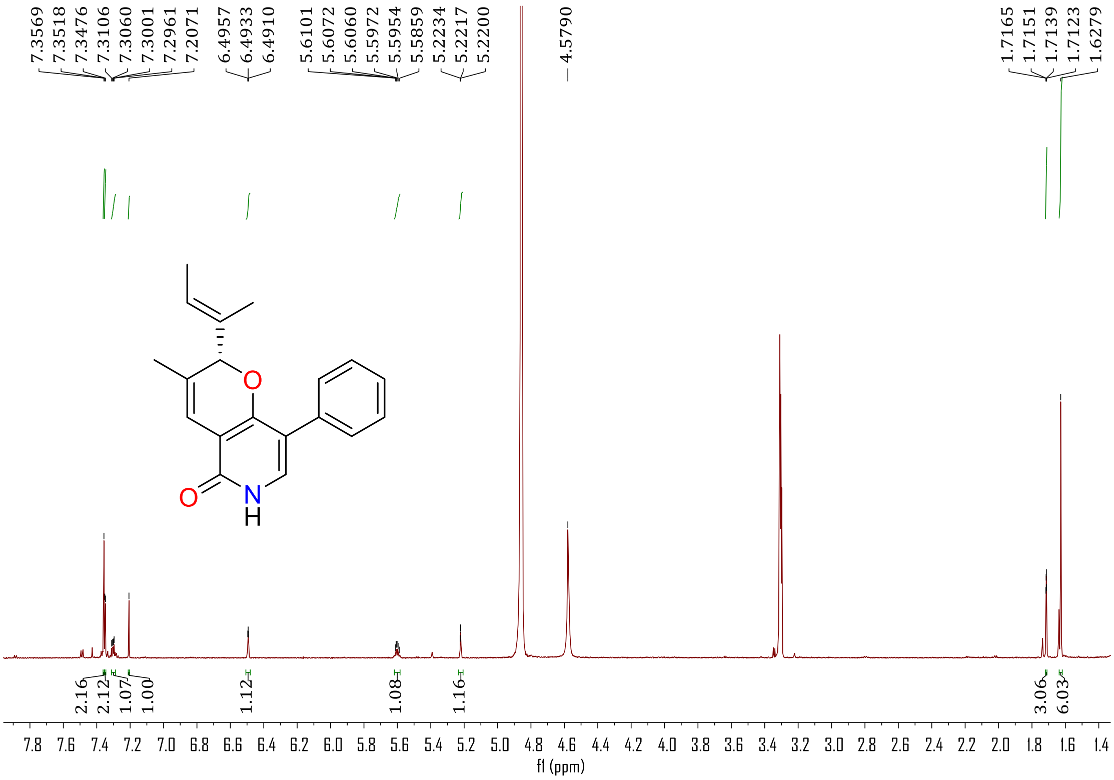

Figure S53. ^{1}H NMR spectrum (600 MHz, CD_3OD) of **4b**

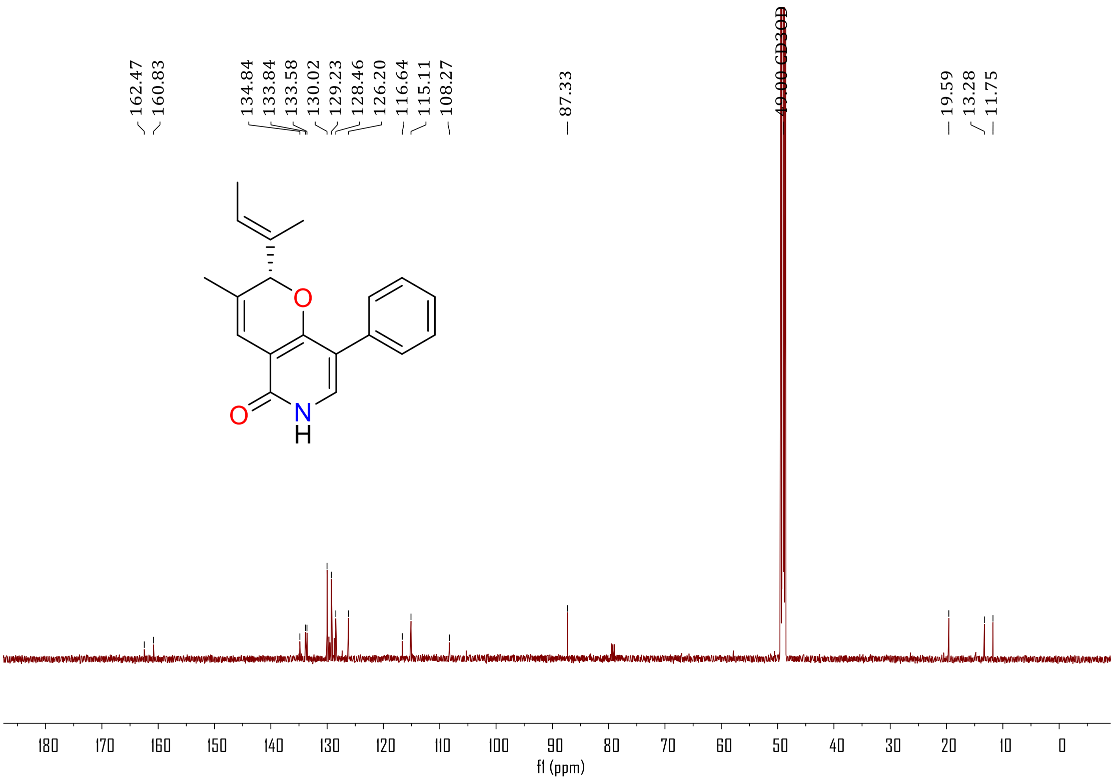

Figure S54. ^{13}C NMR spectrum (150 MHz, CD_3OD) of **4b**

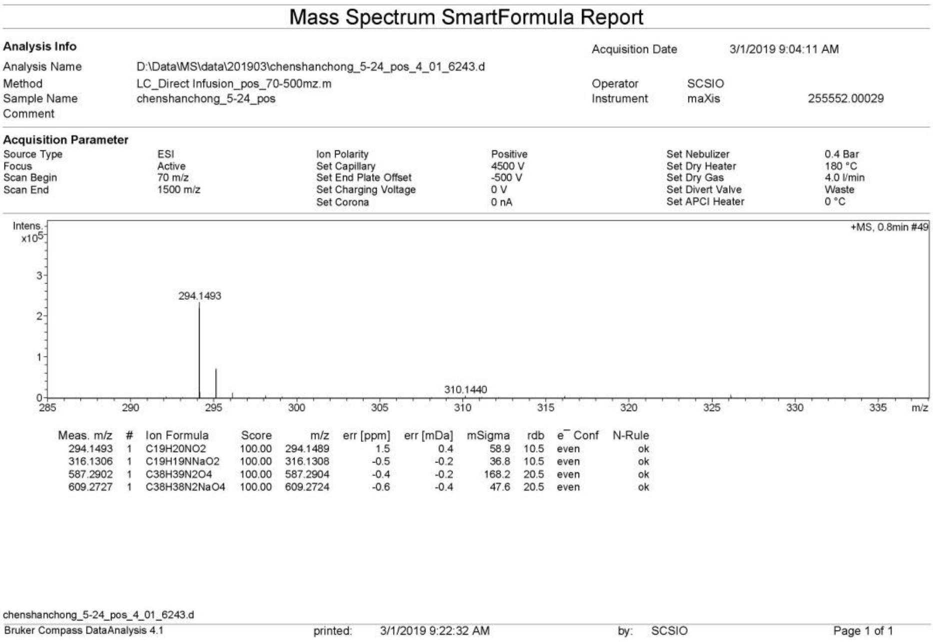

Figure S55. HRESIMS spectrum of **4b**

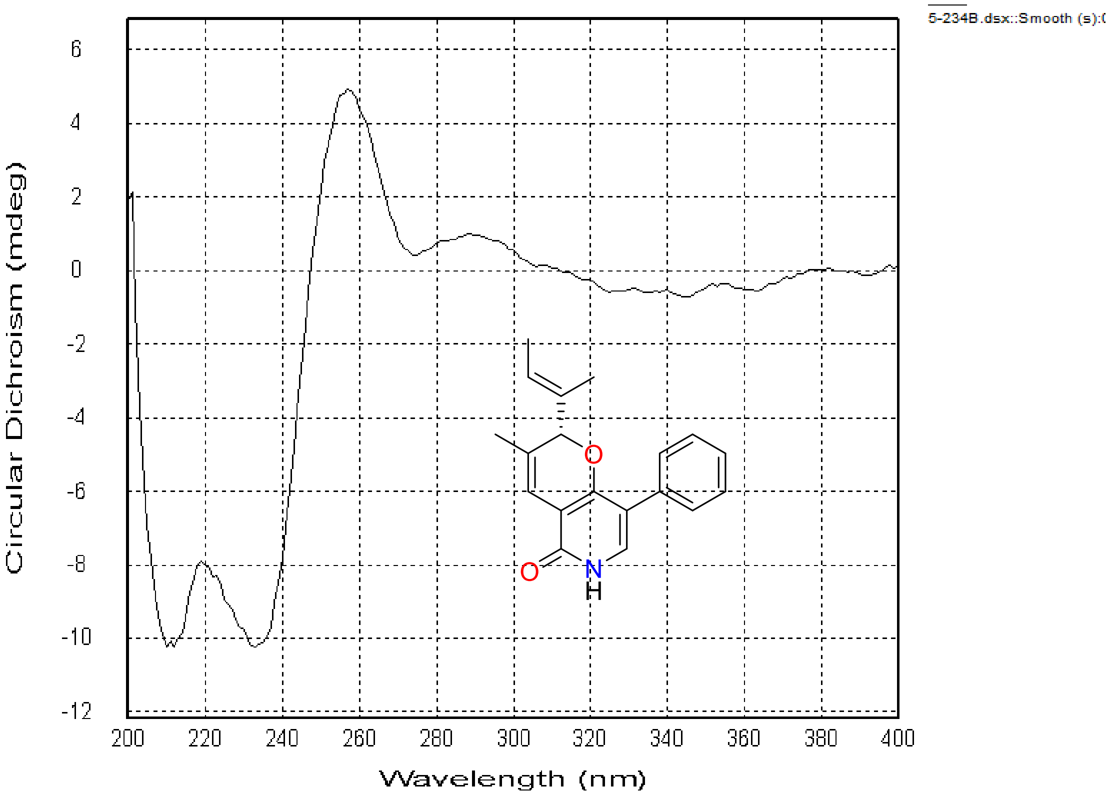

Figure S56. CD spectrum of **4b**

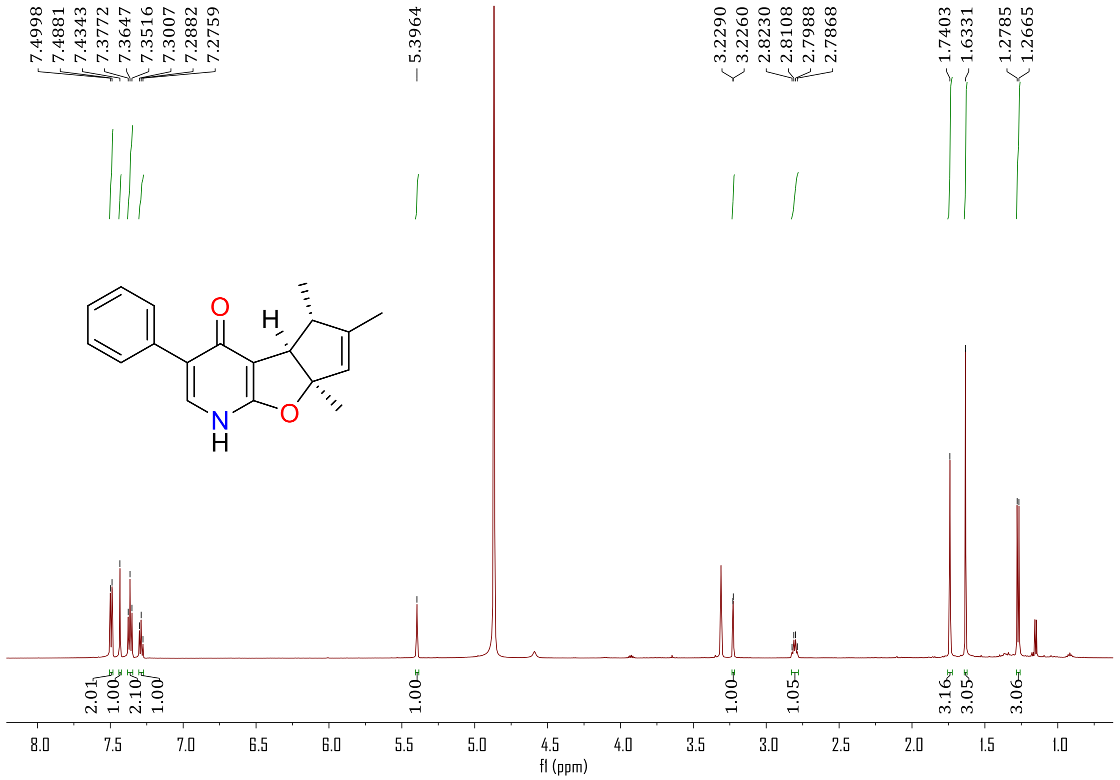

Figure S57. ^{1}H NMR spectrum (600 MHz, CD_3OD) of **5a**

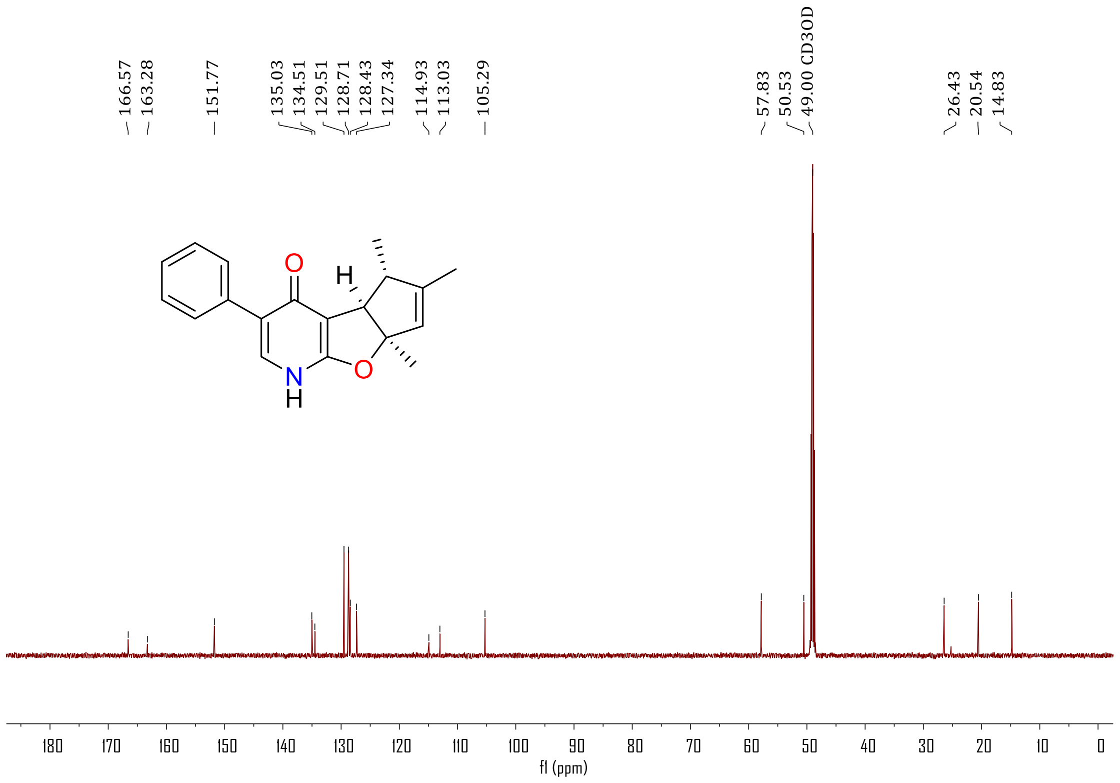

Figure S58. ^{13}C NMR spectrum (150 MHz, CD_3OD) of **5a**

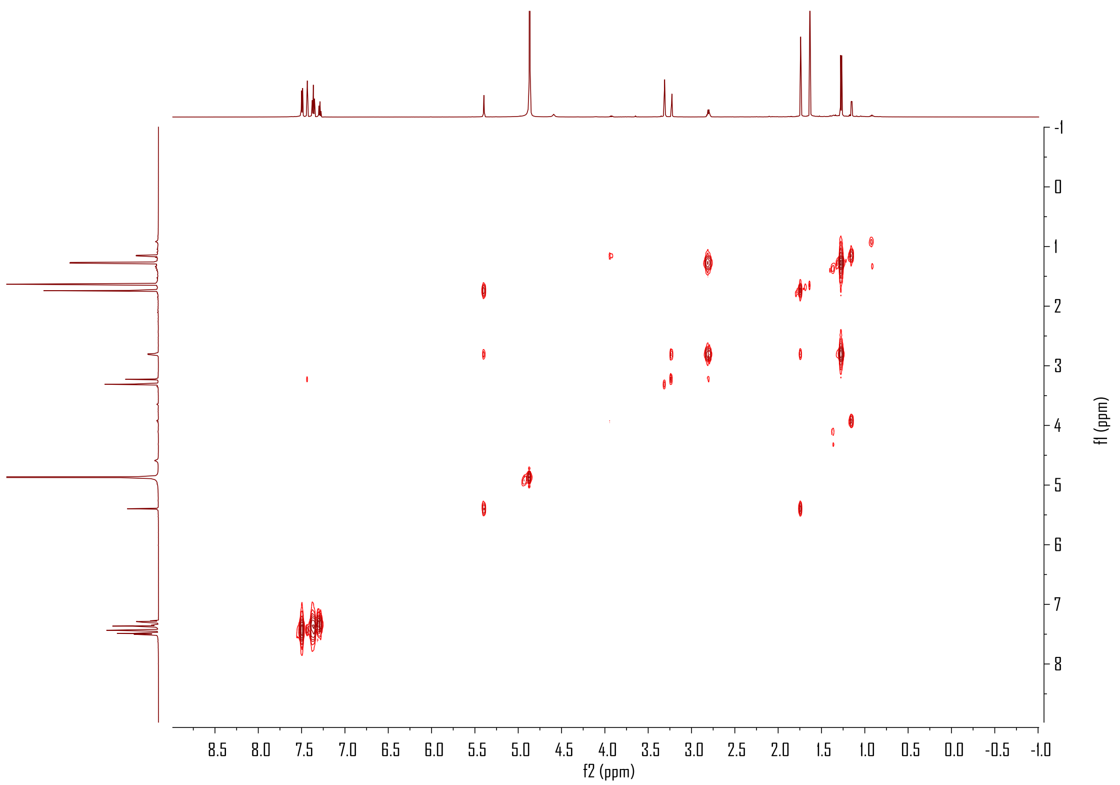

Figure S59. ^1H-^1H COSY spectrum of **5a** in CD_3OD

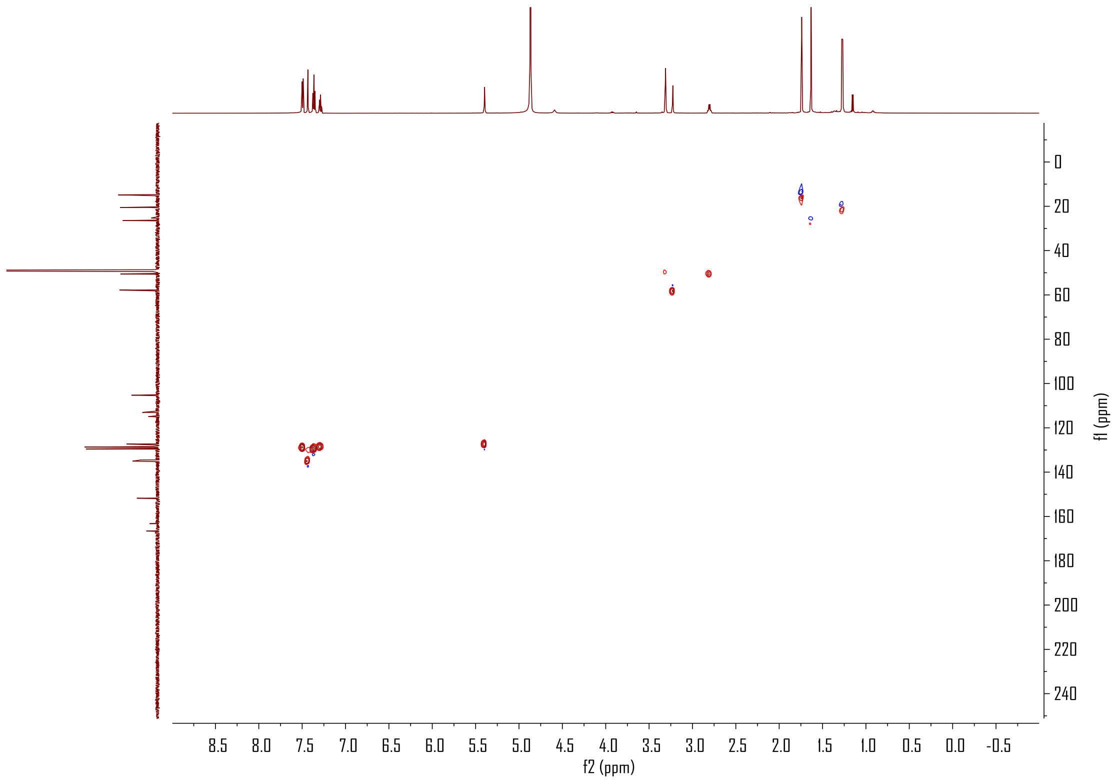

Figure S60. HSQC spectrum of **5a** in CD_3OD

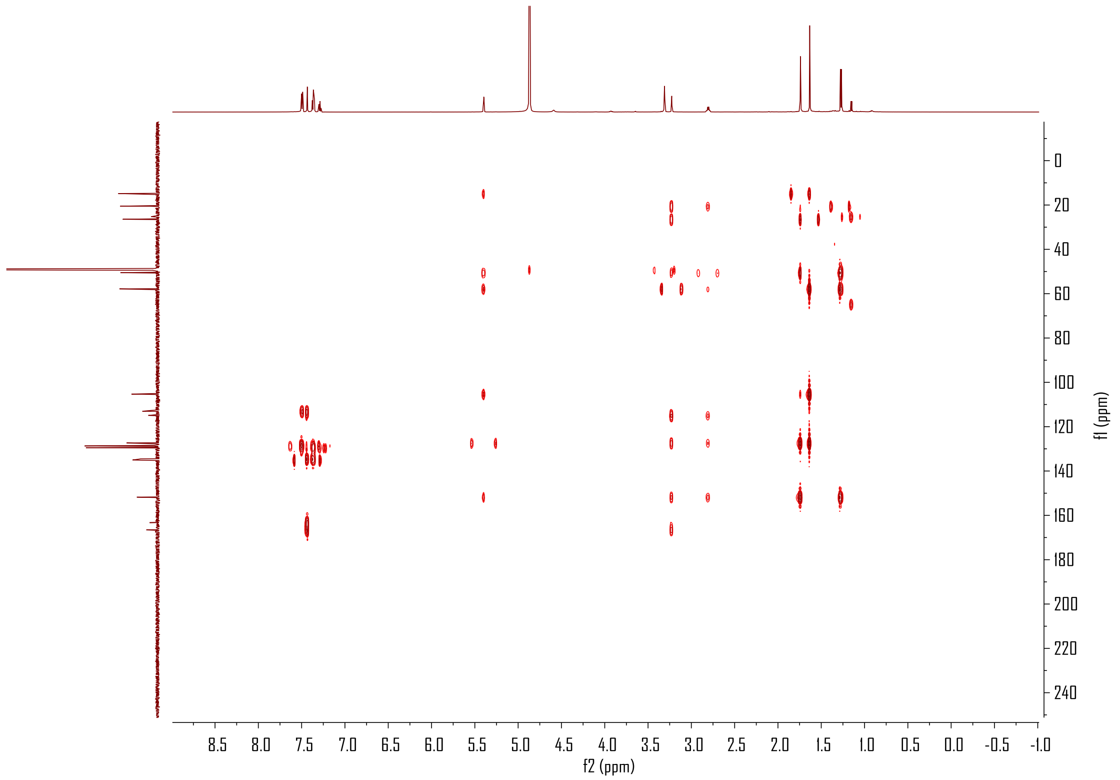

Figure S61. HMBC spectrum of **5a** in CD_3OD

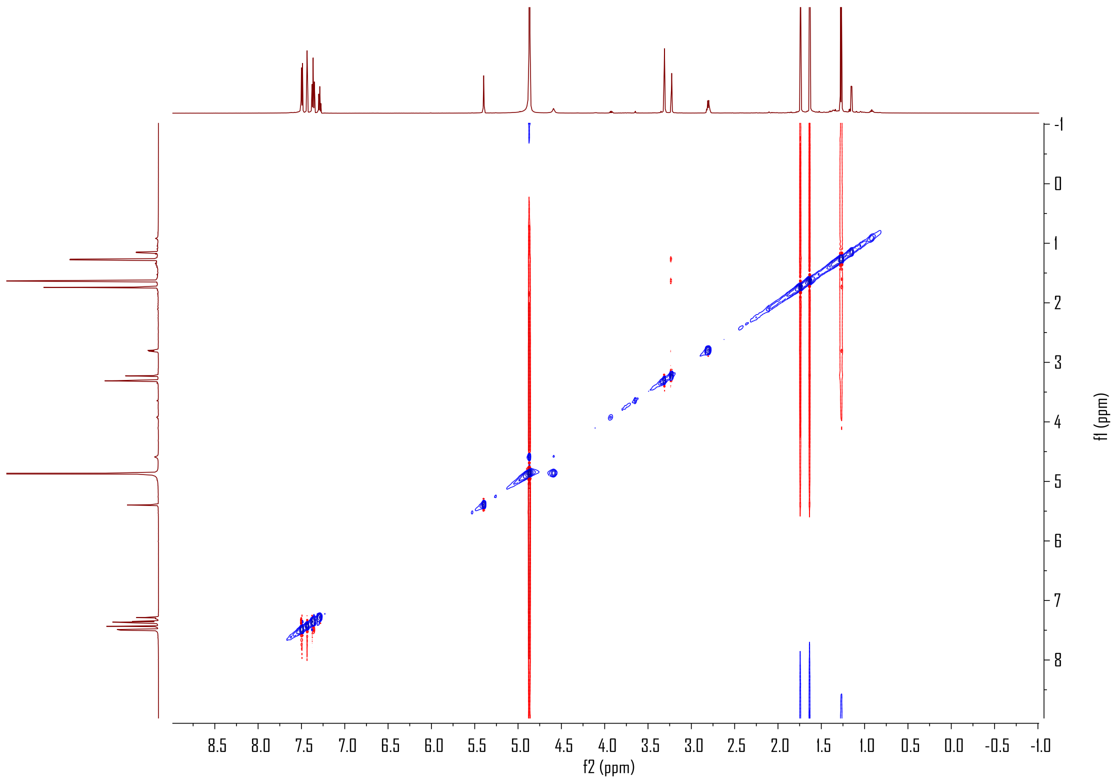

Figure S62. NOESY spectrum of **5a** in CD_3OD

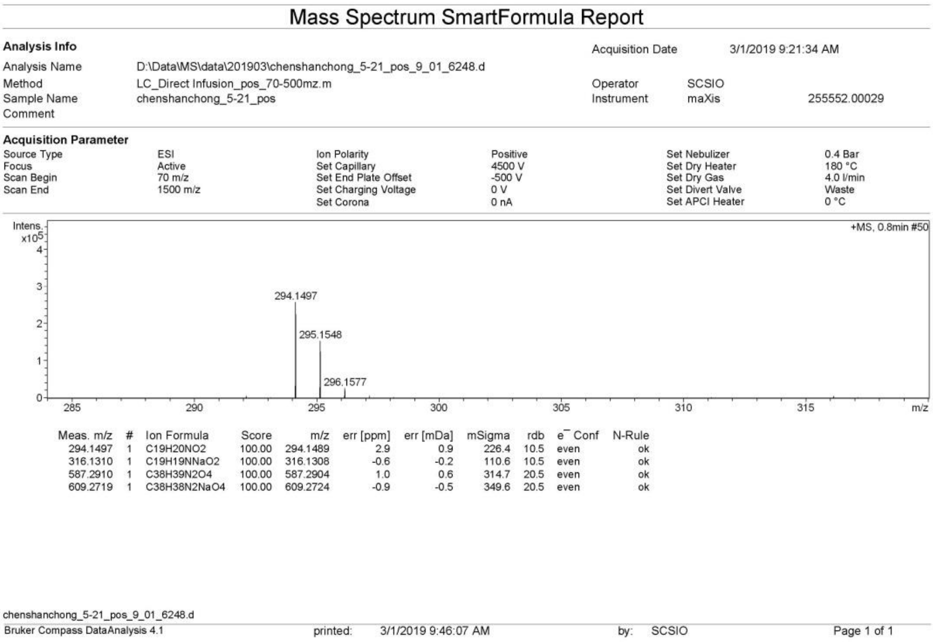
Mass Spectrum SmartFormula Report

Analysis Info
Acquisition Date 3/1/2019 9:21:34 AM
Analysis Name D:\Data\MS\data\201903\chenshanchong_5-21_pos_9_01_6248.d
Method LC_Direct Infusion_pos_70-500mz.m
Operator SCSIO
Sample Name chenshanchong_5-21_pos
Instrument maXis 255552.00029
Comment

Acquisition Parameter

Source Type	ESI	Ion Polarity	Positive	Set Nebulizer	0.4 Bar
Focus	Active	Set Capillary	4500 V	Set Dry Heater	180 °C
Scan Begin	70 m/z	Set End Plate Offset	-500 V	Set Dry Gas	4.0 l/min
Scan End	1500 m/z	Set Charging Voltage	0 V	Set Divert Valve	Waste
		Set Corona	0 nA	Set APCI Heater	0 °C

Meas. m/z	#	Ion Formula	Score	m/z	err [ppm]	err [mDa]	mSigma	rdb	e⁻ Conf	N-Rule
294.1497	1	C19H20NO2	100.00	294.1489	2.9	0.9	226.4	10.5	even	ok
316.1310	1	C19H19NNaO2	100.00	316.1308	-0.6	-0.2	110.6	10.5	even	ok
587.2910	1	C38H39N2O4	100.00	587.2904	1.0	0.6	314.7	20.5	even	ok
609.2719	1	C38H38N2NaO4	100.00	609.2724	-0.9	-0.5	349.6	20.5	even	ok

chenshanchong_5-21_pos_9_01_6248.d
Bruker Compass DataAnalysis 4.1 printed: 3/1/2019 9:46:07 AM by: SCSIO Page 1 of 1

Figure S63. HRESIMS spectrum of **5a**

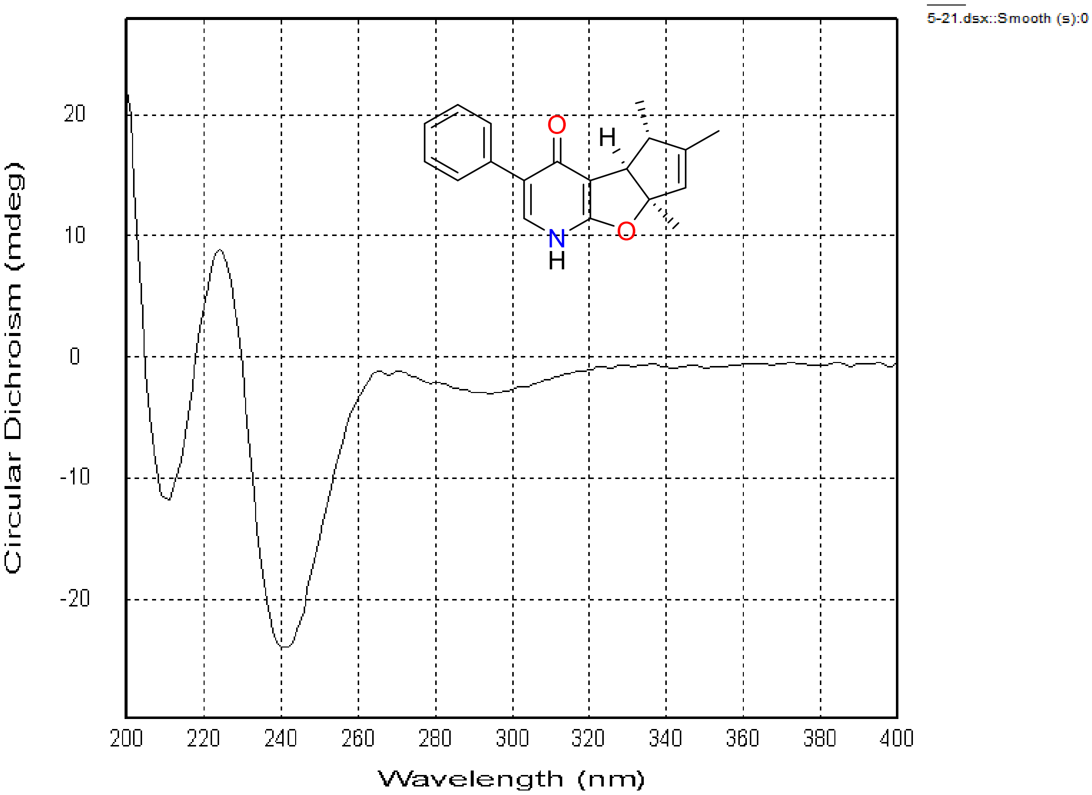

Figure S64. CD spectrum of **5a**

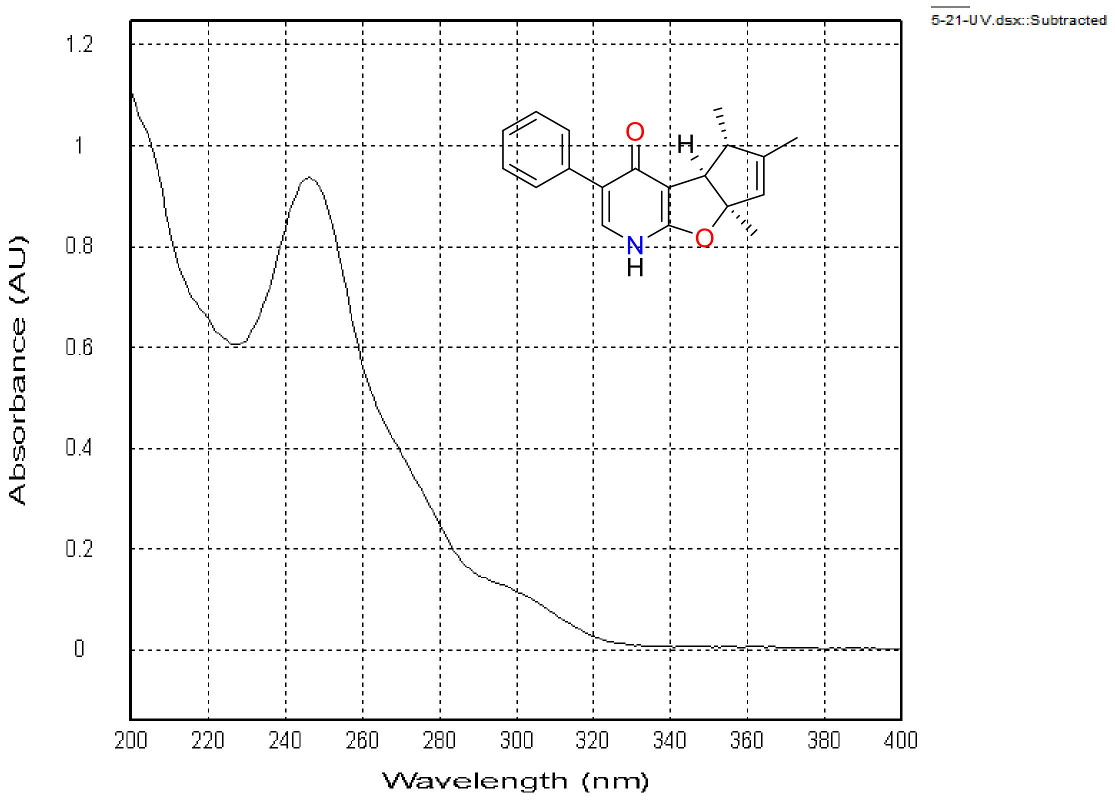

Figure S65. UV spectrum of **5a**

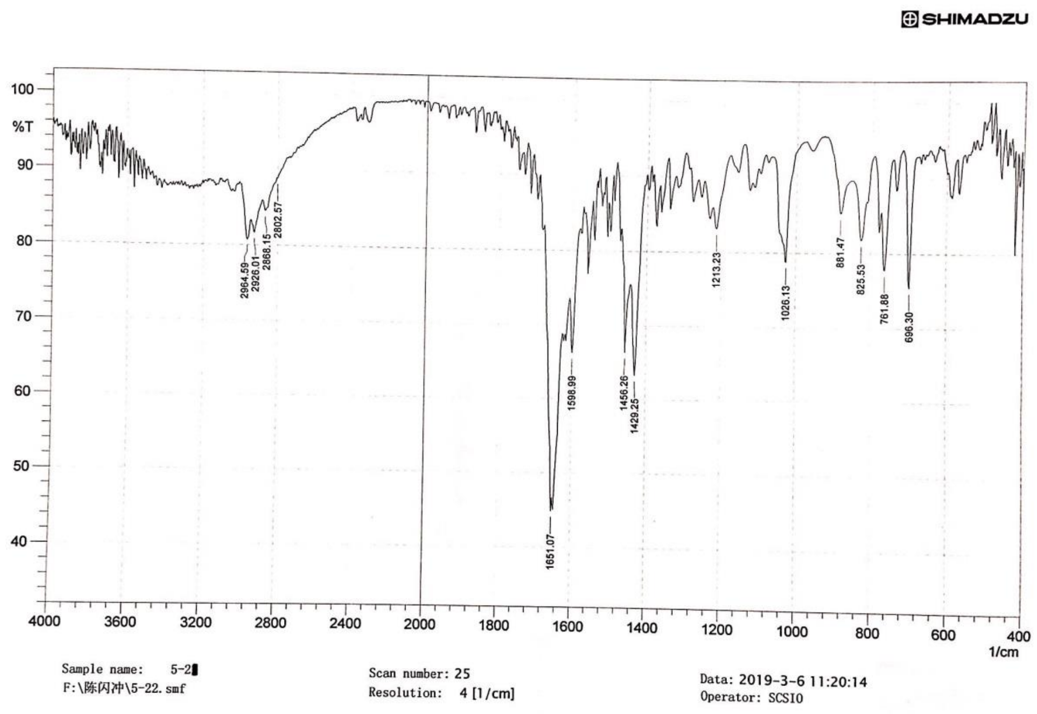

Figure S66. IR spectrum of **5a**

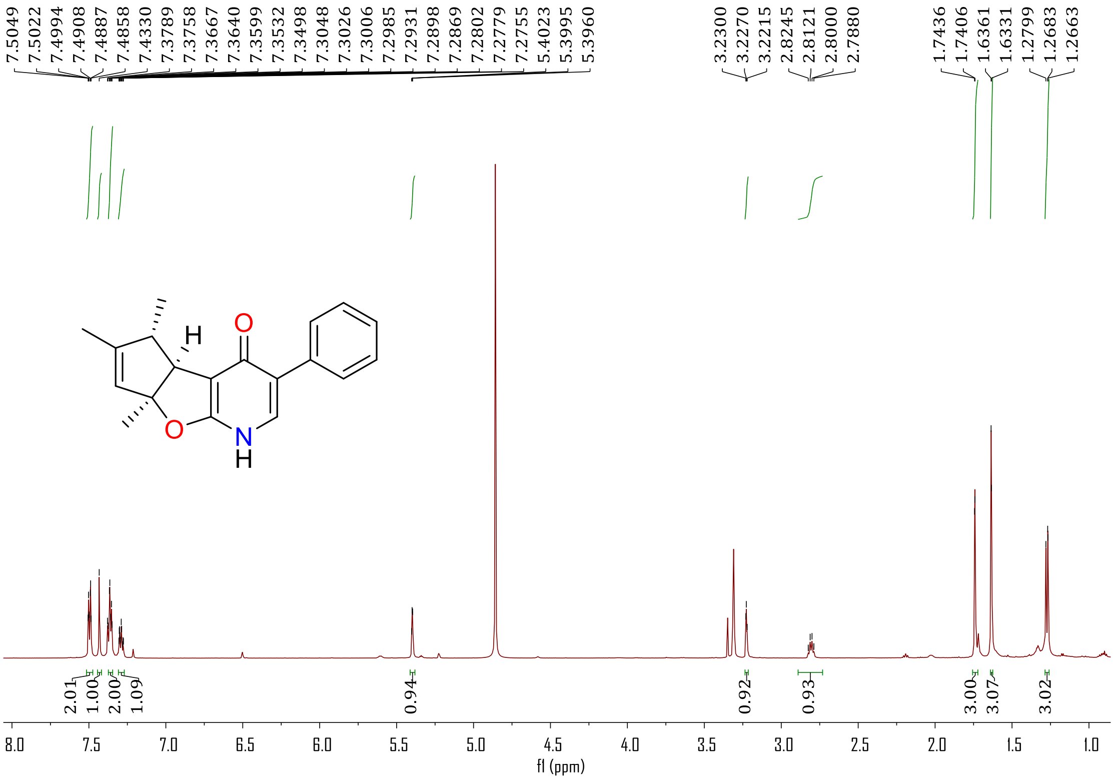

Figure S67. ^{1}H NMR spectrum (600 MHz, CD_3OD) of **5b**

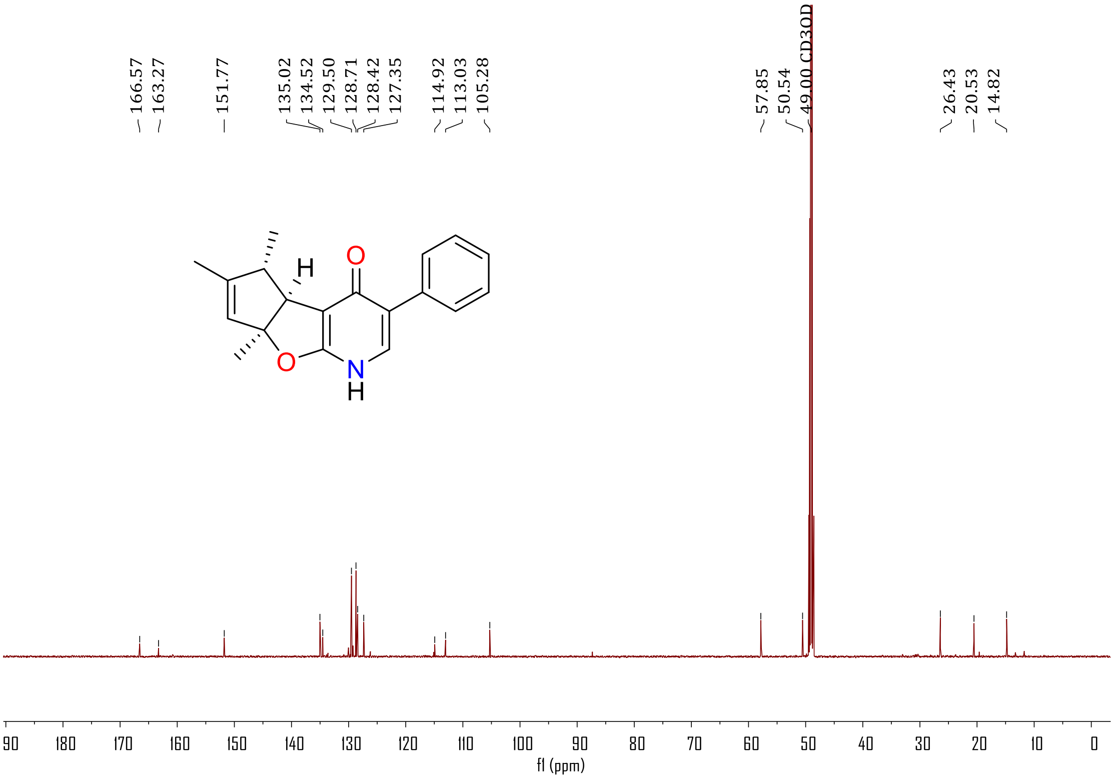

Figure S68. ^{13}C NMR spectrum (150 MHz, CD_3OD) of **5b**

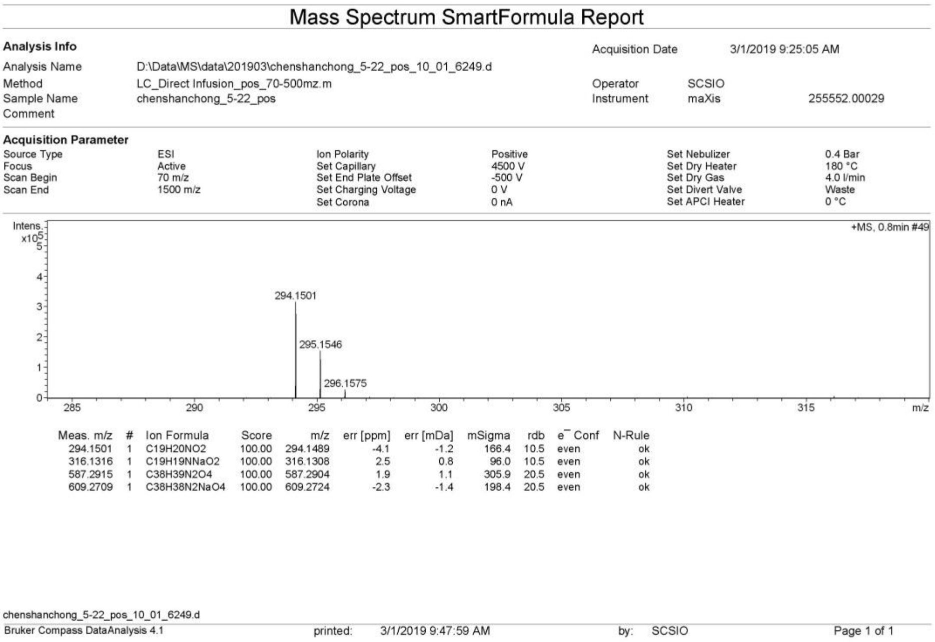

Mass Spectrum SmartFormula Report

Analysis Info

Acquisition Date 3/1/2019 9:25:05 AM

Analysis Name D:\Data\MS\data\201903\chenshanchong_5-22_pos_10_01_6249.d

Method LC_Direct Infusion_pos_70-500mz.m

Operator SCSIO

Sample Name chenshanchong_5-22_pos

Instrument maXis 255552.00029

Comment

Acquisition Parameter

Source Type	ESI	Ion Polarity	Positive	Set Nebulizer	0.4 Bar
Focus	Active	Set Capillary	4500 V	Set Dry Heater	180 °C
Scan Begin	70 m/z	Set End Plate Offset	-500 V	Set Dry Gas	4.0 l/min
Scan End	1500 m/z	Set Charging Voltage	0 V	Set Divert Valve	Waste
		Set Corona	0 nA	Set APCI Heater	0 °C

Meas. m/z	#	Ion Formula	Score	m/z	err [ppm]	err [mDa]	mSigma	rdb	e⁻ Conf	N-Rule
294.1501	1	C19H20NO2	100.00	294.1489	-4.1	-1.2	166.4	10.5	even	ok
316.1316	1	C19H19NNaO2	100.00	316.1308	2.5	0.8	96.0	10.5	even	ok
587.2915	1	C38H39N2O4	100.00	587.2904	1.9	1.1	305.9	20.5	even	ok
609.2709	1	C38H38N2NaO4	100.00	609.2724	-2.3	-1.4	198.4	20.5	even	ok

chenshanchong_5-22_pos_10_01_6249.d

Bruker Compass DataAnalysis 4.1 printed: 3/1/2019 9:47:59 AM by: SCSIO Page 1 of 1

Figure S69. HRESIMS spectrum of **5b**

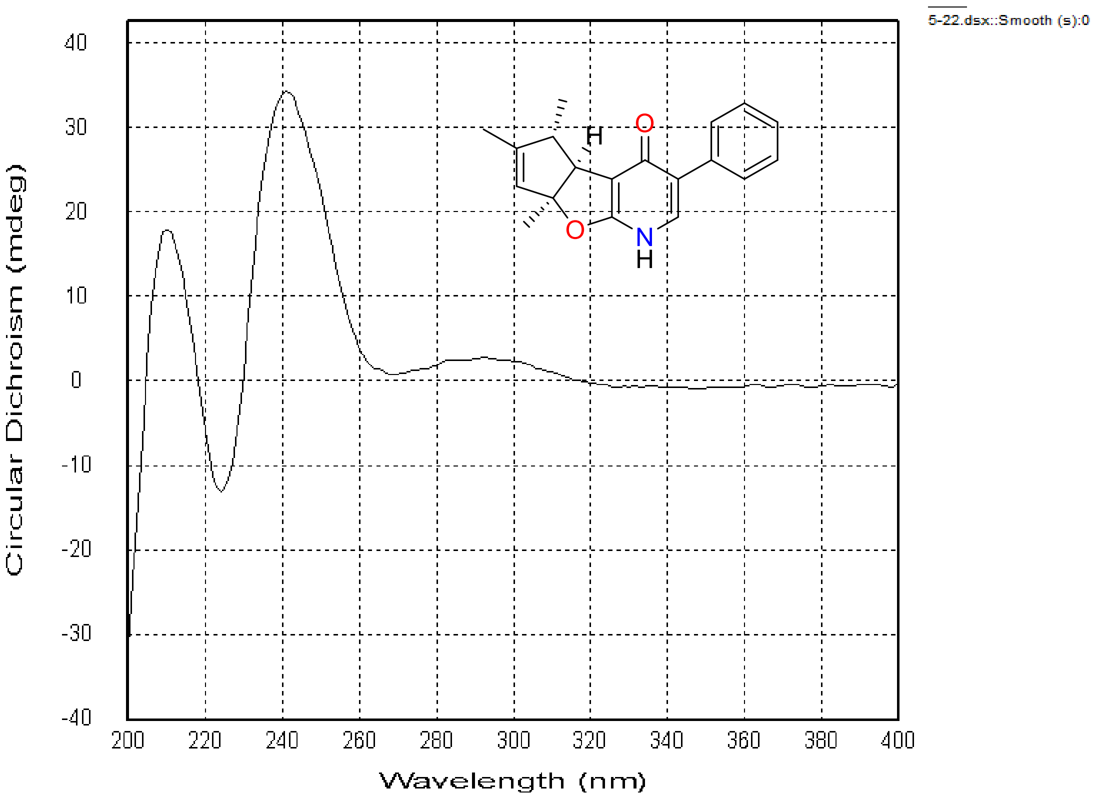

Figure S70. CD spectrum of **5b**

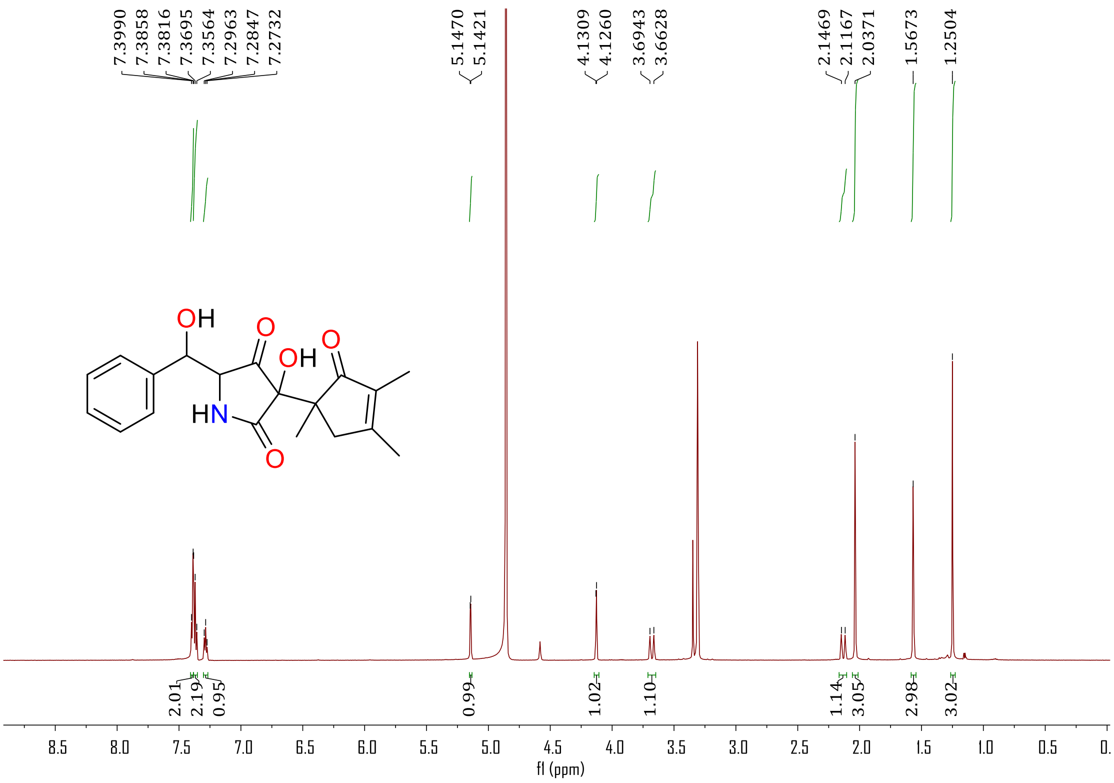

Figure S71. ^{1}H NMR spectrum (600 MHz, CD_3OD) of **6**

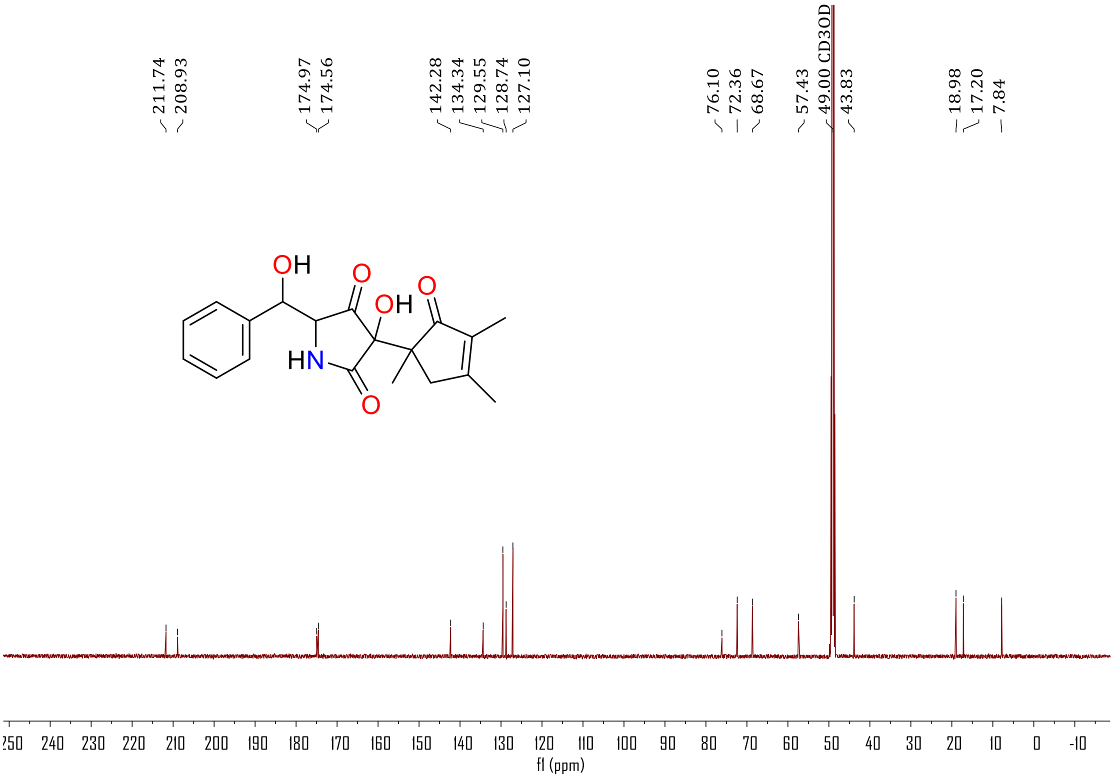

Figure S72. ^{13}C NMR spectrum (150 MHz, CD_3OD) of **6**

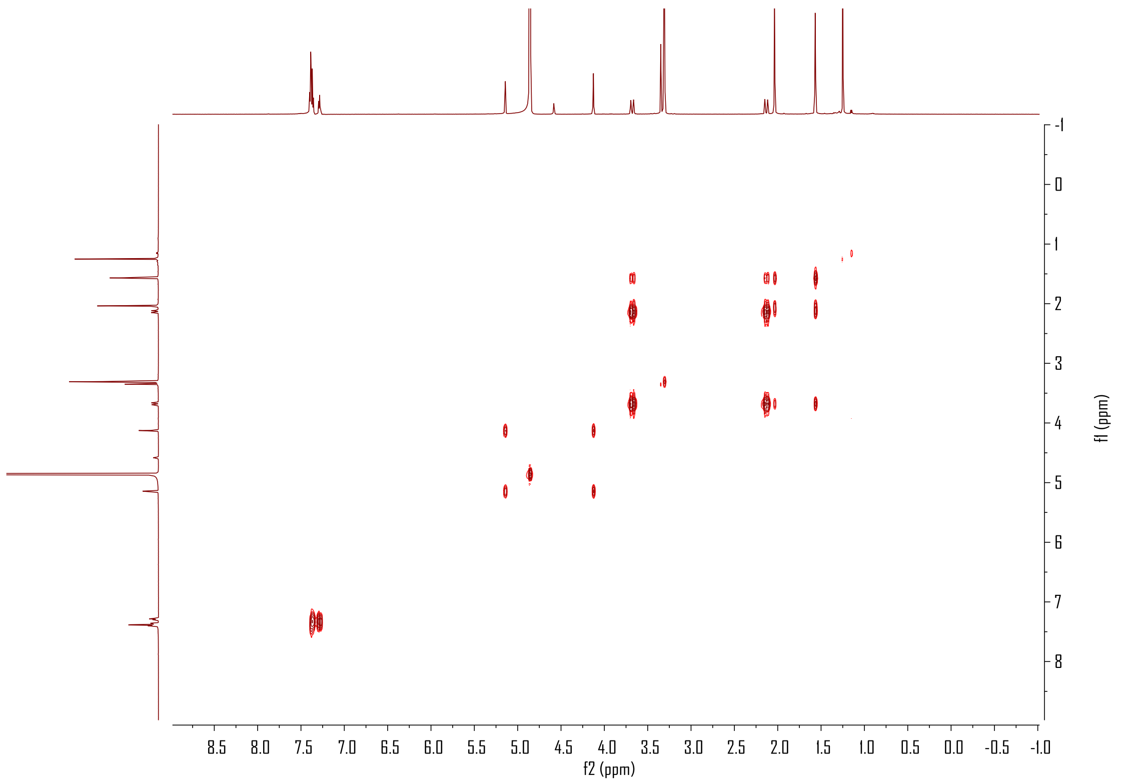

Figure S73. ^{1}H-^{1}H COSY spectrum of **6** in CD_3OD

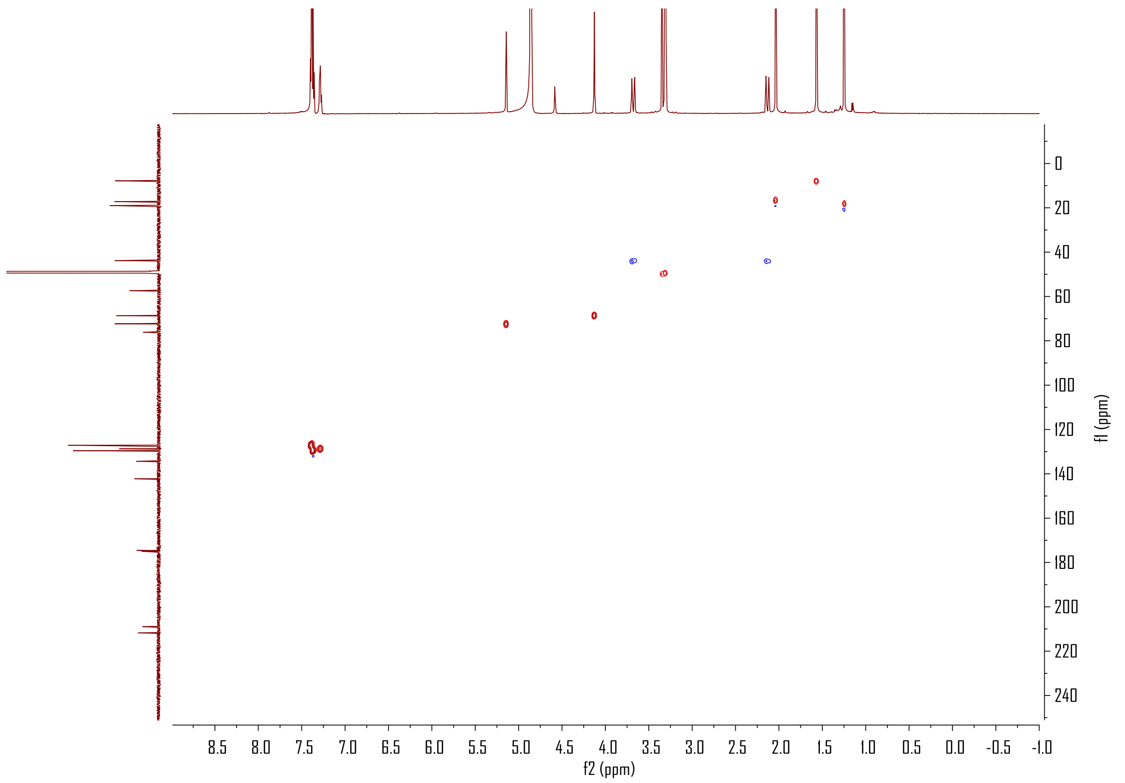

Figure S74. HSQC spectrum of **6** in CD_3OD

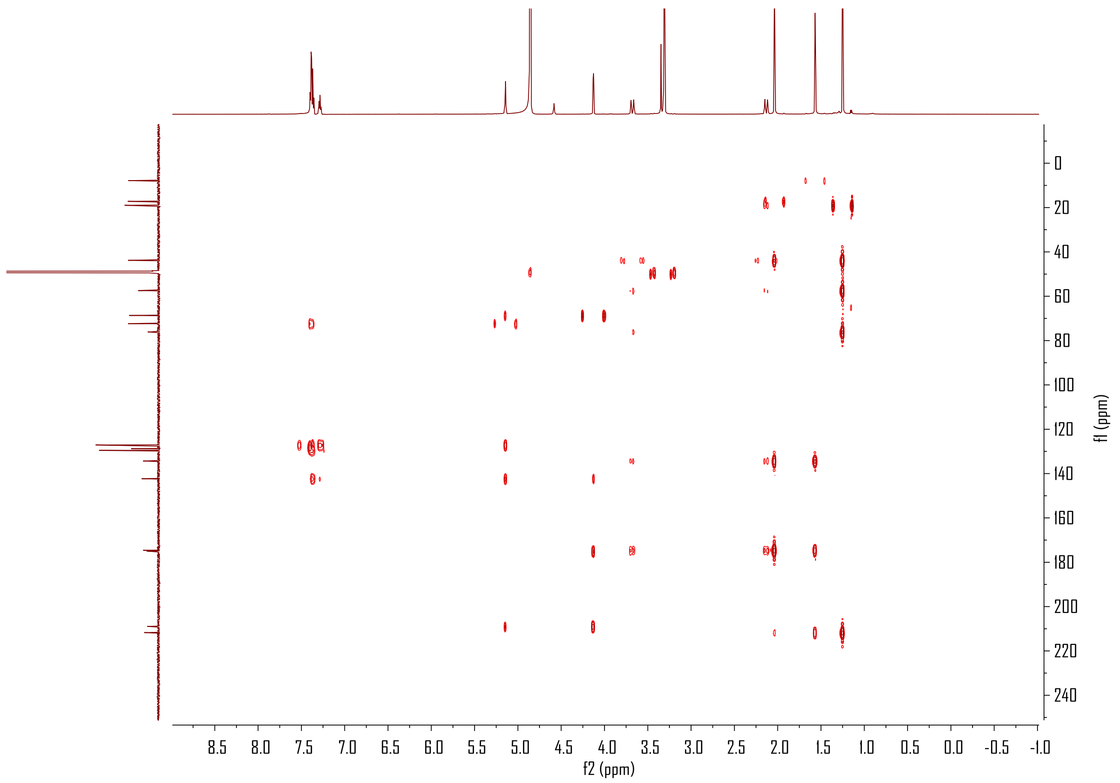

Figure S75. HMBC spectrum of **6** in CD_3OD

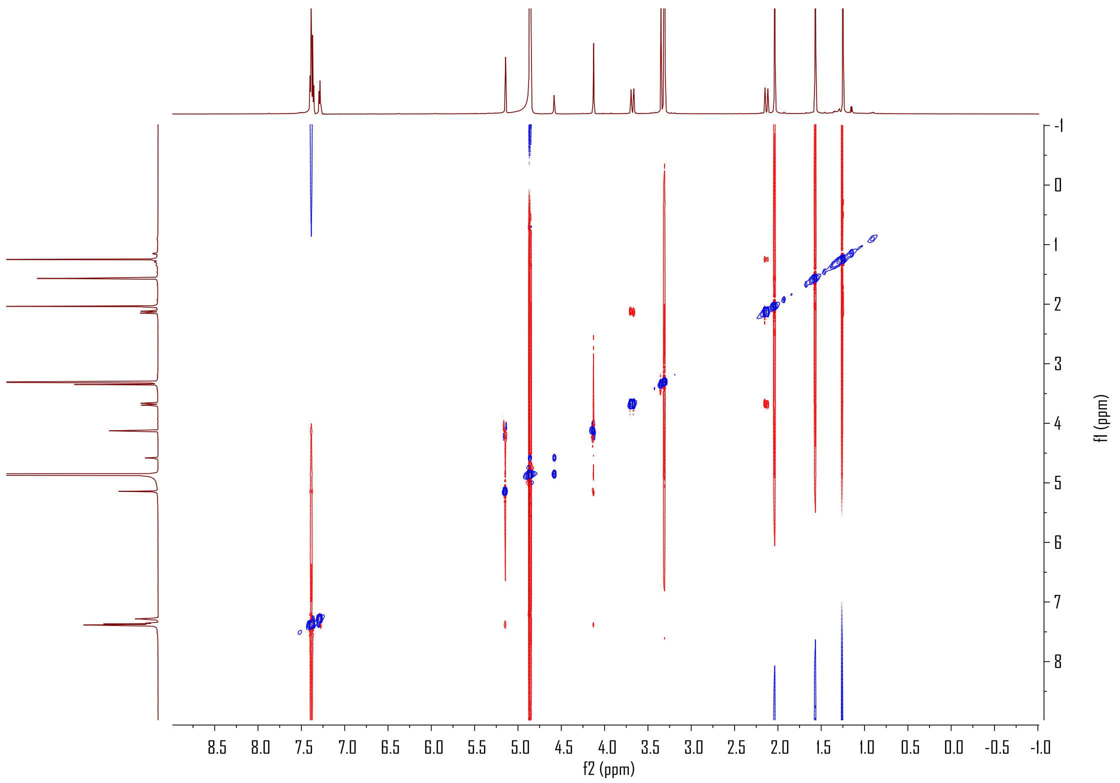

Figure S76. NOESY spectrum of **6** in CD_3OD

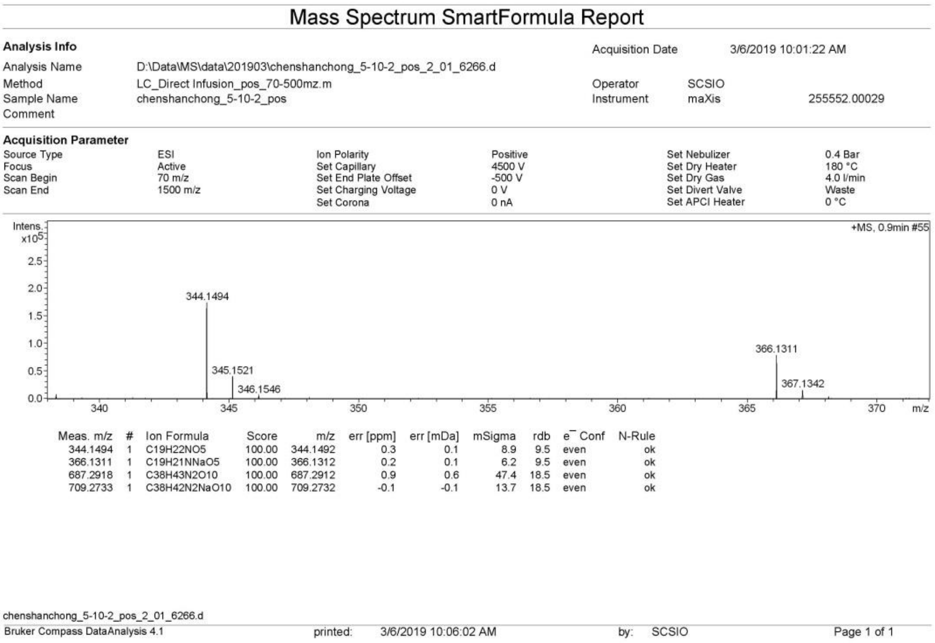

Mass Spectrum SmartFormula Report

Analysis Info

Analysis Name	D:\Data\MS\data\201903\chenshanchong_5-10-2_pos_2_01_6266.d	Acquisition Date	3/6/2019 10:01:22 AM
Method	LC_Direct Infusion_pos_70-500mz.m	Operator	SCSIO
Sample Name	chenshanchong_5-10-2_pos	Instrument	maXis 255552.00029
Comment			

Acquisition Parameter

Source Type	ESI	Ion Polarity	Positive	Set Nebulizer	0.4 Bar
Focus	Active	Set Capillary	4500 V	Set Dry Heater	180 °C
Scan Begin	70 m/z	Set End Plate Offset	-500 V	Set Dry Gas	4.0 l/min
Scan End	1500 m/z	Set Charging Voltage	0 V	Set Divert Valve	Waste
		Set Corona	0 nA	Set APCI Heater	0 °C

Meas. m/z	#	Ion Formula	Score	m/z	err [ppm]	err [mDa]	mSigma	rdb	e¯ Conf	N-Rule
344.1494	1	C19H22NO5	100.00	344.1492	0.3	0.1	8.9	9.5	even	ok
366.1311	1	C19H21NNaO5	100.00	366.1312	0.2	0.1	6.2	9.5	even	ok
687.2918	1	C38H43N2O10	100.00	687.2912	0.9	0.6	47.4	18.5	even	ok
709.2733	1	C38H42N2NaO10	100.00	709.2732	-0.1	-0.1	13.7	18.5	even	ok

chenshanchong_5-10-2_pos_2_01_6266.d
Bruker Compass DataAnalysis 4.1 printed: 3/6/2019 10:06:02 AM by: SCSIO Page 1 of 1

Figure S77. HRESIMS spectrum of **6**

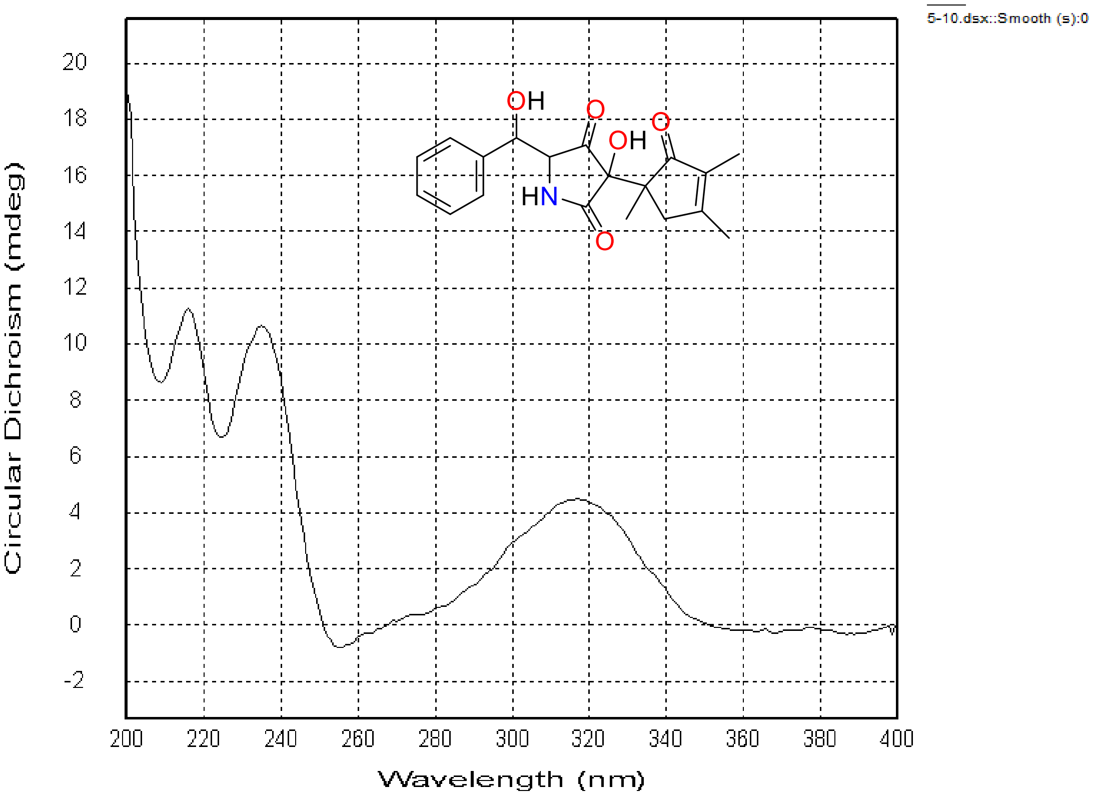

Figure S78. CD spectrum of **6**

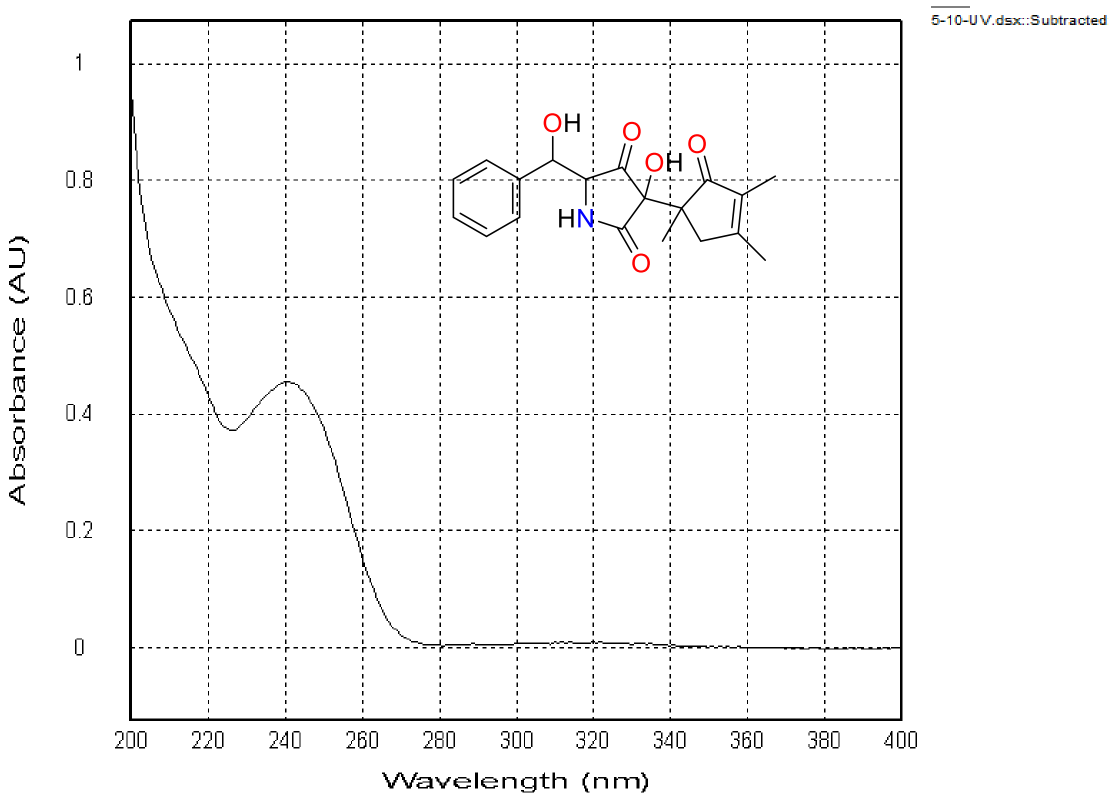

Figure S79. UV spectrum of **6**

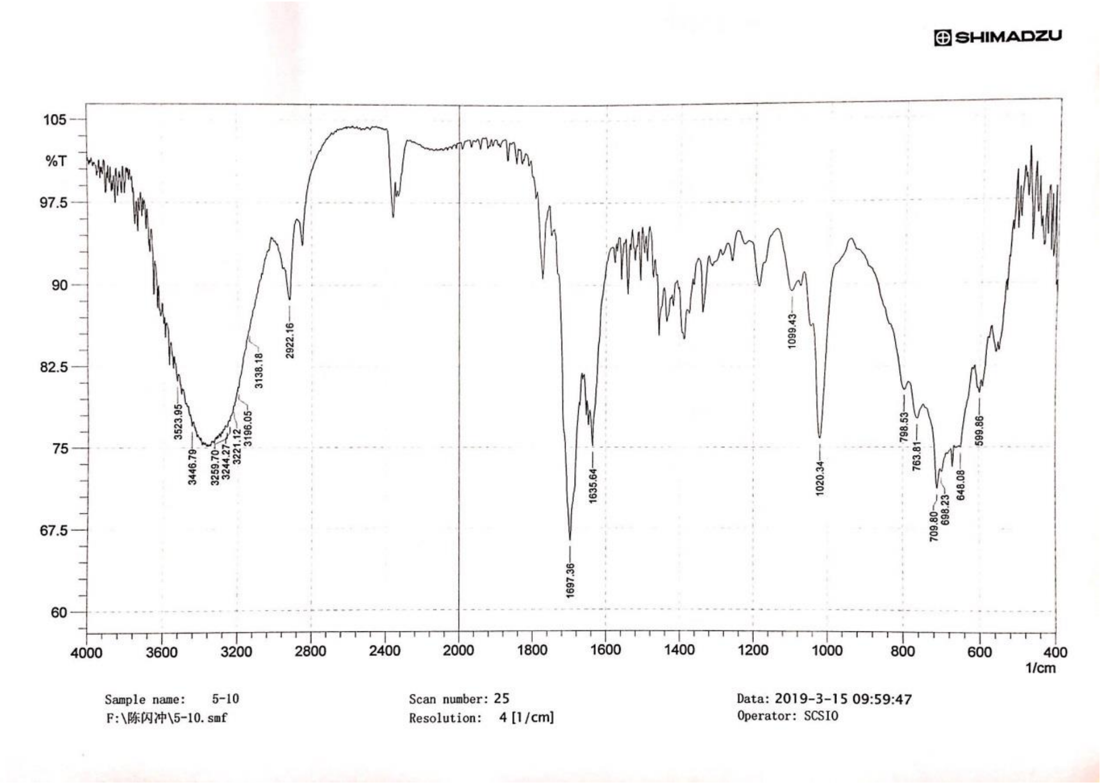

Figure S80. IR spectrum of **6**

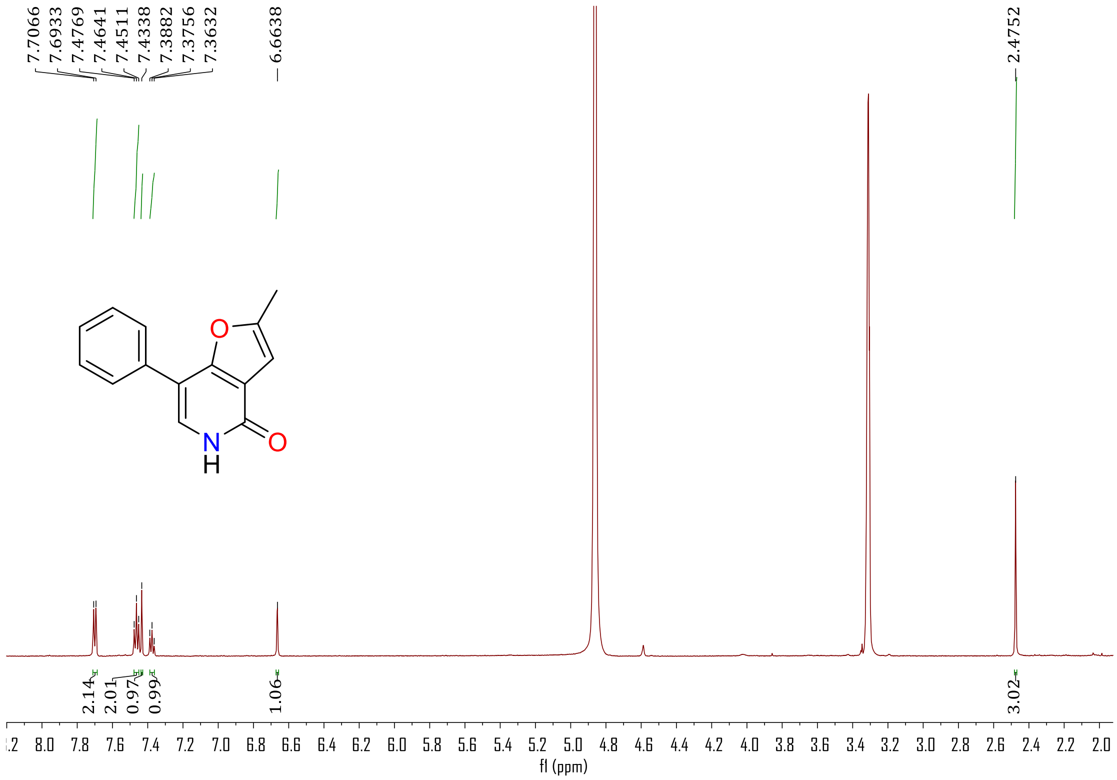

Figure S81. ^{1}H NMR spectrum (600 MHz, CD_3OD) of **7**

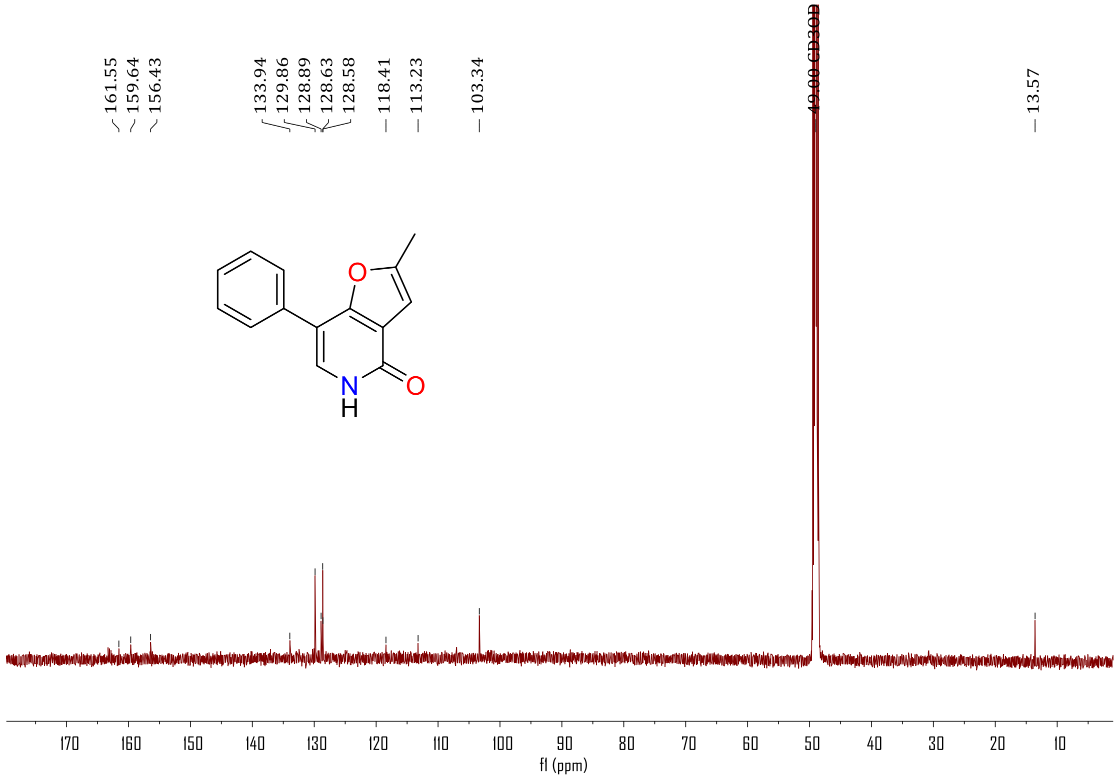

Figure S82. ^{13}C NMR spectrum (150 MHz, CD_3OD) of **7**

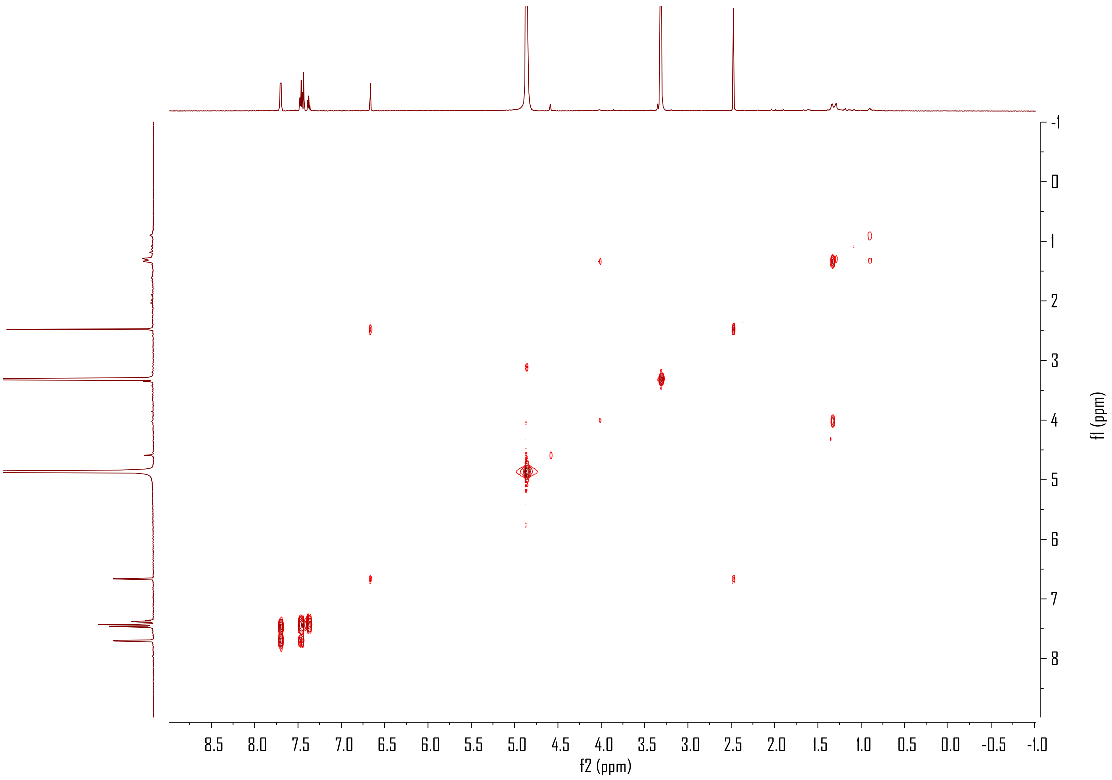

Figure S83. ^{1}H-^{1}H COSY spectrum of **7** in CD_3OD

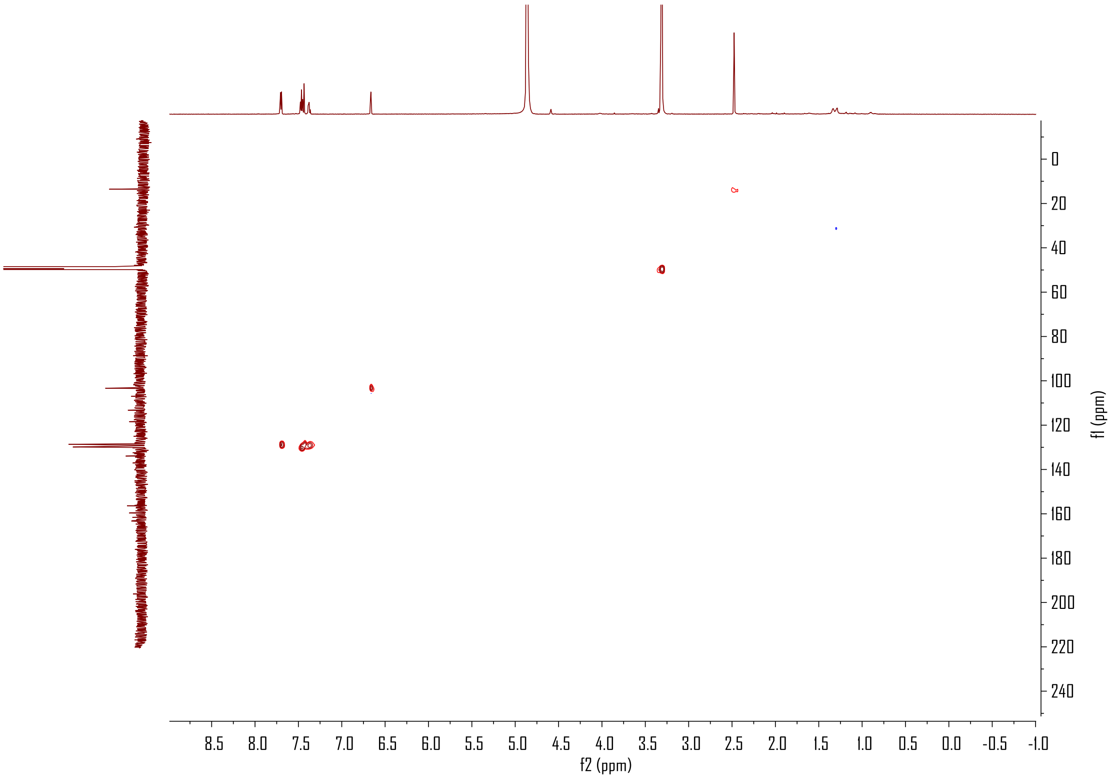

Figure S84. HSQC spectrum of **7** in CD_3OD

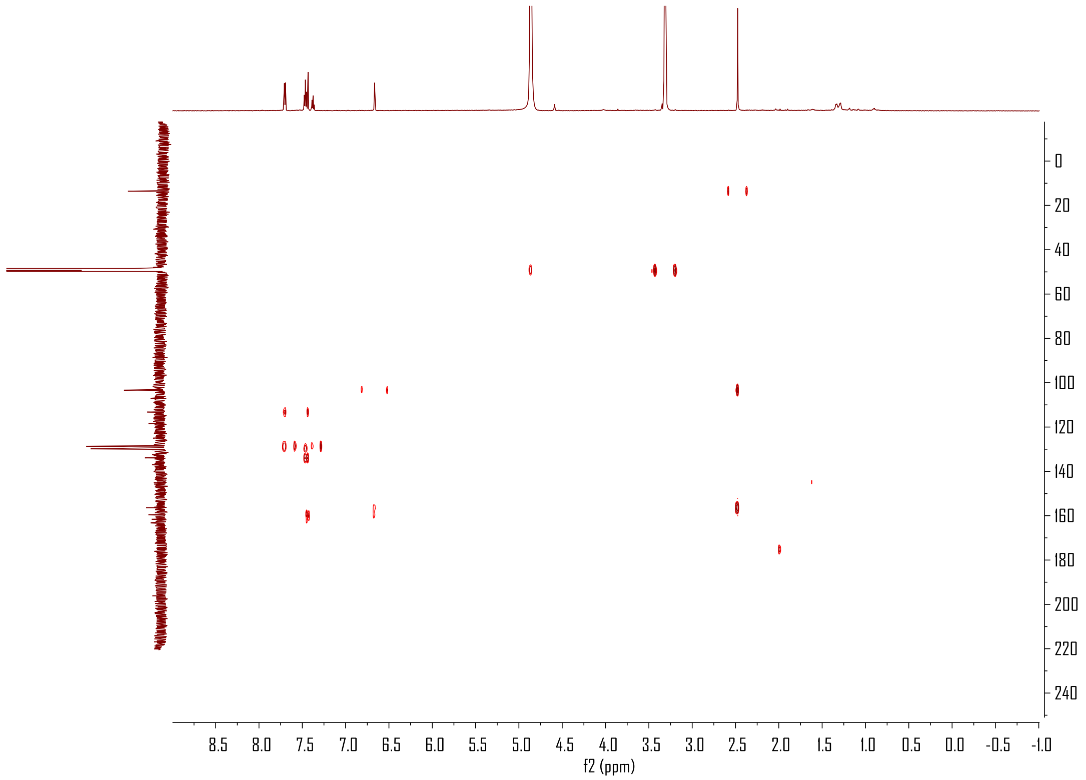

Figure S85. HMBC spectrum of **7** in CD_3OD

Mass Spectrum SmartFormula Report

Analysis Info

Acquisition Date 3/15/2019 8:43:59 AM

Analysis Name D:\Data\MS\data\201903\chenshanchong_5-9_pos_1_01_6302.d

Method LC_Direct Infusion_pos_70-500mz.m

Operator SCSIO

Sample Name chenshanchong_5-9_pos

Instrument maXis 255552.00029

Comment

Acquisition Parameter

Source Type	ESI	Ion Polarity	Positive	Set Nebulizer	0.4 Bar
Focus	Active	Set Capillary	4500 V	Set Dry Heater	180 °C
Scan Begin	70 m/z	Set End Plate Offset	-500 V	Set Dry Gas	4.0 l/min
Scan End	1500 m/z	Set Charging Voltage	0 V	Set Divert Valve	Waste
		Set Corona	0 nA	Set APCI Heater	0 °C

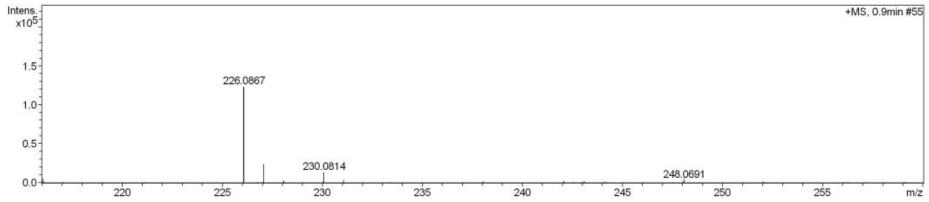

Meas. m/z	#	Ion Formula	Score	m/z	err [ppm]	err [mDa]	mSigma	rdb	e⁻ Conf	N-Rule
226.0867	1	C14H12NO2	100.00	226.0863	-2.0	-0.5	21.7	9.5	even	ok
248.0691	1	C14H11NNaO2	100.00	248.0682	3.6	0.9	91.2	9.5	even	ok
451.1662	1	C28H23N2O4	100.00	451.1652	2.1	1.0	37.6	18.5	even	ok
473.1474	1	C28H22N2NaO4	100.00	473.1472	-0.4	-0.2	44.0	18.5	even	ok

chenshanchong_5-9_pos_1_01_6302.d

Bruker Compass DataAnalysis 4.1 printed: 3/15/2019 8:49:25 AM by: SCSIO Page 1 of 1

Figure S86. HRESIMS spectrum of **7**

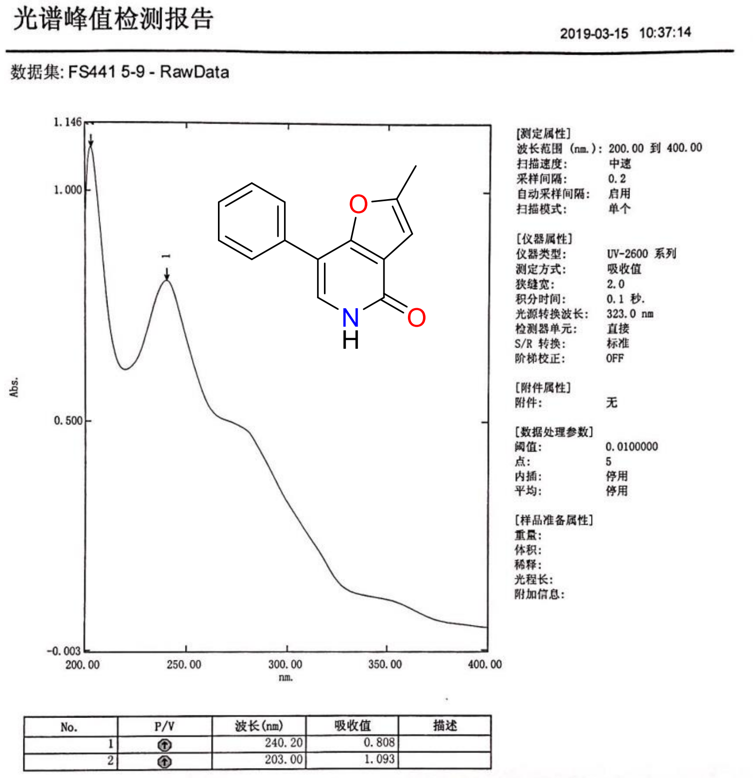

Figure S87. UV spectrum of **7**

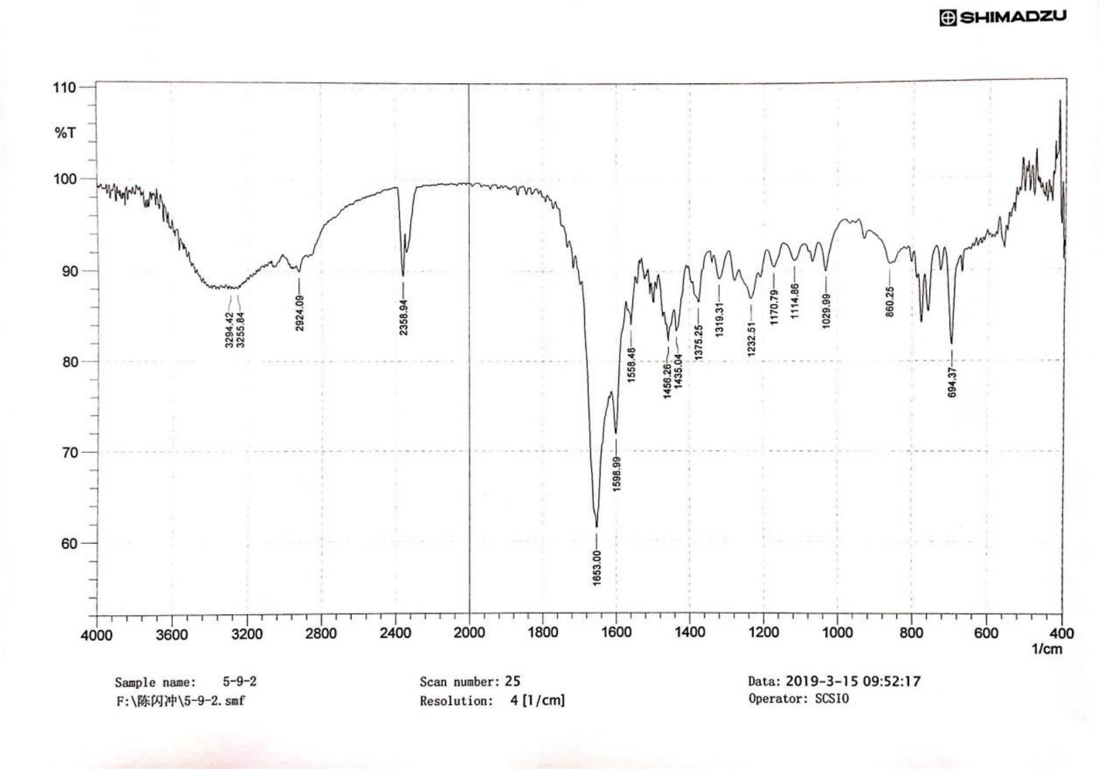

Figure S88. IR spectrum of **7**

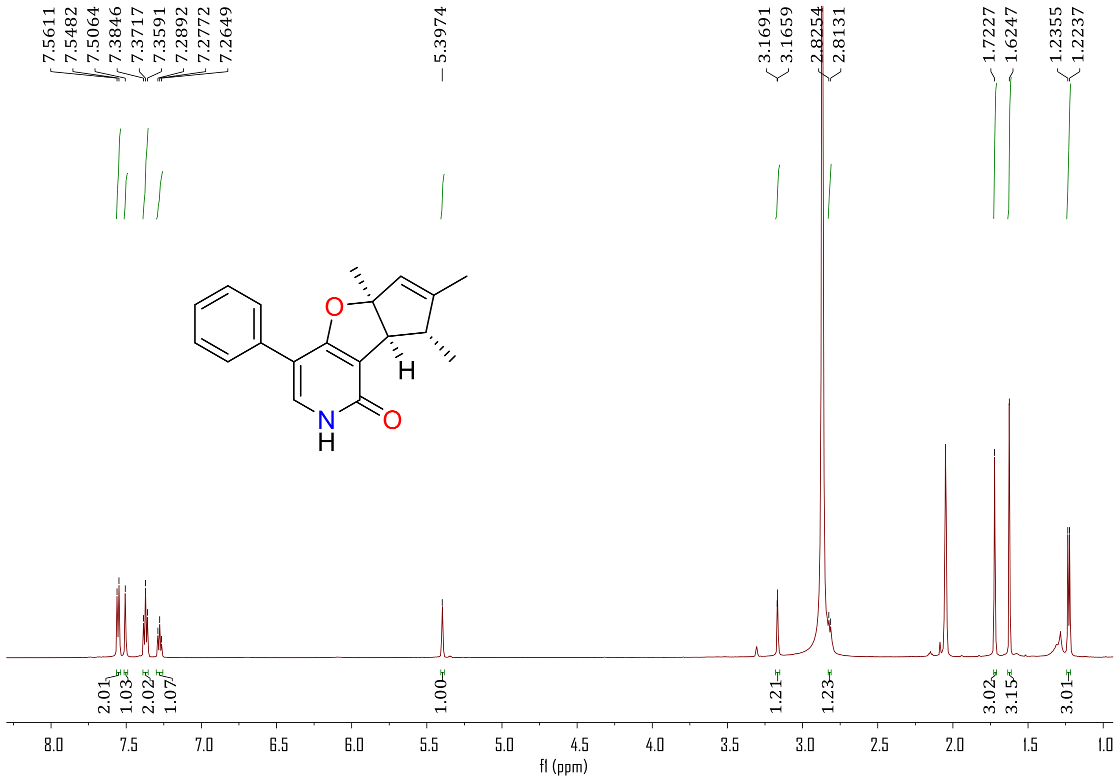

Figure S89. ^{1}H NMR spectrum (600 MHz, CD_3OD) of **8a**

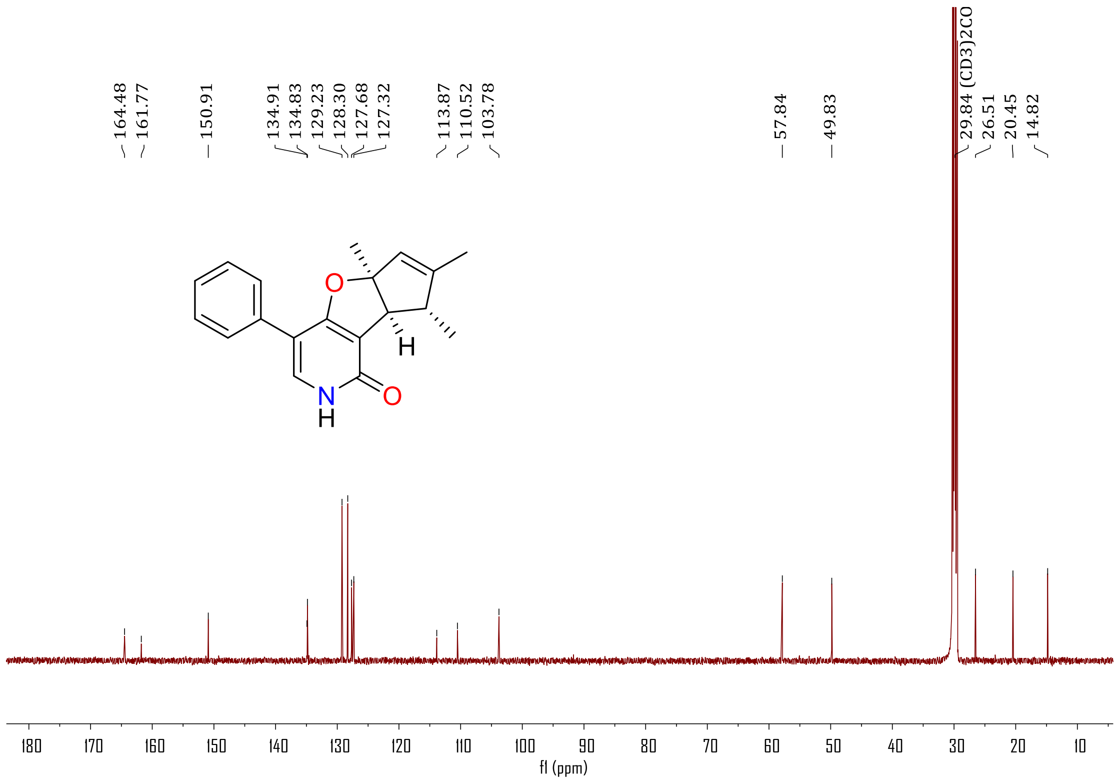

Figure S90. ^{13}C NMR spectrum (150 MHz, CD_3OD) of **8a**

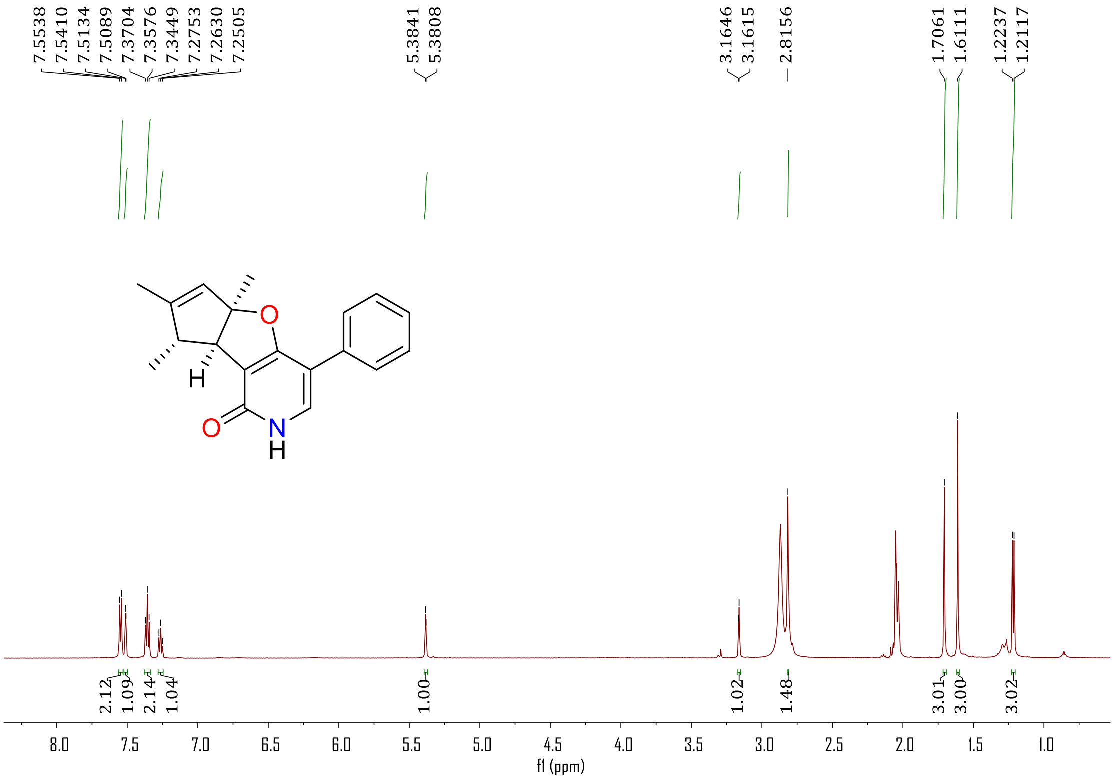

Figure S91. ^{1}H NMR spectrum (600 MHz, CD_3OD) of **8b**

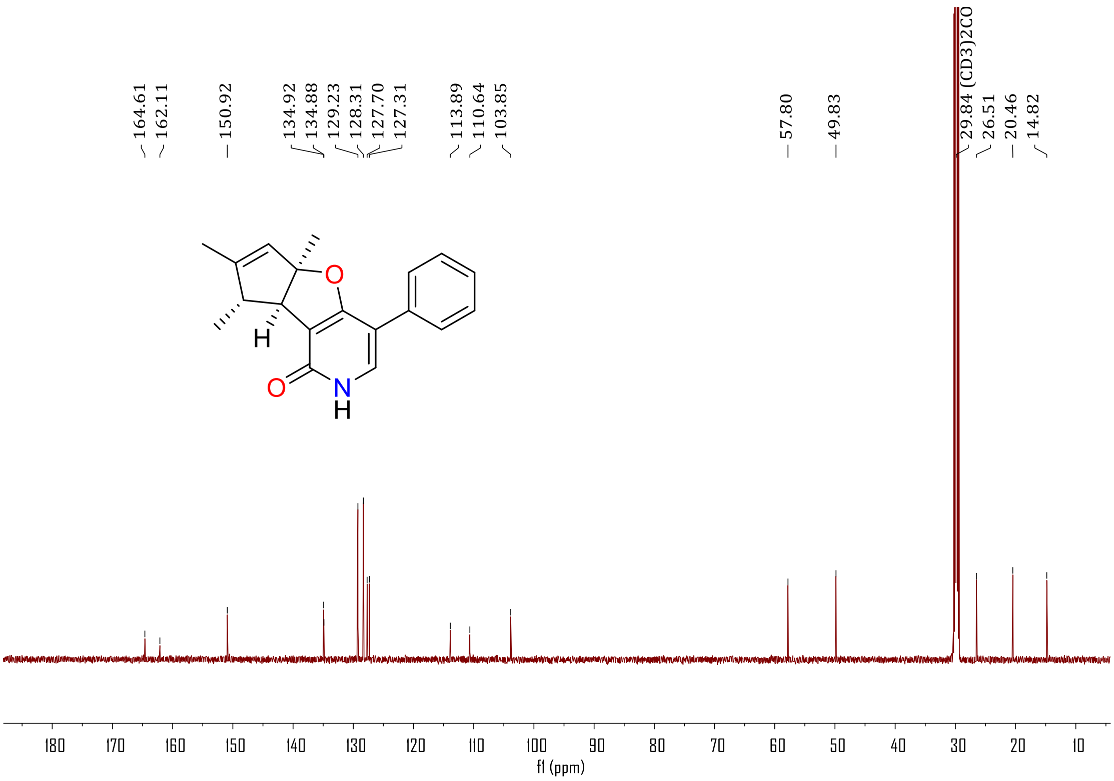

Figure S92. ^{13}C NMR spectrum (150 MHz, CD_3OD) of **8b**

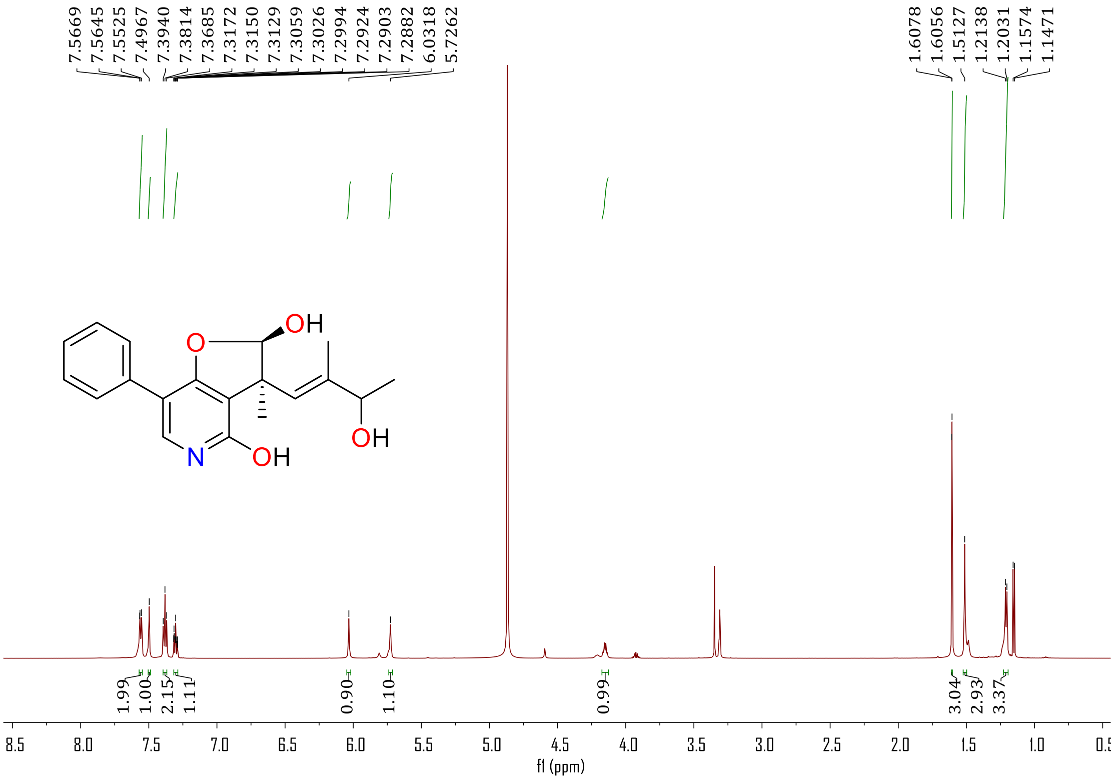

Figure S93. ^{1}H NMR spectrum (600 MHz, CD_3OD) of **9**

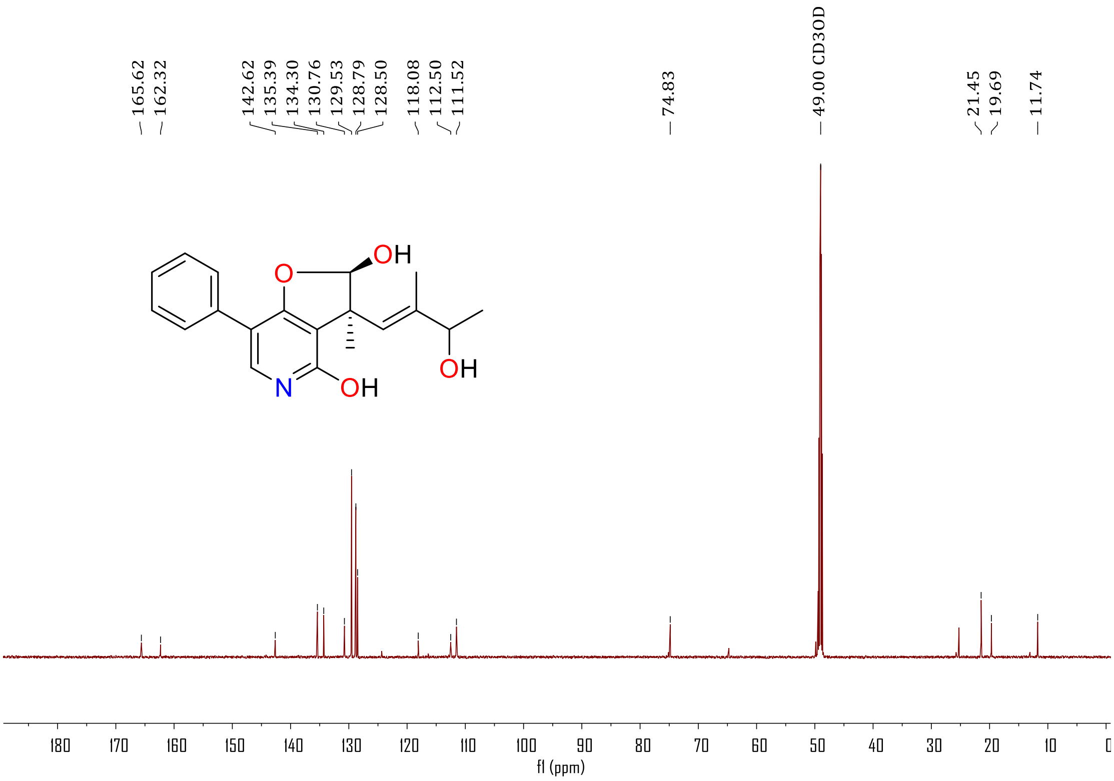

Figure S94. ^{13}C NMR spectrum (150 MHz, CD_3OD) of **9**
